# Isolation and characterization of three pairs of verrucosidin epimers from the marine sediment-derived fungus *Penicillium cyclopium* and configuration revision of penicyrone A and related analogues

**DOI:** 10.1007/s42995-023-00173-2

**Published:** 2023-05-28

**Authors:** Yan-He Li, Attila Mándi, Hong-Lei Li, Xiao-Ming Li, Xin Li, Ling-Hong Meng, Sui-Qun Yang, Xiao-Shan Shi, Tibor Kurtán, Bin-Gui Wang

**Affiliations:** 1 CAS and Shandong Province Key Laboratory of Experimental Marine Biology, Institute of Oceanology, Chinese Academy of Sciences, and Laboratory of Marine Biology and Biotechnology at the Qingdao National Laboratory for Marine Science and Technology, Qingdao, 266071 China; 2 School of Marine Science, University of Chinese Academy of Sciences, Beijing, 100049 China; 3 Department of Organic Chemistry, University of Debrecen, Egyetem Tér 1, Debrecen, 4032 Hungary; 4 Center for Ocean Mega-Science, Chinese Academy of Sciences, Qingdao, 266071 China

**Keywords:** Marine-derived fungus, *Penicillium cyclopium*, Verrucosidin derivatives, TDDFT-ECD calculations, Mosher’s method, Antimicrobial activity

## Abstract

Verrucosidins, a methylated α-pyrone class of polyketides rarely reported upon, have been implicated in one or more neurological diseases. Despite the significance of verrucosidins as neurotoxins, the absolute configurations of most of the derivatives have not been accurately characterized yet. In this study, three pairs of C-9 epimeric verrucosidin derivatives, including the known compounds penicyrones A and B (**1a/1b**) and 9-*O*-methylpenicyrones A and B (**2a/2b**), the new compounds 9-*O-*ethylpenicyrones A and B (**3a/3b**), together with the related known derivative verrucosidin (**4**), were isolated and identified from the culture extract of *Penicillium cyclopium* SD-413, which was obtained from the marine sediment collected from the East China sea. Their structures were established based on an in-depth analysis of nuclear magnetic resonances (NMR) and mass spectroscopic data. Determination of the absolute configurations of these compounds was accomplished by Mosher’s method and time-dependent density functional theory (TDDFT) calculations of electronic circular dichroism (ECD) and optical rotation (OR). The configurational assignment of penicyrone A demonstrated that the previously reported C-6 absolute configuration of verrucosidin derivatives needs to be revised from (6*S*) to (6*R*). The 9*R*/9*S* epimers of compounds **1–3** were found to exhibit growth inhibition against some pathogenic bacteria, indicating that they have potential as lead compounds for the creation of antimicrobial agents.

**Supplementary Information:**

The online version contains supplementary material available at 10.1007/s42995-023-00173-2.

## Introduction

According to numerous studies (Cao et al. 2020; Hai et al. 2021; Meng et al. 2021; Song et al. 2018; Wang et al. 2018; Zhou et al. 2022), marine-derived fungi have demonstrated to be a significant source of natural products with diverse chemical structures such as alkaloids, phenols, polyketides, terpenoids, and benzenes, which have remarkable potential as drug candidates in clinical applications (Carroll et al. 2022; EI-Kashef et al. 2019). Verrucosodin (**4**), the first example of a rarely reported class of methylated *α*-pyrone polyketide derivative, was initially isolated from *Penicillium verrucosum* var *cyclopium* in 1983 (Burka et al. 1983) and was found to possess the inhibitory activity for the expression of glucose-regulated protein 78 (GRP78) under strict hypoglycemic conditions and led to selective cell death of glucose-deprived HT-29 human colon cancer cells (Park et al. 2007). The importance of verrucosidin as a neurotoxin sparked a great deal of interest in characterizing its biosynthetic genes by clustered regularly interspaced short palindrome repeats (CRISPR) technology (Valente et al. 2021).

**Fig. 1:**
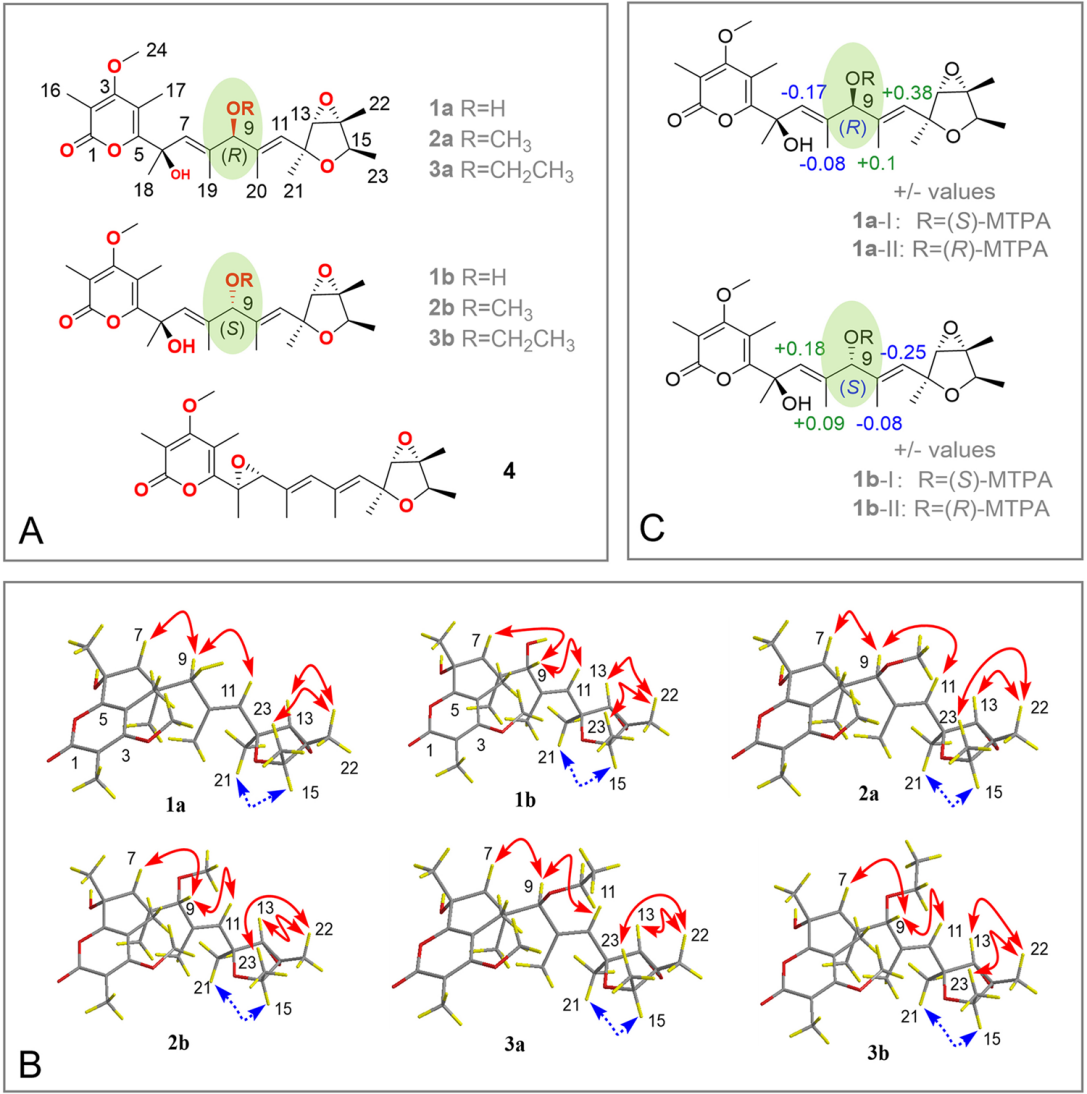
**A** Chemical structures of compounds **1–4**. **B** Key NOE correlations of compounds **1–3** (solid lines: *β*-orientation; dashed lines: *α*-orientation). **C** Δ*δ*_S–R_ values for Mosher esters (*S*)-**1a**/(*R*)-**1a** and (*S*)-**1b**/(*R*)-**1b**

As part of our attempts to discover naturally occurring compounds with distinctive structures and physiologically activities from marine-derived fungi (Chi et al. 2021; Du et al. 2022; Hu et al. 2021; Li et al. 2016, 2020; Yan et al. 2022), we conducted chemical investigations on the culture extract of *Penicillium cyclopium* SD-413, a fungus isolated from marine sediment that was collected from the East China Sea. As a result, three pairs of verrucosidin epimers, including the known compounds penicyrones A and B (**1a/1b**) and 9-*O-*methylpenicyrones A and B (**2a/2b**), the new compounds 9-*O-*ethylpenicyrones A and B (**3a/3b**), together with the related known derivatives verrucosidin (**4**) (Fig. 1A), were isolated and identified from the fungus *P. cyclopium* SD-413. The analysis of NMR data and ECD spectra were primarily used to clarify the planar structures and configurations of isolated compounds. Based on the configurational determination of penicyrone A (**1a**) by a combination of Mosher’s method and TDDFT calculations of ECD and OR, the absolute configurations at C-6 in the previously reported verrucosidin derivatives need to be revised (Bu et al. 2016). Antimicrobial properties against human- and aquatic-pathogenic bacteria, and plant-pathogenic fungi, were assessed for each isolated compound. In this work, the obtained compounds are described together with their isolation, structural identification, chiral HPLC resolution, stereochemical assignment, and biological activities.

## Results and discussion

The fermentation culture of *P. cyclopium* SD-413 was extensively extracted with EtOAc to produce an extract, which was then purified using column chromatography over Si gel, Lobar LiChroprep RP-18, Sephadex LH-20, and semi-preparative HPLC to yield compounds **1–4**. However, two sets of signals in the ^13^C NMR spectra of compounds **1–3** for chemical shifts of C-7 (Δ*δ* 0.5, 0.6, 0.6, respectively) through C-10 (Δ*δ* 0.2, 0.5, 1.3, respectively), C-11 (Δ*δ* 0.7, 1.0, 1.0, respectively), C-13 (Δ*δ* 0.1, 0.2, 0.1, respectively), C-19 (Δ*δ* 0.5, 0.4, 0.5, respectively), and C-20 (Δ*δ* 0.4, 0.3, 0.4, respectively) were observed (Table 1), indicating the presence of three pairs of epimers for compounds **1–3**. Each of the epimeric mixtures was further separated using chiral HPLC to successfully obtain individual epimeric isomers **1a** and **1b**, **2a** and **2b**, and **3a** and **3b**.

**Table 1:** ^1^H (500 MHz) and ^13^C (125 MHz) NMR spectroscopic data for compounds **1a**, **1b**, **2a**, **2b** and **4** in DMSO-*d*_6_

Position	**1a**		**1b**		**2a**		**2b**		**4**	
	*δ*_H_ (*J* in Hz)	*δ*_C_	*δ*_H_ (*J* in Hz)	*δ*_C_	*δ*_H_ (*J* in Hz)	*δ*_C_	*δ*_H_ (*J* in Hz)	*δ*_C_	*δ*_H_ (*J* in Hz)	*δ*_C_
1		163.9		163.9		163.8		163.8		163.8
2		108.6		108.6		108.6		108.6		109.4
3		168.8		168.8		168.7		168.7		167.2
4		110.1		110.0		110.1		110.1		109.9
5		161.3		161.2		161.0		160.9		155.1
6		73.3		73.2		73.2		73.2		59.8
7	5.76 (1H, s)	131.8	5.72 (1H, s)	132.3	5.76 (1H, s)	133.9	5.75 (1H, s)	134.5	3.66 (1H, s)	64.3
8		137.9		137.9		132.5		132.4		128.1
9	4.08 (1H, d, 4.2)	79.9	4.09 (1H, d, 4.0)	80.0	3.70 (1H, s)	89.4	3.72 (1H, s)	89.4	5.77 (1H, s)	130.6
10		135.2		135.4		134.8		135.3		133.7
11	5.59 (1H, s)	128.4	5.61 (1H, s)	127.7	5.59 (1H, s)	130.2	5.60 (1H, s)	129.2	5.52 (1H, s)	133.0
12		79.4		79.4		79.3		79.3		79.4
13	3.54 (1H, s)	66.3	3.55 (1H, s)	66.4	3.55 (1H, s)	66.2	3.56 (1H, s)	66.4	3.63 (1H, s)	66.3
14		66.9		66.9		66.9		66.9		66.9
15	3.93 (1H, q, 6.7)	75.7	3.93 (1H, q, 6.7)	75.7	3.95 (1H, q, 6.8)	75.7	3.93 (1H, q, 6.7)	75.7	3.99 (1H, q, 6.7)	75.9
16	1.92 (3H,s)	10.0	1.92 (3H, s)	10.0	1.90 (3H, s)	10.0	1.90 (3H, s)	10.0	1.93 (3H, s)	10.1
17	1.98 (3H, s)	10.3	1.98 (3H, s)	10.3	1.96 (3H, s)	10.3	1.96 (3H, s)	10.3	1.97 (3H, s)	9.3
18	1.49 (3H, s)	27.2	1.51 (3H, s)	27.3	1.48 (3H, s)	27.2	1.50 (3H, s)	27.3	1.39 (3H, s)	14.8
19	1.21 (3H, s)	12.3	1.22 (3H, s)	11.8	1.18 (3H, s)	12.0	1.18 (3H, s)	11.6	1.89 (3H, s)	15.4
20	1.58 (3H, s)	13.5	1.58 (3H, s)	13.9	1.56 (3H, s)	13.4	1.57 (3H, s)	13.7	1.92 (3H, s)	18.2
21	1.20 (3H, s)	21.9	1.20 (3H, s)	22.0	1.18 (3H, s)	21.9	1.18 (3H, s)	21.9	1.27 (3H, s)	21.9
22	1.34 (3H, s)	13.2	1.35 (3H, s)	13.5	1.32 (3H, s)	13.2	1.33 (3H, s)	13.4	1.35 (3H, s)	13.4
23	1.00 (3H, d, 6.7)	18.7	1.00 (3H, d, 6.7)	18.6	1.00 (3H, d, 6.7)	18.6	0.98 (3H, d, 6.7)	18.7	1.10 (3H, d, 6.7)	18.6
24	3.76 (3H, s)	60.3	3.76 (3H, s)	60.3	3.74 (3H, s)	60.3	3.74 (3H, s)	60.3	3.82 (3H, s)	60.5
25					3.10 (3H, s)	55.3	3.11 (3H, s)	55.2		
26										
6-OH	5.52 (1H, s)		5.53 (1H, s)		5.59 (1H, s)		5.60 (1H, s)			
9-OH	5.00 (1H, d, 4.2)		5.01 (1H, d, 4.2)							

Compound **1** was initially obtained as yellowish oil. Based on the positive HRESIMS data, the identified molecular formula was C_24_H_34_O_7_, indicating eight degrees of unsaturation. The NMR spectral data of compound **1** (Table 1) were found to be almost identical to those of penicyrones A and B, which were a pair of C-9 epimers and separated by repeated HPLC with the eluent CH_3_OH-H_2_O (3:2) (Bu et al. 2016). The relative configurations of C-6 and C-9 were determined by NOESY experiments in the literature, and the absolute configurations of penicyrones A (**1a**) and B (**1b**) were assigned by comparison of their OR values to those of known related compounds, as well as by biosynthetic considerations. Although the experimental ECD spectra were recorded, no ECD calculations were performed to verify these results. As the hydroxy groups or methyl groups at C-6, C-9 and C-12 are not sufficiently bulky to suppress the free rotations of the related single bonds in compounds **1a** and **1b**, the correlation of the C-6 and C-9 chirality centers had large uncertainty. Thus the absolute configurations of C-6 and C-9 in penicyrones A (**1a**) and B (**1b**) were determined independently in the present work by the Mosher’s method and TDDFT-ECD and -OR calculations.

The relative configuration of the tetrahydrofuran ring of compound **1a** was established by the key NOESY enhancements from H_3_-22 to H-13 and H_3_-23 and from H-15 to H_3_-21. Analysis of the NOEs from H-9 to H-7 and H-11 revealed that the geometries of double bonds at C-7 and C-10 were *E* (Fig. 1B).

The absolute configuration of C-6 of compound **1a** was determined by the solution TDDFT-ECD method (Mándi et al. 2019). In a 21 kJ/mol energy window, the Merck Molecular Force Field (MMFF) conformational search was performed on the arbitrarily selected (6*S*, 9*R*,12*S*,13*S*,14*R*,15*R*)-**1a**, which resulted in 3038 conformer clusters. The *α*-pyrone chromophore and its contiguous C-6 chirality center govern the ECD spectrum (Suárez-Ortiz et al. 2017), and to analyze fewer conformers, an AM1 level re-optimization was carried out before the DFT reoptimization (Csupor et al. 2020), and reclustering was applied to neglect the rotation of the flexible side-chain and the condensed tetrahydrofuran ring beyond C-9 (Zhou et al. 2014). The resulting 87 conformers were re-optimized at the ωB97X/TZVP (Chai et al. 2008) PCM/MeCN and ωB97X/TZVP PCM/MeOH levels for the subsequent ECD and OR calculations, respectively. The TDDFT-ECD calculations performed at four different levels for conformers over 1% Boltzmann-population gave good mirror-image agreement with the experimental spectrum indicating (6*R*) absolute configuration, opposite to that reported in the literature (Supplementary Fig. S46).

To further verify the absolute configuration of C-6, OR calculations (Polavarapu et al. 2008; Sun et al. 2016) were also carried out at four different levels of theory for the ωB97X/TZVP PCM/MeOH conformers. The B3LYP/TZVP PCM/MeOH, BH&HLYP/TZVP PCM/MeOH, CAM-B3LYP/TZVP PCM/MeOH and PBE0/TZVP PCM/MeOH calculations gave large negative OR values ranging from 78 to 83 for (6*S*,9*R*,12*S*,13*S*,14*R*,15*R*)-**1a** [experimental value: [α]_D_^25^ + 110 ( *c* 0.1, MeOH)] (Supplementary Table S1). In contrast to the ECD calculations, the OR calculation was not decisive for the determination of the C-6 chirality center even with the good agreement in hand, since the contribution of the other five chirality centers to the OR was not known. The (6*S*) absolute configuration of **1a** described in the literature was confirmed to be wrong (Bu et al. 2016). It is important to note that Bu et al. applied a low-level conformational analysis to correlate the experimental NOE peaks with the distances of protons and determine the relative configuration of C-6 and C-9. The relative configuration of the C-6 and C-9 centers was determined on the basis of the H-7/H_3_-18 NOE correlation, which was feasible for both the (6*R*) and (6*S*) epimers, and hence it is not decisive.

The absolute configuration at C-9 was established by the modified Mosher’s method (Seco et al. 2012). The Δ*δ* values obtained for the (*S*)- and (*R*)-MTPA esters [(*R*)-**1a** and (*S*)-**1a**, respectively] of **1a** allowed determining the absolute configuration of C-9 as (*R*) (Fig. 1C).

With the consideration of the biogenetic origin and co-isolation of verrucosidin (**4**), whose absolute configuration was determined by single crystal X-ray diffraction (Burka et al. 1983), the chirality of C-12 in **1a** was suggested to be (*S*), the same as that in compound 4 (Burka et al. 1983). Finally, the absolute configuration of compound **1a** was established as (6*R*, 9*R*,12*S*,13*S*,14*R*,15*R*).

The NMR and positive HRESIMS data of compound **1b** were almost identical to those of **1a** (Table 1), indicating that they share the same planar structure. Key NOESY enhancements in compound **1b** from H_3_-22 to H-13 and H_3_-23 and from H-15 to H_3_-21 assigned **1b** possessing the same relative configuration of the tetrahydrofuran ring as that in compound **1a**. The geometries of the double bonds at C-7 and C-10 were also confirmed to be *E* by analysis of the NOEs from H-9 to H-7 and H-11, which were also well matched with those of compound **1a** (Fig. 1B).

The experimental ECD curve of compound **1b** well matched that of compound **1a** (Supplementary Fig. S10), suggesting that the absolute configuration of C-6 in compound **1b** was also (*R*). By applying the modified Mosher's method, the absolute configuration of C-9 was determined to be (*S*) (Seco et al. 2012) (Fig. 1C). Considering the biosynthetic pathway, the absolute configuration of compound **1b** was assigned as (6*R*,9*S*,12*S*,13*S*,14*R*,15*R*).

Compound** 2** was also obtained as a yellowish oil. According to the positive HRESIMS data, its molecular formula was determined to be C_25_H_36_O_7_ (eight unsaturations). The NMR spectral data of compound** 2** (Table 1) were found to be nearly identical to those of methyl penicyrone, which was isolated as a mixture of two epimers being epimeric at C-9 (Pan et al. 2018).

In the present work, methyl penicyrone (**2**) was purified by chiral HPLC to yield the optically active derivatives compounds **2a** and **2b** (Supplementary Fig. S30). The NOESY and NMR data of compounds **2a** and **2b** were well matched to that of compounds **1a** and **1b** (Fig. 1B and Table 1). The double bonds at C-7 and C-10 were also deduced as *E* based on NOEs from H-9 to H-7 and H-11 (Fig. 1B). Since compounds **1b**, **2a** and **2b** had similar ECD curves, the absolute configuration of C-6 is expected to be the same.

The absolute configurations of C-9 in compounds **2a** and **2b** were confirmed by NMR data and chiral HPLC analysis, in which the (9*S*)-epimer possessed a larger retention time than that of the (9*R*)-epimer, as also observed for compounds **1a** and **1b** (Table 1 and Supplementary Fig. S30). Considering the biosynthetic pathway, the absolute configurations of compounds **2a** and **2b** were elucidated as (6*R*,9*R*,12*S*,13*S*,14*R*,15*R*) and (6*R*,9*S*,12*S*,13*S*,14*R*,15*R*), respectively.

Compound **3** was also obtained as a yellowish oil and was successfully separated by chiral HPLC to yield two epimers **3a** and **3b** (Supplementary Fig. S47).

Based on the positive HRESIMS data, compound** 3a** had the molecular formula of C_26_H_38_O_7_ (eight degrees of unsaturation). The ^1^H, ^13^C, and HSQC NMR data of compound** 3a** (Table 2) displayed resonances of 10 methyls (including one methoxy), one oxygenated methylene, five methines (including two olefinic and three oxygenated), and 10 nonprotonated (including one carbonyl, six olefinic and three sp^3^ oxygenated) carbons, and one exchangeable proton (6-OH). Compound** 3a** was found to be a derivative of verrucosidin that was comparable to compound **1** by thorough investigation of the ^1^H and ^13^C NMR spectroscopic data (Bu et al. 2016). However, the -OH group at C-9 in compound **1** was changed to an -OCH_2_CH_3_ group in compound **3a**, and COSY and HMBC experiments which were shown in Supplementary Fig. S48 corroborated this deduction. Thus, it was established that compound** 3a** had the planar structure as depicted in Fig. 1A.

**Table 2:** ^1^H (500 MHz) and ^13^C (125 MHz) NMR spectroscopic data for compounds **3a** and **3b** in DMSO-*d*_6_

Position	**3a**		**3b**	
	*δ*_H_ (*J* in Hz)	*δ*_C_	*δ*_H_ (*J* in Hz)	*δ*_C_
1		163.8		163.8
2		108.6		108.6
3		168.7		168.7
4		110.0		110.0
5		161.1		161.0
6		73.2		73.2
7	5.79 (1H, s)	133.5	5.77 (1H, s)	134.1
8		132.9		132.9
9	3.83 (1H, s)	87.6	3.85 (1H, s)	87.6
10		135.3		135.6
11	5.61 (1H, s)	130.0	5.62 (1H, s)	129.0
12		79.3		79.3
13	3.57 (1H, s)	66.2	3.58 (1H, s)	66.3
14		66.9		66.9
15	3.95 (1H, q, 6.6)	75.7	3.95 (1H, q, 6.6)	75.7
16	1.92 (3H, s)	10.0	1.92 (3H, s)	10.0
17	1.98 (3H, s)	10.3	1.97 (3H, s)	10.3
18	1.50 (3H, s)	27.2	1.51 (3H, s)	27.3
19	1.20 (3H, s)	12.3	1.21 (3H, s)	11.8
20	1.58 (3H, s)	13.4	1.59 (3H, s)	13.8
21	1.20 (3H, s)	21.9	1.20 (3H, s)	21.9
22	1.35 (3H, s)	13.2	1.35 (3H, s)	13.4
23	0.99 (3H, d, 6.7)	18.6	0.99 (3H, d, 6.7)	18.6
24	3.76 (3H, s)	60.3	3.76 (3H, s)	60.3
25	3.29 (2H, q, 7.0)	62.7	3.30 (2H, q, 7.0)	62.6
26	1.1 (3H, t, 7.0)	15.3	1.1 (3H, t, 7.0)	15.2
6-OH	5.61 (1H, s)		5.62 (1H, s)	

Based on the positive HRESIMS data, the molecular formula of compound** 3b** was confirmed as C_26_H_38_O_7_ (8 degrees of unsaturation), same as that of **3a**. The NMR spectra for **3a** and **3b** were almost identical, which showed they share the same planar structure.

The NOESY and NMR data of compounds **3a** and **3b** matched well with that of compound **1a** (Fig. 1B). The ECD behaviors of compounds **3a** and **3b** were similar to those of compounds **1a** and **1b** (Supplementary Fig. S10 and S46), indicating that their absolute configurations at C-6 were both (*R*), the same as that of compound **1a**. Considering the NMR data, retention time in chiral HPLC chromatograms, and the biosynthetic pathway, the absolute configurations of compounds **3a** and **3b** were tentatively determined to be (6*R*,9*R*,12*S*,13*S*,14*R*,15*R*) and (6*R*,9*S*,12*S*,13*S*,14*R*,15*R*), respectively, and the geometry of C-7 and C-10 in these two compounds were also confirmed to be *E* based on NOEs as shown in Fig. 1B.

All of the isolated pure epimers were evaluated for antibiotic efficacy against one human pathogenic bacterium (*Escherichia coli* EMBLC-1), 10 aquatic pathogens (*Aeromonas hydrophila* QDIO-1, *Edwardsiella ictaluri* QDIO-9, *E. tarda* QDIO-2, *Micrococcus luteus* QDIO-3, *Pseudomonas aeruginosa* QDIO-4*, Vibrio alginolyticus* QDIO-5, *V. anguillarum* QDIO-6*, V. harveyi* QDIO-7, *V. parahemolyticus* QDIO-8, and *V. vulnificus* QDIO-10), and seven plant-pathogenic fungi (*Bipolaris sorokiniana* QDAU-3, *Ceratobasidium cornigerum* QDAU-6, *Colletotrichum glecosporioides* QDAU-2, *Fusarium graminerum* QDAU-4, *F. oxysporum* QDAU-5, *Penicillium digitatum* QDAU-14 and *Physalospora piricola Nosa* QDAU-15). As shown in Table 3, the epimer **2a** showed activity against human pathogenic bacterium *E. coli* and aquatic pathogens *P. aeruginosa* and *Ed. ictaluri*, with MIC values of 4, 8, and 8 μg/mL, respectively, while **2b** showed moderate inhibitory activities against *E. coli* with an MIC value of 8 μg/mL. Epimers **3a** and **3b** showed activity against *A. hydrophilia*, each with an MIC value of 8 μg/mL.

**Table 3:** Antibacterial activities of compounds 1–4 (MIC, μg/mL)

Compound	*Escherichia coli*	*Pseudomonas aeruginosa*	*Vibrio harveyi*	*Edwardsiella ictaluri*	*Aeromonas hydrophilia*
**1**	64	–	64	16	–
**1a**	64	–	64	16	64
**1b**	64	–	64	16	–
**2**	8	16	–	16	–
**2a**	4	8	64	8	–
**2b**	8	16	–	–	–
**3**	–	–	–	16	8
**3a**	–	64	–	16	8
**3b**	64	–	–	8	8
**4**	64	64	16	–	–
Chloramphenicol	1	1	0.5	1	1

## Conclusion

In summary, three pairs of verrucosidin derivatives (**1–3**) representing an uncommonly described class of methylated α-pyrone polyketides, together with one related known derivative verrucosidin (**4**), were isolated from the culture extract of *Penicillium cyclopium* SD-413 obtained from the marine sediment. The optically pure epimers for each mixed epimers were successfully separated by chiral HPLC resolution, allowing the absolute configuration to be determined by Mosher’s method and TDDFT-calculations and comparisons. The epimer **2a** showed to be more effective than epimer **2b** in inhibiting one human- and two aquatic-pathogens, with MIC values ranging from 4 to 8 g/mL. This information may be helpful in the development of new antibacterial agents.

## Material and methods

### General experimental procedures

The instruments and chemical materials employed in this investigation are the same as those used in our earlier work (Du et al. 2022; Hu et al. 2021).

### Fungal material

The fungus *Penicillium cyclopium* SD-413 was obtained from a marine sediment sample, which was collected from the East China Sea in May 2017. It was identified by DNA amplification and the sequencing of the ITS region utilizing a molecular biological procedure described in our prior research (Wang et al. 2006). GenBank has received the sequenced data of the fungal strain (accession no. MN818582.1). The sequence was most similar (99%) to the sequence of *Penicillium cyclopium* (accession no. MT990551.1), according to the results of a BLAST search.

### Fermentation

The fresh mycelia of *P. cyclopium* were grown on PDA medium at 28 °C for five days and then were inoculated into 1 L conical flasks (100 flasks) with solid rice medium at room temperature for 30 days. Each flask contained 70 g of rice, 0.1 g of corn flour, 0.3 g of peptone, 0.1 g of sodium glutamate, and 100 mL naturally sourced and filtered seawater, which was obtained from the Huiquan Gulf of the Yellow Sea near the campus of IOCAS.

### Extraction and isolation

The fermented cultures (98 flasks) were extracted four times with EtOAc, and the solvents were then evaporated under reduced pressure to afford an extract (71.2 g), which was fractionated using vacuum liquid chromatography (VLC) on silica gel while eluting with various solvents of increasing polarity from petroleum ether (PE) to MeOH to obtain nine fractions (Frs. 1–9). Fr. 5 (6.65 g), eluted with PE-EtOAc (2:1), was further purified by column chromatography (CC) over Lobar LiChroprep RP-18 with a MeOH-H_2_O gradient (from 10:90 to 100:0) to afford 10 subfractions (Fr. 5.1–5.10). Fr. 5.4 (610 mg) was further purified by CC on silica gel eluting with a CH_2_Cl_2_-MeOH gradient (from 100:1 to 20:1) and then by CC on Sephadex LH-20 (MeOH) to yield compounds **1** (14.6 mg) and **2** (32.1 mg). The optical resolution of compound **1** by chiral HPLC (CHIRALPAK IG column, *n*-hexane–isopropanol 75:25, 1.0 mL/min) afforded optical pure epimers **1a** (3.1 mg) and **1b** (5.9 mg); whereas, the optical resolution of compound **2** by chiral HPLC (CHIRALPAK IG column, *n*-hexane–isopropanol 85:15, 1.0 mL/min) afforded optical pure epimers **2a** (5.6 mg) and **2b** (3.1 mg). Purification of Fr. 5.6 (596 mg) by CC on silica gel eluting with a CH_2_Cl_2_-MeOH gradient (from 150:1 to 100:1) and by preparative TLC (pTLC) (plate: 20 cm × 20 cm, developing solvents: PE-acetone 5:1) and then by CC on Sephadex LH-20 (MeOH) yielded the mixture of compound **3** (50.4 mg), which was further separated by chiral HPLC (CHIRALPAK IG column, *n*-hexane–isopropanol 80:20, 1.0 mL/min) to afford optical pure isomers **3a** (7.2 mg) and **3b** (6.9 mg).

### Penicyrone A (**1a**)

Yellowish oil; [α]_D_^25^ + 110 (*c* 0.1, MeOH); UV (MeOH) *λ*_max_ (log *ε*) 300 (3.96), 203 (4.42) nm; ECD (1.87 × 10^–4^ M, MeCN) *λ*_max_ (Δ*ε*) 297 (+ 9.07), 212 (–27.07); ECD (3.94 × 10^–4^ M, MeOH) *λ*_max_ (Δ*ε*) 298 (+ 12.78), 212 (–42.22); ^1^H and ^13^C NMR data, Table 1; HRESIMS *m*/*z* 435.2379 [M + H]^+^ (calcd for C_24_H_35_O_7_, 435.2377).

### Penicyrone B (**1b**)

Yellowish oil; [α]_D_^25^ + 100 (*c* 0.1, MeOH); UV (MeOH) *λ*_max_ (log *ε*) 300 (3.80), 203 (4.24) nm; ECD (2.73 × 10^–4^ M, MeCN) *λ*_max_ (Δ*ε*) 299 (+ 7.65), 211 (–24.34); ECD (3.82 × 10^–4^ M, MeOH) *λ*_max_ (Δ*ε*) 300 (+ 11.77), 213 (–40.24); ^1^H and ^13^C NMR data, Table 1; HRESIMS *m*/*z* 435.2368 [M + H]^+^ (calcd for C_24_H_35_O_7_, 435.2377).

### (9*R*)*-O-*Methylpenicyrone (**2a**)

Yellowish oil; [α]_D_^25^ + 104 (*c* 0.1, MeOH); UV (MeOH) *λ*_max_ (log *ε*) 300 (3.99), 203 (4.43) nm; ECD (2.51 × 10^–4^ M, MeCN) *λ*_max_ (Δ*ε*) 297 (+ 10.89), 213 (–32.57); ECD (3.79 × 10^–4^ M, MeOH) *λ*_max_ (Δ*ε*) 298 (+ 11.90), 214 (–37.50); ^1^H and ^13^C NMR data, Table 1; HRESIMS *m*/*z* 449.2539 [M + H]^+^ (calcd for C_25_H_37_O_7_, 449.2534).

### (9*S*)*-O-*Methylpenicyrone (**2b**)

Yellowish oil; [α]_D_^25^ + 92 (*c* 0.1, MeOH); UV (MeOH) *λ*_max_ (log *ε*) 300 (4.01), 203 (4.46) nm; ECD (2.51 × 10^–4^ M, MeCN) *λ*_max_ (Δ*ε*) 298 (+ 10.07), 213 (–29.62); ECD (3.63 × 10^–4^ M, MeOH) *λ*_max_ (Δ*ε*) 299 (+ 10.44), 214 (–32.11); ^1^H and ^13^C NMR data, Table 1; HRESIMS *m*/*z* 449.2539 [M + H]^+^ (calcd for C_25_H_37_O_7_, 435.2534).

### (9*R*)*-O-*Ethylpenicyrone (**3a**)

Yellowish oil; [α]_D_^25^ + 90 (*c* 0.1, MeOH); UV (MeOH) *λ*_max_ (log *ε*) 300 (3.79), 203 (4.24) nm; ECD (3.22 × 10^–4^ M, MeOH) *λ*_max_ (Δ*ε*) 299 (+ 10.50), 215 (–32.26); ^1^H and ^13^C NMR data, Table 1; HRESIMS *m*/*z* 463.2688 [M + H]^+^ (calcd for C_26_H_39_O_7_, 463.2690).

### (9*S*)*-O-*Ethylpenicyrone (**3b**)

Yellowish oil; [α]_D_^25^ + 75 (*c* 0.1, MeOH); UV (MeOH) *λ*_max_ (log *ε*) 300 (4.00), 203 (4.44), nm; ECD (3.22 × 10^–4^ M, MeOH) *λ*_max_ (Δ*ε*) 295 (+ 10.92), 214 (–33.04); ^1^H and ^13^C NMR data, Table 1; HRESIMS *m*/*z* 463.2677 [M + H]^+^ (calcd for C_26_H_39_O_7_, 463.2690).

### Preparation of MTPA esters of **1a** and **1b**

Preparation of the (*R*)- and (*S*)-MTPA esters of **1a** and **1b**. To the stirred solutions of **1a** (1.1 mg) and **1b** (1.2 mg) in pyridine (400 μL), respectively, each were given an addition of 2.0 mg of 4-(dimethylamino) pyridine and 10 μL of (*S*)-( +)-α-methoxy-α-(trifluoromethyl) phenylacetyl (MTPA). The mixtures were stirred at 25 ℃ for 12 h before adding 0.2 mL of H_2_O to cease the reactions. The reaction mixtures were subjected to pTLC with CH_2_Cl_2_-MeOH (20:1) as developing solvents to obtain the (*R*)-Mosher ester *R*-**1a** (0.8 mg) and *R*-**1b** (0.9 mg). Treatment of **1a** (1.0 mg) and **1b** (1.3 mg) with (*R*)-MTPA (10 μL) with the same procedure was used to obtain the corresponding (*S*)-Mosher ester *S*-**1a** (0.8 mg) and *S*-**1b** (1.0 mg) (see Supplementary data for NMR data) (Suárez-Ortiz et al. 2017).

### Antimicrobial assay

The microplate assay with three repetitions was used to evaluate the effectiveness of the antibiotics against human pathogenic bacteria, aquatic pathogens, and plant-pathogenic fungi (Pierce et al. 2008). The Institute of Oceanology, Chinese Academy of Sciences (IOCAS) provided the pathogenic bacterial and aquatic pathogenic strains, and Qingdao Agricultural University provided the plant pathogenic fungal strains. Positive controls against bacteria and fungi were chloramphenicol and amphotericin B, respectively.

### Computational section

Mixed torsional/low-mode conformational searches were carried out with the Macromodel 10.8.011 software using the MMFF with an implicit solvent model for CHCl_3_ applying a 21 kJ/mol energy window (MacroModel. 2015). Geometry reoptimizations of the resultant conformers (AM1, and ωB97X/TZVP with PCM solvent model for MeCN and MeOH), TDDFT-ECD and OR calculations were performed with Gaussian 09 software package (Frisch et al. 2013). Distance based reclustering was performed with a 0.5 Å cut off for the non-hydrogen atoms plus the 6-OH hydrogen taking into account the left part of the molecule until C-9. For the ECD and OR calculations the B3LYP/TZVP, BH&HLYP/TZVP, CAM-B3LYP/TZVP, and PBE0/TZVP levels were applied with the same solvent model as in the preceding DFT optimization level. ECD spectra were generated as the sum of Gaussians with 3000 cm^−1^ half-height widths, using dipole-velocity-computed rotational strength values (Stephens et al. 2010). Boltzmann distributions were estimated from the ωB97X energies. The MOLEKEL program was used for visualization of the results (Varetto et al. 2009).

## Supplementary Information

Below is the link to the electronic supplementary material.

Supplementary file1 (DOC 9009 KB)

## Data Availability

The data that supports the findings of this study are included in this published article (and its supplementary information file).
