# Supplementary material for: Isolation and characterization of three pairs of verrucosidin epimers from the marine sediment-derived fungus Penicillium cyclopium and configuration revision of penicyrone A and related analogues
Source: Mar Life Sci Technol. 2023 May 28;5(2):223–31. doi: 10.1007/s42995-023-00173-2 (PMC10232390; doi:10.1007/s42995-023-00173-2)
Supplement: Supplementary file 1 — Supplementary file1 (DOC 9009 KB) [file 42995_2023_173_MOESM1_ESM.doc]

Supplementary Information

**Isolation and characterization of three pairs of** **verrucosidin epimers from the marine sediment-derived fungus *Penicillium cyclopium* and configuration revision of penicyrone A and related analogues**

**Yan-He Li ^1,2^, Attila Mándi ^3^, Hong-Lei Li ^1,4^, Xiao-Ming Li ^1,4^, Xin Li ^1,4^, Ling-Hong Meng ^1,4^, Sui-Qun Yang ^1,4^, Xiao-Shan Shi ^1,4^, Tibor Kurtán ^3^,*, and Bin-Gui Wang ^1,2,4^,***

^1^. CAS and Shandong Province Key Laboratory of Experimental Marine Biology, Institute of Oceanology, Chinese Academy of Sciences, and Laboratory of Marine Biology and Biotechnology at the Qingdao National Laboratory for Marine Science and Technology, Nanhai Road 7, Qingdao 266071, China

^2^. School of Marine Science, University of Chinese Academy of Sciences, Yuquan Road 19A, Beijing 100049, China

^3^. Department of Organic Chemistry, University of Debrecen, Egyetem tér 1, Debrecen 4032, Hungary

^4^. Center for Ocean Mega-Science, Chinese Academy of Sciences, Nanhai Road 7, Qingdao 266071, China.

***** Correspondence: [kurtan.tibor@science.unideb.hu](mailto:kurtan.tibor@science.unideb.hu); wangbg@ms.qdio.ac.cn

Content

| **Fig. S1.** | HRESIMS spectrum of compound **1a**; |
| --- | --- |
| **Fig. S2.** | ^1^H NMR spectrum of compound **1a** (Recorded in DMSO-*d*_6_); |
| **Fig. S3.** | ^13^C NMR and DEPT spectra of compound **1a** (Recorded in DMSO-*d*_6_); |
| **Fig. S4.** | ^1^H-^1^H COSY spectrum of compound **1a** (Recorded in DMSO-*d*_6_); |
| **Fig. S5.** | NOESY spectrum of compound **1a** (Recorded in DMSO-*d*_6_); |
| **Fig. S6.** | ^1^H NMR spectrum of the (*S)*-MTPA ester of compound **1a** (Recorded in DMSO-*d*_6_);  of compound **4** (4a) |
| **Fig. S7.** | ^1^H NMR spectrum of the (*R)*-MTPA ester compound **1a** (Recorded in DMSO-*d*_6_); |
| **Fig. S8.** | Low-energy (≥ 1%) ωB97X/TZVP PCM/MeCN conformers of (6*S*,9*R*,12*S*,13*S*,14*R*,15*R*)-**1a**. |
| **Fig. S9.** | Low-energy (≥ 1%) ωB97X/TZVP PCM/MeOH conformers of (6*S*,9*R*,12*S*,13*S*,14*R*,15*R*)-**1a**. |
| **Fig. S10.** | Experimental ECD spectra of **1a**–**3b** measured in MeOH. |
| **Fig. S11.** | Experimental ECD spectra of **1a**–**2b** measured in MeCN. |
| **Table S1.** | Boltzmann populations and specific optical rotations of the low-energy conformers of  (6*S*,9*R*,12*S*,13*S*,14*R*,15*R*)-**1a** computed at various levels for the ωB97X/TZVP PCM/MeOH  optimized MMFF conformers. |
| **Table S2.** | Cartesian coordinates and energies of the low-energy conformers calculated.  At the ωB97X/TZVP PCM/MeCN level of (6*S*,9*R*,12*S*,13*S*,14*R*,15*R*)-**1a**. |
| **Table S3.** | Cartesian coordinates and energies of the low-energy conformers calculated.  at the ωB97X/TZVP PCM/MeOH level of (6*S*,9*R*,12*S*,13*S*,14*R*,15*R*)-**1a**. |
| **Fig. S12.** | HRESIMS spectrum of compound **1b**; |
| **Fig. S13.** | ^1^H NMR spectrum of compound **1b** (Recorded in DMSO-*d*_6_); |
| **Fig. S14.** | ^13^C NMR and DEPT spectra of compound **1b** (Recorded in DMSO-*d*_6_); |
| **Fig. S15.** | ^1^H-^1^H COSY spectrum of compound **1b** (Recorded in DMSO-*d*_6_); |
| **Fig. S16.** | NOESY spectrum of compound **1b** (Recorded in DMSO-*d*_6_); |
| **Fig. S17.** | ^1^H NMR spectrum of the (*S)*-MTPA ester of compound **1b** (Recorded in DMSO-*d*_6_); |
| **Fig. S18.** | ^1^H NMR spectrum of the (*R)*-MTPA ester compound **1b** (Recorded in DMSO-*d*_6_); |
| **Fig. S19.** | Chiral HPLC chromatogram of compounds **1a/1b**; |
| **Fig. S20.** | HRESIMS spectrum of compound **2a**; |
| **Fig. S21.** | ^1^H NMR spectrum of compound **2a** (Recorded in DMSO-*d*_6_); |
| **Fig. S22.** | ^13^C NMR and DEPT spectra of compound **2a** (Recorded in DMSO-*d*_6_); |
| **Fig. S23.** | ^1^H-^1^H COSY spectrum of compound **2a** (Recorded in DMSO-*d*_6_); |
| **Fig. S24.**  **3.** | NOESY spectrum of compound **2a** (Recorded in DMSO-*d*_6_); |
| **Fig. S25.** | HRESIMS spectrum of compound **2b**; |
| **Fig. S26.** | ^1^H NMR spectrum of compound **2b** (Recorded in DMSO-*d*_6_); |
| **Fig. S27.** | ^13^C NMR and DEPT spectra of compound **2b** (Recorded in DMSO-*d*_6_); |
| **Fig. S28.** | ^1^H-^1^H COSY spectrum of compound **2b** (Recorded in DMSO-*d*_6_); |
| **Fig. S29.** | NOESY spectrum of compound **2b** (Recorded in DMSO-*d*_6_); |
| **Fig. S30.** | Chiral HPLC chromatogram of compounds **2a/2b**; |
| **Fig. S31.** | ^1^H NMR spectrum of compound **3** (Recorded in DMSO-*d*_6_); |
| **Fig. S32.** | ^13^C NMR and DEPT spectra of compound **3** (Recorded in DMSO-*d*_6_); |
| **Fig. S33.** | ^1^H-^1^H COSY spectrum of compound **3a** (Recorded in DMSO-*d*_6_); |
| **Fig. S34.** | HSQC spectrum of compound **3** (Recorded in DMSO-*d*_6_); |
| **Fig. S35.** | HMBC spectrum of compound **3** (Recorded in DMSO-*d*_6_); |
| **Fig. S36.** | HRESIMS spectrum of compound **3a**; |
| **Fig. S37.** | ^1^H NMR spectrum of compound **3a** (Recorded in DMSO-*d*_6_); |
| **Fig. S38.** | ^13^C NMR and DEPT spectra of compound **3a** (Recorded in DMSO-*d*_6_); |
| **Fig. S39.** | NOESY spectrum of compound **3a** (Recorded in DMSO-*d*_6_); |
| **Fig. S40.** | HRESIMS spectrum of compound **3b**; |
| **Fig. S41.** | ^1^H NMR spectrum of compound **3b** (Recorded in DMSO-*d*_6_); |
| **Fig. S42.** | ^13^C NMR and DEPT spectra of compound **3b** (Recorded in DMSO-*d*_6_); |
| **Fig. S43.** | NOESY spectrum of compound **3b** (Recorded in DMSO-*d*_6_); |
| **Fig. S44.** | ^1^H NMR spectrum of compound **4** (Recorded in DMSO-*d*_6_); |
| **Fig. S45.** | ^13^C NMR and DEPT spectra of compound **4** (Recorded in DMSO-*d*_6_). |
| **Fig. S46.** | Experimental ECD spectra of (6*R*,9*R*,12*S*,13*S*,14*R*,15*R*)-**1a** in MeCN compared with the Boltzmann-  weighted B3LYP/TZVP PCM/MeCN, BH&HLYP/TZVP PCM/MeCN, CAM-B3LYP/TZVP  PCM/MeCN and PBE0-TZVP PCM/MeCN spectra of (6*S*,9*R*,12*S*,13*S*,14*R*,15*R*)-**1a**. |
| **Fig. S47.** | Chiral HPLC chromatogram of compound **3** (Column: CHIRALPAK IG, 4.6 × 250 mm;  Temperature: 25 ^o^C; Flow rate: 1 mL/min; Mobile phase: 80% *n*-hexane-isopropanol). |
| **Fig. S48.** | COSY and key HMBC correlations of compound **3**. |

**Fig. S1.** HRESIMS spectrum of compound **1a**;


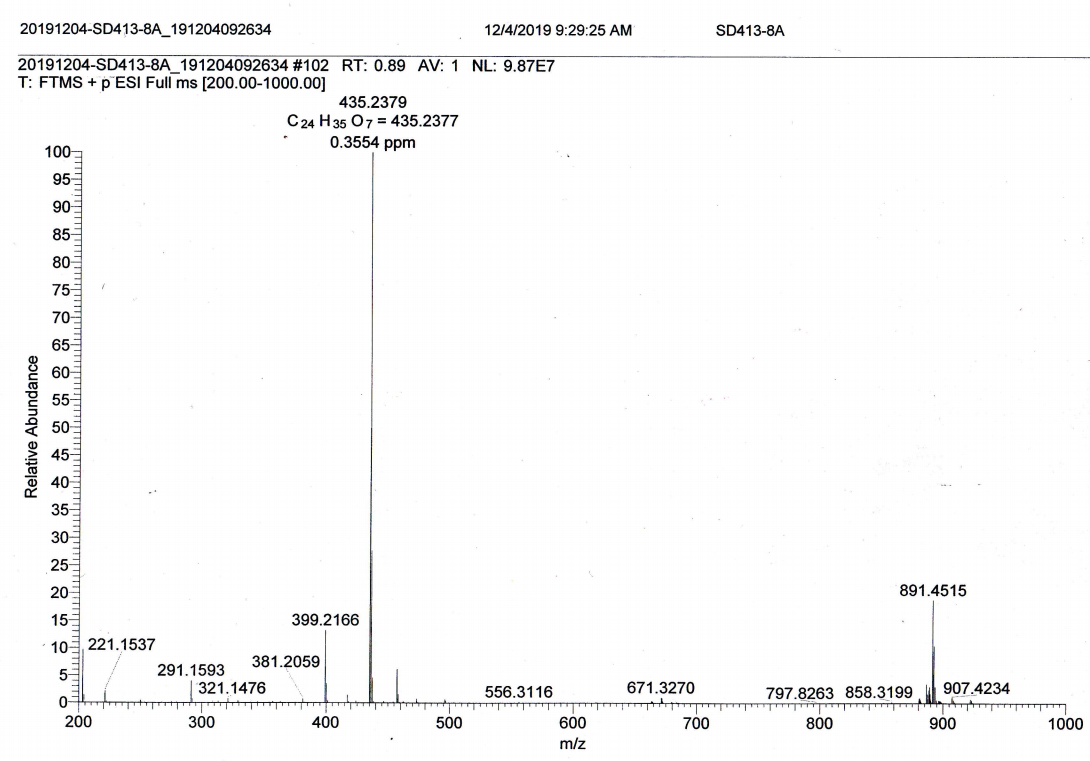


**Fig. S2.** ^1^H NMR spectrum of compound **1a** (Recorded in DMSO-*d*_6_);


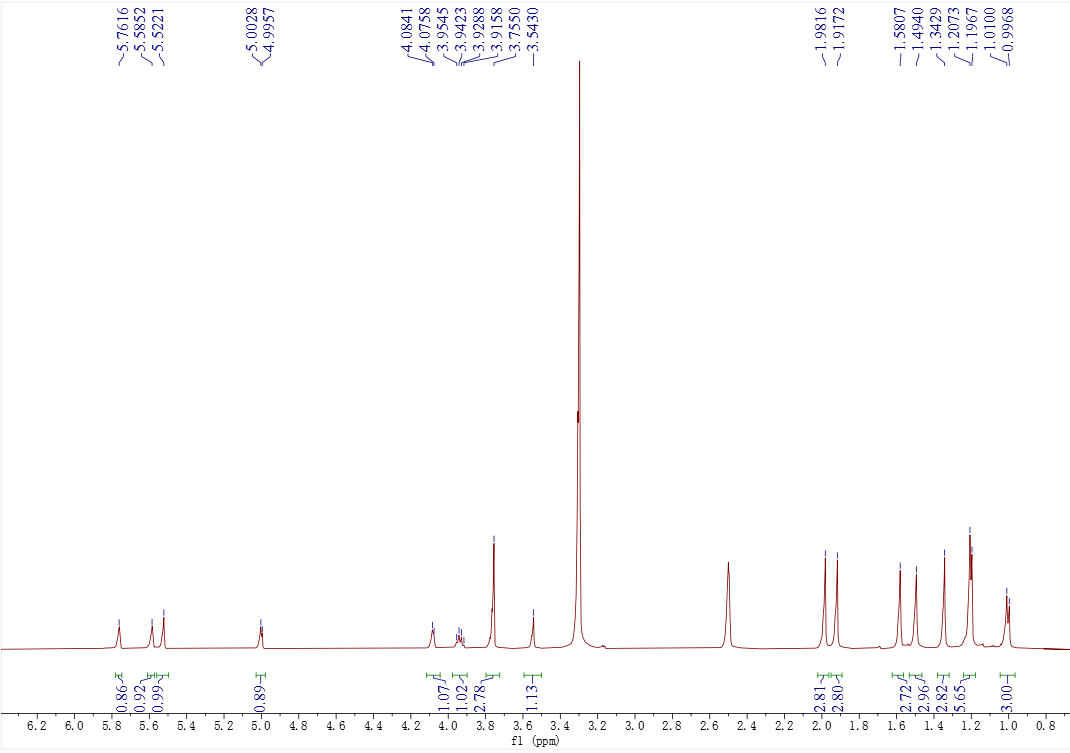


**Fig. S3.** ^13^C and DEPT spectra of compound **1a** (Recorded in DMSO-*d*_6_);


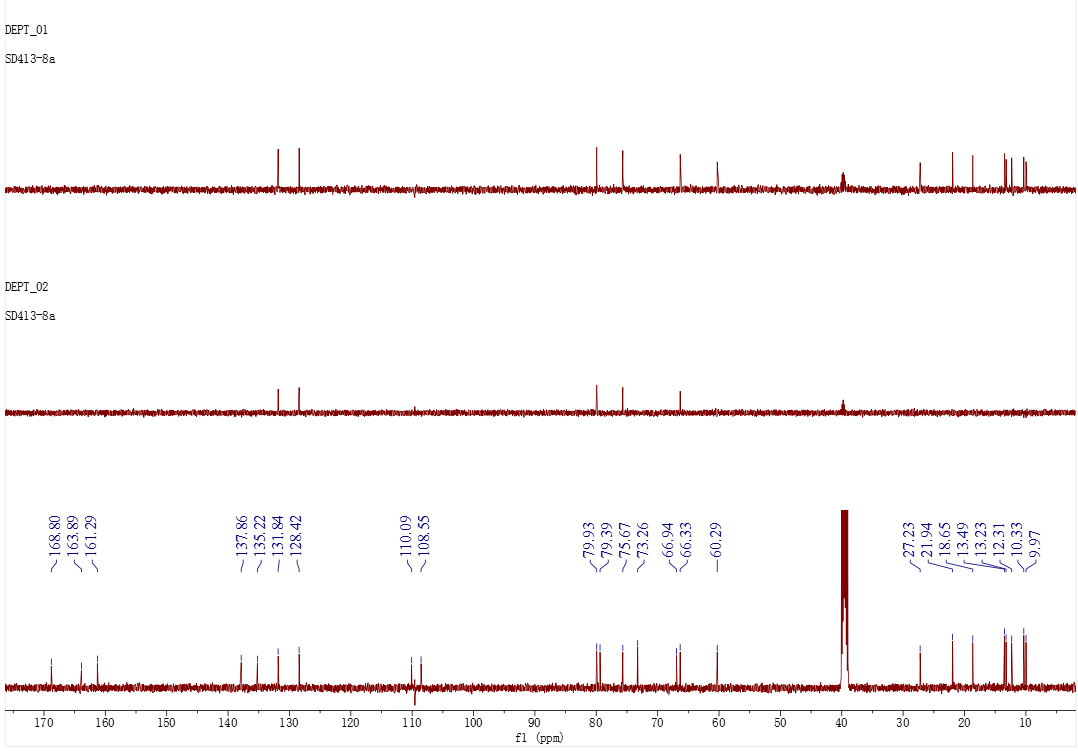


**Fig. S4.** ^1^H-^1^H COSY spectrum of compound **1a** (Recorded in DMSO-*d*_6_);


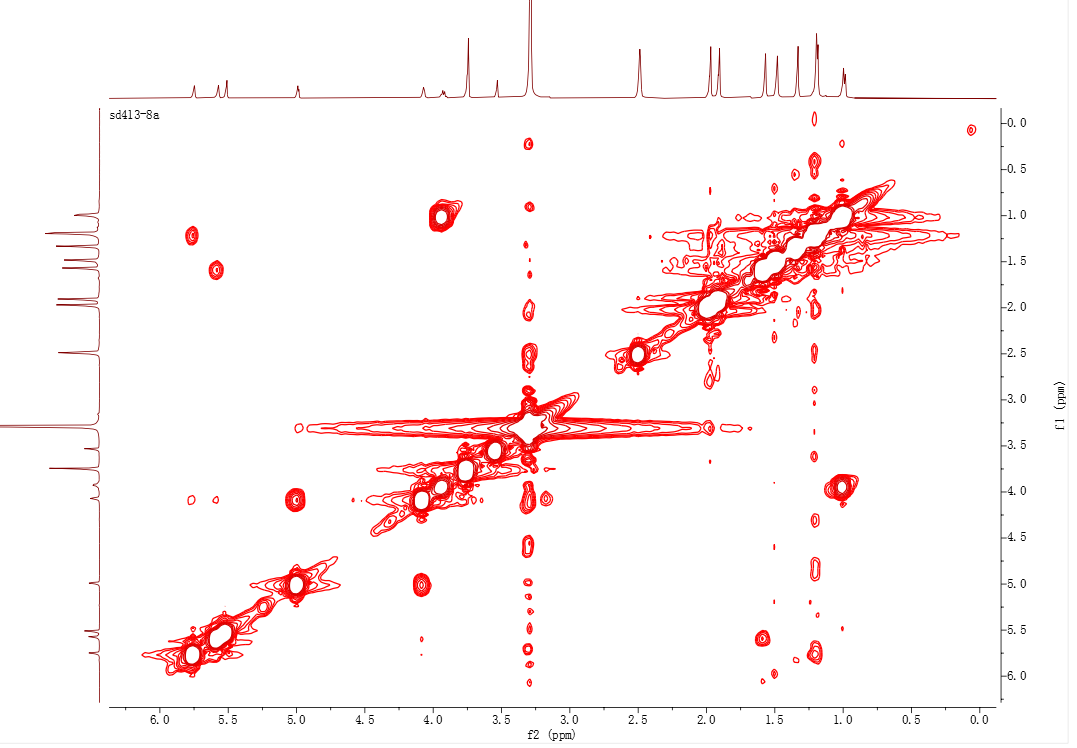


**Fig. S5.** NOESY spectrum of compound **1a** (Recorded in DMSO-*d*_6_);


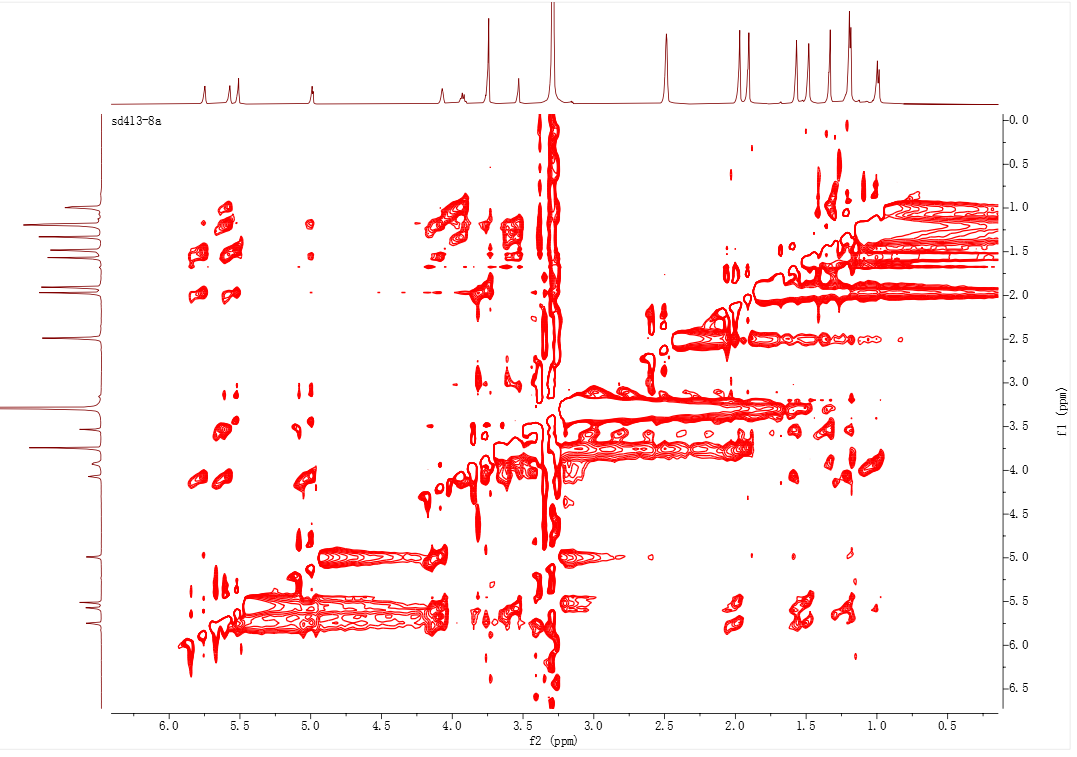


**Fig. S6.** ^1^H NMR spectrum of the (*S)*-MTPA ester compound **1a** (Recorded in DMSO-*d*_6_);

**Fig. S7.** ^1^H NMR spectrum of the (*R)*-MTPA ester compound **1a** (Recorded in DMSO-*d*_6_);

**Fig. S8.** Low-energy (≥ 1%) ωB97X/TZVP PCM/MeCN conformers of (6*S*,9*R*,12*S*,13*S*,14*R*,15*R*)-**1a**.


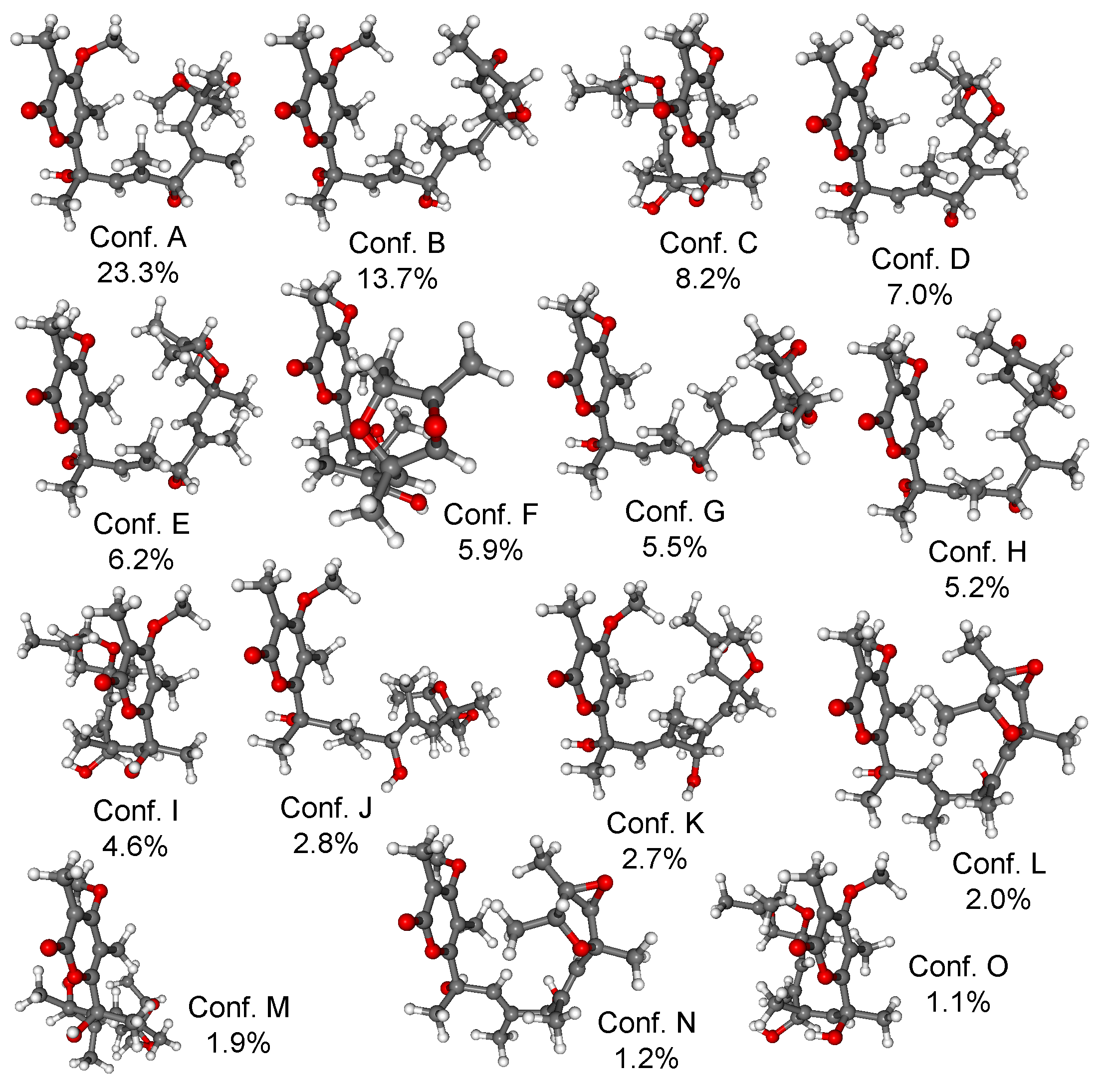


**Fig. S9.** Low-energy (≥ 1%) ωB97X/TZVP PCM/MeOH conformers of (6*S*,9*R*,12*S*,13*S*,14*R*,15*R*)-**1a**.


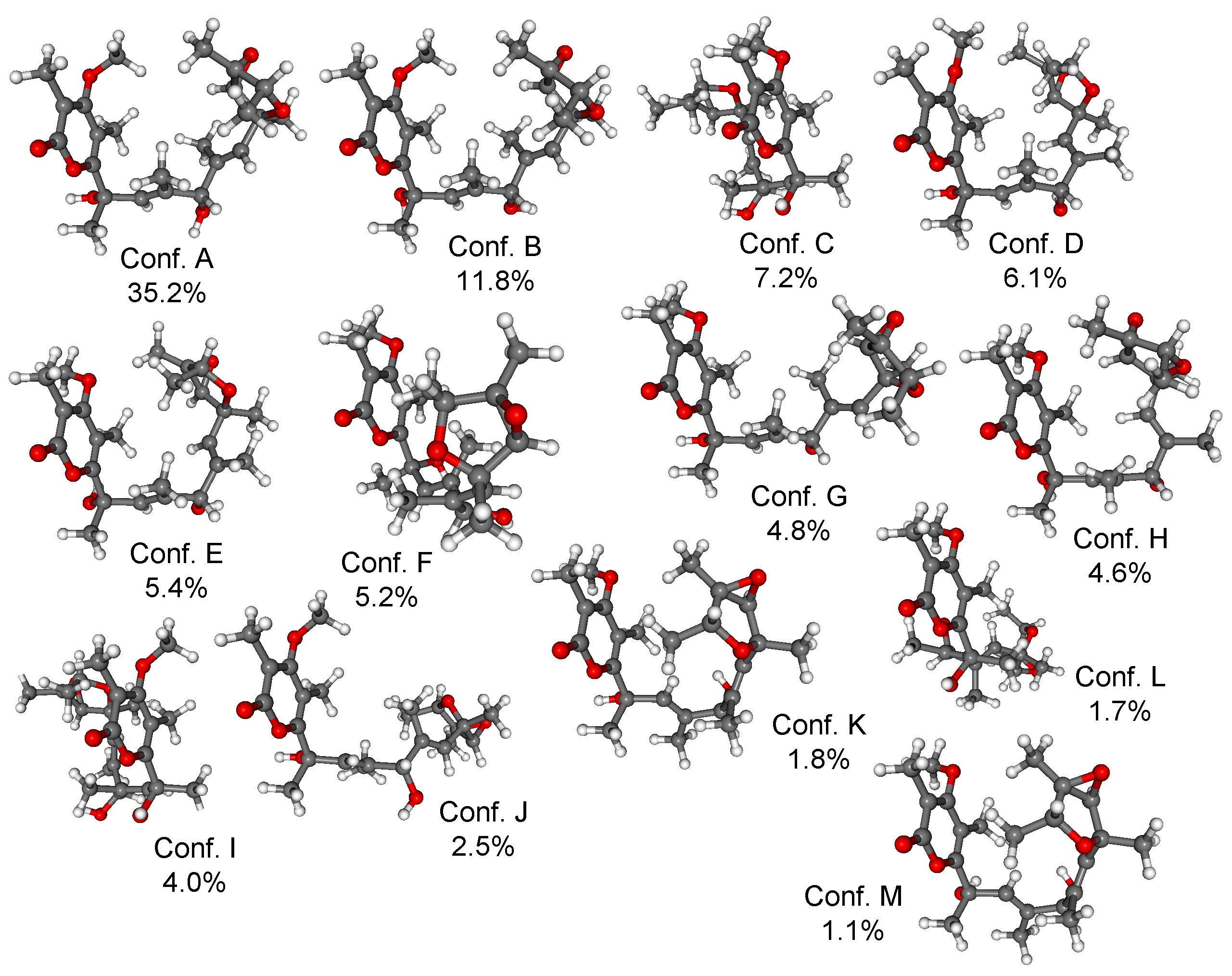


**Fig. S10.** Experimental ECD spectra of **1a**–**3b** measured in MeOH.


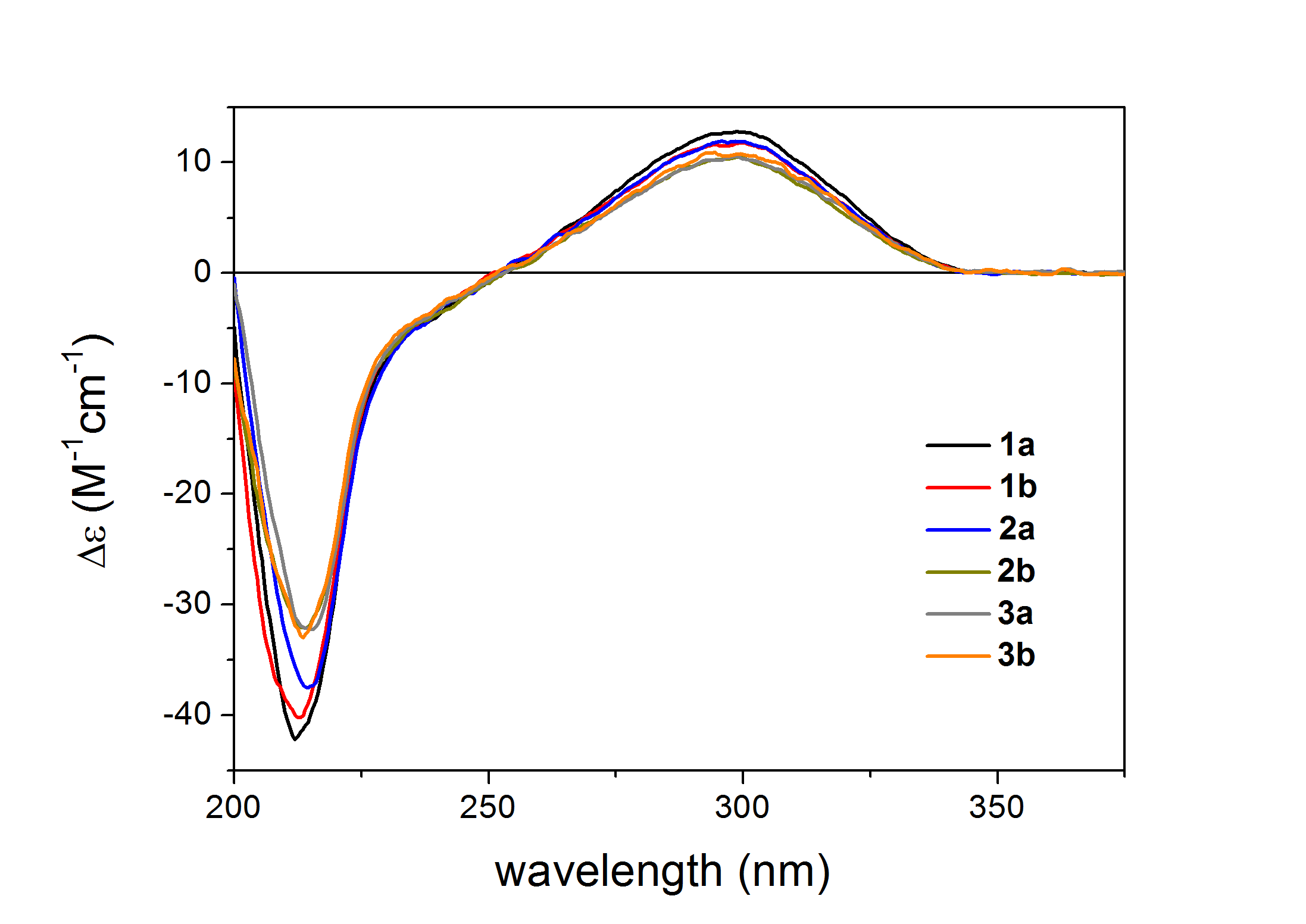


**Fig. S11.** Experimental ECD spectra of **1a**–**2b** measured in MeCN.


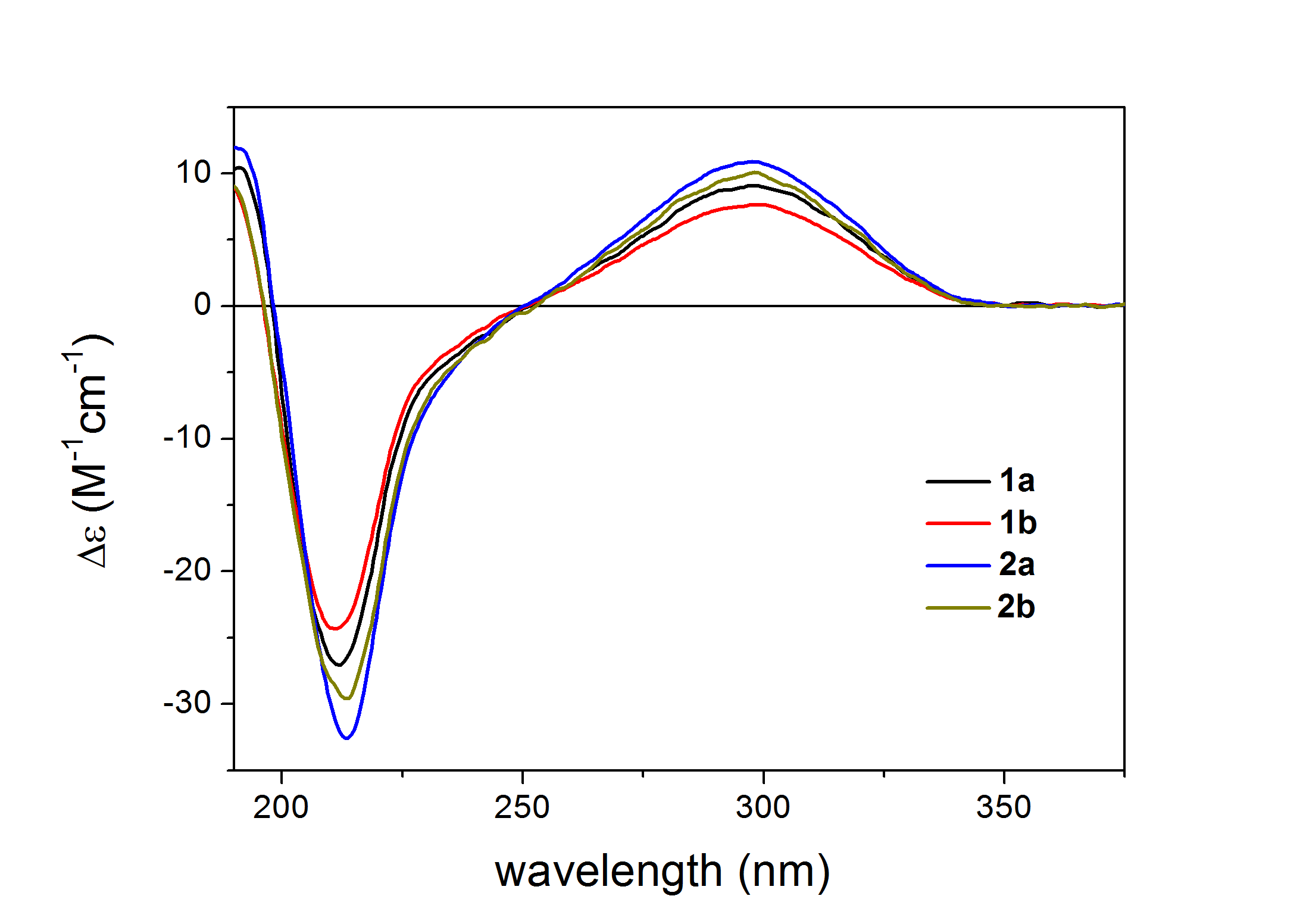


**Table S1.** Boltzmann populations and specific optical rotations of the low-energy conformers of (6*S*,9*R*,12*S*,13*S*,14*R*,15*R*)-**1a** computed at various levels for the ωB97X/TZVP PCM/MeOH optimized MMFF conformers.

| Conformer | Boltzmann population | B3LYP/TZVP | BH&HLYP/TZVP | CAM-B3LYP/TZVP | PBE0/TZVP |
| --- | --- | --- | --- | --- | --- |
| Conf. A | 35.18% | -53.82 | -48.77 | -58.09 | -50.78 |
| Conf. B | 11.80% | -32.01 | -28.44 | -37.97 | -29.69 |
| Conf. C | 7.19% | 62.74 | 17.80 | 34.77 | 60.72 |
| Conf. D | 6.09% | -299.89 | -255.09 | -271.86 | -296.14 |
| Conf. E | 5.41% | -214.23 | -207.66 | -201.08 | -214.87 |
| Conf. F | 5.24% | -158.07 | -164.21 | -145.63 | -159.55 |
| Conf. G | 4.80% | -75.61 | -106.42 | -94.72 | -75.57 |
| Conf. H | 4.60% | -121.77 | -103.34 | -104.96 | -122.44 |
| Conf. I | 3.96% | 88.73 | 84.32 | 75.25 | 90.74 |
| Conf. J | 2.47% | -470.23 | -364.78 | -373.03 | -460.70 |
| Conf. K | 1.76% | -150.98 | -134.95 | -137.35 | -151.55 |
| Conf. L | 1.69% | 136.59 | 120.59 | 117.84 | 134.72 |
| Conf. M | 1.05% | -151.87 | -133.75 | -137.09 | -151.05 |
| Average | N/A | -82.87 | -78.78 | -82.15 | -81.15 |

**Table S2.** Cartesian coordinates and energies of the low-energy conformers calculated at the ωB97X/TZVP PCM/MeCN level of (6*S*,9*R*,12*S*,13*S*,14*R*,15*R*)-**1a**.

(6*S*,9*R*,12*S*,13*S*,14*R*,15*R*)-**1a**, Conf A

C -3.615200 -1.926186 -0.444517

C -4.326096 -0.704439 -0.735197

O -3.847704 0.442086 -0.157031

C -2.762934 0.490726 0.658221

C -2.078993 -0.625747 0.970504

C -2.532146 -1.858767 0.368181

C -2.456053 1.913520 1.137072

C -1.086641 2.383245 0.682825

C -0.546093 2.268209 -0.523938

C 0.830679 2.833114 -0.821499

C 1.828257 1.769570 -1.279863

C 1.822226 0.580314 -0.683347

C 2.729307 -0.613666 -0.912107

O 2.436869 -1.625488 0.075218

C 3.360515 -1.582321 1.172365

C 4.575733 -0.855117 0.632122

C 4.185649 -0.276748 -0.643253

O -1.833569 -2.986143 0.683594

C -0.842198 -3.358182 -0.282206

C -4.131655 -3.186864 -1.063130

C -0.883363 -0.670991 1.876571

O -5.305050 -0.598318 -1.442733

C -3.522635 2.916338 0.696909

O -2.387537 1.912414 2.568705

C -1.196122 1.598922 -1.707462

C 2.774575 2.237371 -2.348025

C 2.491358 -1.251682 -2.273352

O 5.018134 -1.435083 -0.603874

C 5.636660 -0.321735 1.543980

C 2.764696 -0.903186 2.393354

O 1.393129 3.594892 0.240076

H -0.541599 2.911817 1.458627

H 0.706344 3.551865 -1.638462

H 3.610934 -2.621063 1.408358

H 4.636145 0.623125 -1.045021

H -0.404378 -4.288909 0.070832

H -0.060907 -2.594908 -0.353170

H -1.298383 -3.515113 -1.262847

H -3.643843 -4.057850 -0.629085

H -3.962990 -3.191821 -2.142835

H -5.208060 -3.270181 -0.905331

H -0.034810 -1.133231 1.368586

H -1.101684 -1.277595 2.758209

H -0.594105 0.321353 2.209754

H -3.584858 2.994064 -0.387616

H -4.506539 2.634021 1.077271

H -3.252401 3.890969 1.103860

H -3.259458 1.694657 2.913099

H -2.276671 1.738643 -1.723667

H -0.795618 2.000952 -2.640807

H -0.995466 0.523599 -1.708311

H 2.212034 2.507029 -3.246572

H 3.297943 3.138961 -2.017682

H 3.516877 1.494908 -2.632343

H 3.164662 -2.099186 -2.406126

H 1.459892 -1.603866 -2.336740

H 2.655280 -0.531813 -3.076286

H 6.433264 0.135694 0.957470

H 5.226592 0.429829 2.219811

H 6.064522 -1.126878 2.145593

H 3.457784 -0.955131 3.235377

H 2.540105 0.148007 2.192476

H 1.841828 -1.405966 2.683328

H 1.735629 2.979027 0.895880

H 1.050605 0.385916 0.057504

ωB97X Energy = -1461.66487097 a.u.

(6*S*,9*R*,12*S*,13*S*,14*R*,15*R*)-**1a**, Conf B

C -3.214179 -2.238340 -0.109036

C -3.624056 -1.432119 -1.233256

O -3.473090 -0.075945 -1.108556

C -2.973392 0.538022 -0.006805

C -2.594337 -0.169607 1.072806

C -2.707230 -1.607714 0.978821

C -2.911072 2.055018 -0.168602

C -1.488574 2.591444 -0.065660

C -0.389759 2.115113 -0.639056

C 0.951552 2.796101 -0.442378

C 1.894589 1.885616 0.331711

C 2.952084 1.379384 -0.297757

C 3.986187 0.392422 0.214094

O 4.738545 -0.120623 -0.903173

C 4.181257 -1.349695 -1.386273

C 3.420057 -1.918353 -0.203883

C 3.323977 -0.852535 0.779478

O -2.299167 -2.320213 2.067691

C -0.997616 -2.900365 1.934301

C -3.384409 -3.720563 -0.227989

C -2.078370 0.431787 2.348020

O -4.085930 -1.841316 -2.276856

C -3.539025 2.525787 -1.480237

O -3.688878 2.560587 0.924902

C -0.317344 0.874504 -1.489430

C 1.489529 1.610414 1.753538

C 4.987346 1.041845 1.156725

O 4.238660 -1.930383 0.974949

C 2.441534 -3.040065 -0.365487

C 3.300481 -1.137888 -2.606222

O 0.772155 4.043694 0.212805

H -1.405247 3.500959 0.522566

H 1.382352 2.964915 -1.437562

H 5.028099 -1.992609 -1.644243

H 2.515169 -0.781248 1.496519

H -0.245151 -2.122973 1.765292

H -0.970113 -3.616491 1.109391

H -0.789327 -3.411323 2.871056

H -4.371320 -3.951524 -0.631061

H -3.278730 -4.202115 0.742730

H -2.648429 -4.148598 -0.913435

H -1.193570 -0.102089 2.696344

H -2.832943 0.350236 3.133357

H -1.831353 1.481979 2.223974

H -2.994582 2.149093 -2.345563

H -4.578581 2.204566 -1.547711

H -3.501106 3.616937 -1.505633

H -3.718396 3.519682 0.847505

H -0.025989 0.005112 -0.889764

H -1.263091 0.644023 -1.976386

H 0.437072 0.992462 -2.270662

H 2.321620 1.281115 2.373488

H 1.068476 2.512187 2.201087

H 0.710517 0.843375 1.791083

H 5.702188 0.296375 1.507162

H 5.527275 1.831328 0.631517

H 4.483689 1.486814 2.015953

H 2.029238 -3.314616 0.606028

H 1.620326 -2.743676 -1.020685

H 2.926209 -3.917672 -0.798940

H 3.853434 -0.584930 -3.366294

H 2.999073 -2.096618 -3.033037

H 2.399261 -0.573714 -2.352618

H 1.637854 4.450550 0.306850

H 3.132326 1.680786 -1.326941

ωB97X Energy = -1461.66436748 a.u.

(6*S*,9*R*,12*S*,13*S*,14*R*,15*R*)-**1a**, Conf C

C 3.259127 -1.985874 -0.242353

C 3.720853 -0.971556 -1.155600

O 3.363929 0.324389 -0.866014

C 2.587843 0.693030 0.184094

C 2.115766 -0.224726 1.049417

C 2.484790 -1.602522 0.803928

C 2.473575 2.227504 0.258578

C 1.043799 2.693251 0.463262

C 0.048914 2.576126 -0.409643

C -1.321171 3.103709 -0.036327

C -2.337575 2.025805 0.330057

C -1.960291 0.758578 0.479520

C -2.781814 -0.450327 0.886823

O -1.982893 -1.638499 0.703964

C -2.231625 -2.248067 -0.569431

C -3.615543 -1.766715 -0.961815

C -3.949004 -0.676096 -0.058758

O 2.073309 -2.509730 1.731016

C 0.950159 -3.317334 1.353478

C 3.701861 -3.392894 -0.498073

C 1.286741 0.070459 2.267239

O 4.402084 -1.144208 -2.141889

C 3.364819 2.712786 1.405826

O 2.917754 2.815487 -0.958403

C 0.171123 1.960342 -1.776663

C -3.728917 2.561316 0.538816

C -3.169540 -0.417674 2.357779

O -4.552500 -1.958400 0.107749

C -4.136508 -1.939425 -2.354702

C -1.170953 -1.882593 -1.595305

O -1.833280 3.961956 -1.059004

H 0.848509 3.188425 1.411029

H -1.217462 3.758821 0.833654

H -2.232234 -3.329247 -0.400130

H -4.614434 0.135332 -0.329518

H 1.146194 -3.842898 0.415341

H 0.051430 -2.702444 1.255352

H 0.815044 -4.042618 2.152472

H 4.762638 -3.410851 -0.751435

H 3.161203 -3.831908 -1.340496

H 3.540615 -4.014611 0.381087

H 0.347276 -0.486593 2.232380

H 1.043654 1.125137 2.355909

H 1.816673 -0.241971 3.168599

H 3.045647 2.297793 2.363083

H 4.400353 2.414745 1.228946

H 3.316027 3.801585 1.458691

H 3.732112 2.379887 -1.231558

H -0.764815 1.481552 -2.074399

H 0.401782 2.729167 -2.517454

H 0.964266 1.215570 -1.826494

H -3.689695 3.484629 1.122100

H -4.200203 2.812444 -0.414003

H -4.376215 1.860891 1.062623

H -3.766761 -1.296826 2.602568

H -2.266750 -0.421085 2.971490

H -3.738772 0.481153 2.596312

H -5.148865 -1.541768 -2.424733

H -3.505910 -1.412871 -3.072396

H -4.157041 -2.997158 -2.626873

H -0.178623 -2.103271 -1.197525

H -1.306976 -2.459600 -2.511979

H -1.211447 -0.819882 -1.845803

H -2.047598 3.429493 -1.831446

H -0.915420 0.508411 0.324388

ωB97X Energy = -1461.66388152 a.u.

(6*S*,9*R*,12*S*,13*S*,14*R*,15*R*)-**1a**, Conf D

C 3.572837 1.655073 -0.443547

C 4.209277 0.398813 -0.752180

O 3.645303 -0.737599 -0.233231

C 2.550531 -0.742319 0.566294

C 1.910491 0.398592 0.874306

C 2.424928 1.624539 0.294275

C 2.189223 -2.154544 1.038753

C 0.788612 -2.582088 0.647805

C 0.153655 -2.395362 -0.502083

C -1.226842 -2.987670 -0.731136

C -2.301601 -1.913519 -0.920325

C -2.526975 -1.052047 0.067299

C -3.638152 -0.020017 0.151348

O -3.734931 0.801790 -1.027865

C -2.993113 2.017500 -0.875788

C -2.892613 2.229414 0.623851

C -3.303768 0.979170 1.243957

O 1.677832 2.703329 0.597307

C 1.518833 3.763951 -0.352561

C 4.300227 2.891688 -0.896338

C 0.687117 0.462212 1.741950

O 5.207105 0.244769 -1.427038

C 3.184393 -3.199768 0.532771

O 2.191854 -2.169685 2.473412

C 0.676279 -1.625778 -1.686408

C -3.062385 -1.989527 -2.210693

C -4.992926 -0.677879 0.371579

O -4.178740 2.103432 1.247346

C -1.951172 3.232960 1.213881

C -1.634108 1.944541 -1.551697

O -1.626043 -3.924752 0.260898

H 0.311954 -3.186276 1.413689

H -1.168487 -3.568351 -1.657252

H -3.595537 2.806662 -1.335628

H -2.950734 0.637062 2.212135

H 2.316366 4.499384 -0.272725

H 0.570851 4.239155 -0.108035

H 1.476666 3.362457 -1.366345

H 5.336562 2.629737 -1.101802

H 4.292928 3.657249 -0.120957

H 3.885518 3.322552 -1.809667

H -0.193370 0.713152 1.145127

H 0.796812 1.240577 2.498352

H 0.511483 -0.485646 2.241913

H 3.193709 -3.249067 -0.555724

H 4.197024 -2.980515 0.877115

H 2.878556 -4.169858 0.925383

H 3.091757 -2.009407 2.774629

H 1.764149 -1.620045 -1.741483

H 0.304320 -2.062594 -2.616367

H 0.335184 -0.586474 -1.659563

H -2.372497 -1.879330 -3.053395

H -3.528060 -2.974646 -2.311386

H -3.823968 -1.218591 -2.293927

H -5.759521 0.087000 0.501769

H -5.254756 -1.303685 -0.482311

H -4.963144 -1.310109 1.261314

H -2.094245 3.282224 2.293353

H -0.913733 2.956439 1.017587

H -2.132431 4.224654 0.792661

H -1.756334 1.638361 -2.591289

H -1.146247 2.921563 -1.539002

H -0.981978 1.221627 -1.054128

H -2.061775 -3.445644 0.971692

H -1.882560 -1.107525 0.945758

ωB97X Energy = -1461.66372941 a.u.

(6*S*,9*R*,12*S*,13*S*,14*R*,15*R*)-**1a**, Conf E

C -3.141376 1.702310 0.827671

C -3.806825 0.478386 1.204502

O -3.441581 -0.658242 0.527762

C -2.491042 -0.697111 -0.437248

C -1.849748 0.421118 -0.827931

C -2.216998 1.650942 -0.161868

C -2.293355 -2.102422 -1.016192

C -0.864976 -2.595411 -0.834878

C -0.132713 -2.586545 0.273189

C 1.294807 -3.100022 0.253729

C 2.283956 -2.005222 0.670888

C 2.618883 -1.067972 -0.210307

C 3.620480 0.056738 -0.021812

O 3.479978 0.744838 1.236133

C 2.645342 1.900283 1.101982

C 2.757541 2.292621 -0.360514

C 3.351031 1.150432 -1.041551

O -1.549126 2.782316 -0.528817

C -2.166702 3.516425 -1.592033

C -3.526536 2.946641 1.563439

C -0.798080 0.453585 -1.902540

O -4.659413 0.361618 2.057151

C -3.279270 -3.111559 -0.438073

O -2.610905 -2.060023 -2.413493

C -0.582356 -2.077706 1.615188

C 2.813240 -2.123789 2.071554

C 5.048865 -0.463987 -0.105872

O 4.125841 2.320583 -0.790961

C 1.855990 3.324903 -0.963281

C 1.209502 1.616209 1.515561

O 1.590626 -3.632186 -1.028210

H -0.431257 -3.020939 -1.734137

H 1.363848 -3.903419 1.001039

H 3.077800 2.669867 1.748128

H 3.171158 0.902239 -2.082943

H -3.141760 3.896677 -1.277321

H -2.286946 2.891567 -2.480581

H -1.502798 4.347652 -1.818574

H -4.574845 3.194468 1.382505

H -2.906366 3.785809 1.254479

H -3.410887 2.801299 2.639142

H -1.223260 0.738334 -2.869024

H -0.298703 -0.508318 -2.009199

H -0.032497 1.189596 -1.658669

H -3.138536 -3.239487 0.633671

H -4.306356 -2.797876 -0.625751

H -3.103566 -4.068373 -0.929775

H -1.945759 -1.545338 -2.879849

H -0.097913 -1.125001 1.850210

H -1.658131 -1.930709 1.675741

H -0.302434 -2.784557 2.400392

H 1.994143 -2.121896 2.796357

H 3.332569 -3.079875 2.189279

H 3.489702 -1.313058 2.327513

H 5.752815 0.366695 -0.042677

H 5.243864 -1.161257 0.710109

H 5.201483 -0.991765 -1.049484

H 2.044644 3.407916 -2.033999

H 0.804786 3.078177 -0.805412

H 2.045920 4.297431 -0.502322

H 1.195567 1.186782 2.518495

H 0.617449 2.533803 1.524797

H 0.737543 0.906368 0.829905

H 2.533444 -3.815247 -1.057552

H 2.173953 -1.131886 -1.202649

ωB97X Energy = -1461.66361424 a.u.

(6*S*,9*R*,12*S*,13*S*,14*R*,15*R*)-**1a**, Conf F

C 2.735325 -2.045288 0.575762

C 1.855093 -1.152863 1.286381

O 1.970959 0.180908 1.004116

C 2.860935 0.704851 0.122589

C 3.775302 -0.075732 -0.485293

C 3.671781 -1.499355 -0.239828

C 2.687819 2.221423 0.041242

C 1.244247 2.648771 0.265230

C 0.183661 2.211666 -0.407440

C -1.192978 2.757835 -0.065764

C -2.071720 1.682032 0.545552

C -3.171521 1.266150 -0.073723

C -4.176537 0.242156 0.419517

O -3.585960 -0.875509 1.107147

C -3.347482 -1.969806 0.216046

C -4.326045 -1.759834 -0.925743

C -4.839892 -0.406708 -0.782800

O 4.540424 -2.289156 -0.930414

C 5.597631 -2.856989 -0.149843

C 2.539286 -3.515092 0.782624

C 4.877331 0.404226 -1.389266

O 1.013366 -1.474325 2.099640

C 3.586277 2.866687 1.098635

O 3.107753 2.628524 -1.265898

C 0.229011 1.151627 -1.469012

C -1.564082 1.173517 1.866908

C -5.183860 0.879789 1.366668

O -5.652927 -1.524666 -0.431118

C -4.210961 -2.525420 -2.206895

C -1.903184 -2.021836 -0.253812

O -1.819627 3.311766 -1.215921

H 1.101044 3.398625 1.039509

H -1.067995 3.541790 0.692607

H -3.593847 -2.879646 0.772099

H -5.186767 0.200040 -1.613175

H 6.191357 -3.461927 -0.830732

H 5.205492 -3.485715 0.651840

H 6.221464 -2.067903 0.278334

H 2.943787 -3.842528 1.743880

H 3.022118 -4.084952 -0.010078

H 1.473787 -3.749269 0.785389

H 4.644075 0.193605 -2.435221

H 5.802577 -0.121800 -1.152703

H 5.040321 1.471649 -1.289601

H 3.305735 2.529210 2.098614

H 4.629718 2.607171 0.916215

H 3.479517 3.952947 1.057408

H 2.754524 3.510928 -1.412773

H 0.038341 0.171919 -1.019817

H -0.545416 1.324432 -2.217718

H 1.198029 1.117108 -1.964513

H -2.336587 0.650513 2.424076

H -0.732769 0.476158 1.722811

H -1.185368 2.003246 2.470254

H -5.949713 0.153336 1.641689

H -4.678821 1.226138 2.269869

H -5.659257 1.739775 0.890600

H -5.004555 -2.226605 -2.891701

H -3.249055 -2.337886 -2.685975

H -4.297125 -3.598367 -2.020252

H -1.229527 -1.978104 0.604795

H -1.709870 -2.949407 -0.797242

H -1.679083 -1.179221 -0.911940

H -1.246300 4.004801 -1.554519

H -3.424336 1.738769 -1.018842

ωB97X Energy = -1461.66357270 a.u.

(6*S*,9*R*,12*S*,13*S*,14*R*,15*R*)-**1a**, Conf G

C 3.522800 -2.082285 0.251259

C 3.568084 -1.391191 1.517229

O 3.290572 -0.049041 1.499042

C 2.964781 0.648816 0.380530

C 2.923695 0.049377 -0.823869

C 3.231870 -1.362606 -0.860497

C 2.701664 2.129140 0.674470

C 1.298533 2.556972 0.285594

C 0.146118 1.967305 0.579347

C -1.177349 2.574911 0.130129

C -2.018155 1.560471 -0.633179

C -3.188531 1.189787 -0.121225

C -4.197503 0.188992 -0.656138

O -5.228091 -0.013969 0.330576

C -4.947066 -1.151272 1.155674

C -4.019668 -2.014040 0.321641

C -3.576486 -1.191554 -0.792664

O 3.166578 -1.963063 -2.082187

C 4.437398 -2.197245 -2.698705

C 3.794811 -3.554026 0.261004

C 2.577587 0.740756 -2.110895

O 3.828557 -1.885078 2.593283

C 2.969506 2.486669 2.136586

O 3.563214 2.917045 -0.157899

C -0.006507 0.691064 1.361688

C -1.408814 1.061608 -1.913870

C -4.896331 0.682369 -1.914149

O -4.547685 -2.216242 -0.997838

C -3.256641 -3.151350 0.926050

C -4.335913 -0.751049 2.488278

O -1.034422 3.784307 -0.599959

H 1.291450 3.499505 -0.252438

H -1.728974 2.858486 1.030875

H -5.902510 -1.658468 1.319646

H -2.621732 -1.330288 -1.286635

H 4.960437 -1.252522 -2.868877

H 4.233825 -2.678515 -3.652055

H 5.057749 -2.851001 -2.081568

H 3.479620 -4.012789 -0.674818

H 3.258771 -4.027639 1.084413

H 4.858267 -3.757999 0.409846

H 3.411879 0.682365 -2.813461

H 2.345287 1.789230 -1.952472

H 1.724267 0.252437 -2.584932

H 2.311399 1.943597 2.813390

H 4.003009 2.265559 2.410863

H 2.791680 3.555417 2.257546

H 4.473850 2.745140 0.101732

H -0.839717 0.777626 2.064148

H -0.238674 -0.148163 0.698404

H 0.883179 0.433162 1.932424

H -1.137599 1.894944 -2.568513

H -0.487940 0.506498 -1.710250

H -2.073760 0.417573 -2.484007

H -5.597218 -0.074944 -2.267359

H -5.446562 1.597567 -1.690055

H -4.177751 0.903089 -2.703949

H -2.675128 -3.658004 0.155936

H -2.573971 -2.793701 1.698252

H -3.939637 -3.873397 1.379248

H -4.972651 -0.012313 2.976203

H -4.250348 -1.617071 3.147636

H -3.341291 -0.317643 2.354498

H -0.529387 3.611245 -1.400470

H -3.506900 1.649616 0.810918

ωB97X Energy = -1461.66351127 a.u.

(6*S*,9*R*,12*S*,13*S*,14*R*,15*R*)-**1a**, Conf H

C 2.587366 -1.873692 0.538320

C 3.047644 -0.864172 1.459813

O 2.912740 0.442542 1.062594

C 2.369524 0.826675 -0.118228

C 1.943253 -0.078739 -1.020374

C 2.066647 -1.472240 -0.647677

C 2.297311 2.348548 -0.257577

C 0.841803 2.803302 -0.325061

C -0.109252 2.647167 0.588494

C -1.537043 3.076956 0.310518

C -2.484704 1.892114 0.116755

C -2.032414 0.754411 -0.403253

C -2.822077 -0.493474 -0.755462

O -3.759218 -0.883723 0.264486

C -3.172785 -1.824794 1.174144

C -2.040504 -2.467795 0.393625

C -1.854848 -1.662670 -0.802439

O 1.572139 -2.403219 -1.513345

C 2.490272 -2.814616 -2.532637

C 2.720659 -3.300682 0.967344

C 1.348487 0.281928 -2.353931

O 3.539969 -1.060007 2.549500

C 3.058432 3.074561 0.849479

O 2.935595 2.623529 -1.509416

C 0.069031 2.046840 1.957397

C -3.908652 2.180346 0.493901

C -3.589900 -0.319012 -2.057039

O -2.503804 -2.928081 -0.885263

C -0.976431 -3.280867 1.062653

C -2.703773 -1.161422 2.457189

O -1.675406 3.977440 -0.783625

H 0.585744 3.303053 -1.255118

H -1.882049 3.643239 1.182852

H -3.950245 -2.561641 1.397268

H -0.906109 -1.578519 -1.324385

H 3.362602 -3.296684 -2.084153

H 2.812433 -1.963264 -3.136972

H 1.957058 -3.525632 -3.158672

H 2.308446 -3.971942 0.216524

H 2.196933 -3.465185 1.911648

H 3.769711 -3.554794 1.133514

H 2.120556 0.335253 -3.123901

H 0.855302 1.249809 -2.323859

H 0.624391 -0.472931 -2.660705

H 2.625245 2.894581 1.831346

H 4.103157 2.763064 0.861451

H 3.010036 4.146868 0.648810

H 2.942379 3.578200 -1.632358

H -0.832675 1.514207 2.264493

H 0.906282 1.354763 2.021996

H 0.235854 2.842280 2.689780

H -3.984832 2.315945 1.577563

H -4.233301 3.116026 0.030859

H -4.587815 1.381467 0.207824

H -4.074621 -1.256958 -2.331395

H -4.349207 0.456809 -1.949059

H -2.908639 -0.021376 -2.857027

H -0.266812 -3.641072 0.316687

H -0.431669 -2.678032 1.792016

H -1.411202 -4.138468 1.581024

H -2.383977 -1.911337 3.183499

H -1.869143 -0.483051 2.266365

H -3.522436 -0.588835 2.894865

H -1.675487 3.461123 -1.595686

H -0.970316 0.693136 -0.632264

ωB97X Energy = -1461.66345682 a.u.

(6*S*,9*R*,12*S*,13*S*,14*R*,15*R*)-**1a**, Conf I

C 3.160301 -1.802268 -0.527021

C 3.718834 -0.688111 -1.250567

O 3.331341 0.565711 -0.842318

C 2.480871 0.812240 0.185797

C 1.981028 -0.195872 0.925330

C 2.356825 -1.536455 0.533324

C 2.301047 2.327915 0.388034

C 0.842206 2.723439 0.534939

C -0.105763 2.585356 -0.386131

C -1.513037 3.057188 -0.073950

C -2.504356 1.942426 0.252473

C -2.081465 0.699344 0.468917

C -2.875118 -0.527391 0.882036

O -2.004479 -1.677964 0.871946

C -2.094276 -2.397640 -0.363962

C -3.439376 -2.004309 -0.944440

C -3.919564 -0.875248 -0.163746

O 1.802329 -2.546965 1.258420

C 2.690215 -3.193006 2.177760

C 3.505494 -3.177980 -1.005731

C 1.096727 -0.040039 2.130463

O 4.488210 -0.747175 -2.184058

C 3.099770 2.744181 1.626219

O 2.791214 3.036958 -0.744827

C 0.098939 2.003662 -1.756637

C -3.927661 2.419676 0.360090

C -3.418393 -0.422782 2.299792

O -4.485591 -2.171171 0.025245

C -3.788833 -2.291598 -2.371495

C -0.930186 -2.083653 -1.288530

O -2.008829 3.898344 -1.117943

H 0.583214 3.190200 1.482208

H -1.472924 3.714496 0.799841

H -2.087020 -3.461180 -0.106337

H -4.581733 -0.115379 -0.563086

H 3.535475 -3.647366 1.656826

H 2.108375 -3.965241 2.674673

H 3.058589 -2.477595 2.917501

H 2.817877 -3.914645 -0.592493

H 4.524865 -3.454856 -0.724721

H 3.449484 -3.217731 -2.094296

H 1.606728 -0.410368 3.022910

H 0.183416 -0.625389 2.009394

H 0.817625 0.995311 2.304663

H 2.743455 2.234527 2.522651

H 4.155849 2.502737 1.489066

H 3.000712 3.821301 1.769206

H 3.660096 2.689494 -0.973013

H 0.157972 2.805397 -2.497093

H 1.017040 1.425647 -1.834677

H -0.740243 1.360525 -2.035051

H -4.595095 1.671311 0.781547

H -3.976252 3.311212 0.991160

H -4.318379 2.707196 -0.618160

H -3.996002 -1.316617 2.538655

H -2.585595 -0.342953 3.000782

H -4.050709 0.456432 2.422095

H -4.804901 -1.956382 -2.578938

H -3.106615 -1.775288 -3.048292

H -3.726311 -3.363361 -2.573094

H 0.010239 -2.265315 -0.765248

H -0.956831 -2.723557 -2.172924

H -0.950494 -1.040571 -1.614962

H -2.179955 3.358816 -1.896209

H -1.020895 0.492825 0.375903

ωB97X Energy = -1461.66333087 a.u.

(6*S*,9*R*,12*S*,13*S*,14*R*,15*R*)-**1a**, Conf J

C -5.201038 -1.182810 -0.248503

C -5.378454 0.179903 -0.687671

O -4.405946 1.076857 -0.329512

C -3.300164 0.755741 0.388396

C -3.069091 -0.511366 0.782003

C -4.071445 -1.493522 0.434614

C -2.397821 1.975179 0.634881

C -1.212985 1.848183 -0.299413

C 0.098889 1.846849 -0.075316

C 1.018868 1.700346 -1.282044

C 1.892751 0.464619 -1.182948

C 3.217824 0.562824 -1.189529

C 4.228572 -0.568350 -1.152310

O 3.883167 -1.629124 -0.242323

C 4.481027 -1.425430 1.042507

C 5.682944 -0.537466 0.775952

C 5.529125 -0.033112 -0.578960

O -3.846549 -2.763313 0.874584

C -3.410277 -3.680014 -0.135087

C -6.290755 -2.156409 -0.572625

C -1.853601 -0.964361 1.539892

O -6.309367 0.606948 -1.336665

C -3.126243 3.285514 0.325107

O -1.975789 1.985821 1.991634

C 0.807166 1.972194 1.245365

C 1.111510 -0.817599 -1.098794

C 4.414416 -1.188126 -2.530092

O 6.478228 -1.060156 -0.299270

C 6.415103 0.142489 1.890595

C 3.507549 -0.812395 2.034670

O 1.828586 2.859963 -1.437314

H -1.540894 1.748127 -1.332917

H 0.392163 1.576283 -2.175191

H 4.802545 -2.410915 1.393534

H 5.931587 0.914650 -0.922183

H -3.265052 -4.636780 0.360358

H -2.465533 -3.345746 -0.572421

H -4.159096 -3.784124 -0.923060

H -7.263893 -1.704008 -0.378018

H -6.195515 -3.058716 0.029544

H -6.271415 -2.437487 -1.628781

H -2.118978 -1.239089 2.562884

H -1.099095 -0.185461 1.583836

H -1.423632 -1.849723 1.068542

H -3.400488 3.359633 -0.725995

H -4.035275 3.376464 0.923603

H -2.451878 4.105371 0.573893

H -2.751122 2.131886 2.542367

H 0.231560 2.542602 1.967030

H 0.992658 0.981463 1.673861

H 1.778811 2.446251 1.095645

H 0.652320 -0.924056 -0.111024

H 0.296925 -0.813889 -1.828746

H 1.737179 -1.689713 -1.265572

H 5.203793 -1.940343 -2.496572

H 3.486494 -1.658615 -2.858712

H 4.682785 -0.419038 -3.257104

H 7.256530 0.708734 1.491499

H 5.756284 0.827731 2.425713

H 6.795808 -0.592820 2.603286

H 2.594093 -1.408380 2.068969

H 3.941201 -0.792327 3.036695

H 3.244061 0.207608 1.746000

H 1.238546 3.613193 -1.527183

H 3.644644 1.557969 -1.277006

ωB97X Energy = -1461.66286700 a.u.

(6*S*,9*R*,12*S*,13*S*,14*R*,15*R*)-**1a**, Conf K

C -3.747147 -1.893234 -0.174554

C -4.451216 -0.731607 -0.658030

O -3.910022 0.489741 -0.353753

C -2.793990 0.665125 0.398987

C -2.109892 -0.388290 0.882022

C -2.599218 -1.701582 0.525177

C -2.476198 2.147127 0.610051

C -1.069488 2.541580 0.210808

C -0.306329 2.105378 -0.784698

C 1.080352 2.716654 -0.984467

C 2.132589 1.972056 -0.176895

C 2.913321 1.096247 -0.805549

C 4.018569 0.220872 -0.241263

O 4.369248 -0.782331 -1.216817

C 3.689755 -2.017474 -0.964313

C 3.354125 -1.978214 0.514081

C 3.560778 -0.606401 0.948040

O -1.860466 -2.750341 0.978234

C -1.209612 -3.526443 -0.033562

C -4.363165 -3.229689 -0.452730

C -0.865291 -0.276463 1.714634

O -5.473495 -0.732536 -1.310356

C -3.462129 3.054291 -0.127224

O -2.540401 2.425982 2.016963

C -0.646660 1.011520 -1.760069

C 2.172960 2.297686 1.291269

C 5.282083 1.024815 0.030940

O 4.501076 -1.622987 1.297872

C 2.393386 -2.947506 1.128931

C 2.455242 -2.175310 -1.836498

O 1.143138 4.088024 -0.615671

H -0.690743 3.349499 0.831442

H 1.334711 2.615480 -2.045460

H 4.406589 -2.815498 -1.179125

H 3.016187 -0.160158 1.770884

H -1.927044 -4.024722 -0.686377

H -0.616461 -4.272935 0.489662

H -0.555774 -2.888847 -0.636210

H -4.160226 -3.566958 -1.472513

H -5.445967 -3.160527 -0.346249

H -3.990579 -3.980193 0.243416

H -0.886757 -1.009629 2.520530

H -0.770537 0.715697 2.147309

H 0.025907 -0.480811 1.111073

H -3.424336 2.884373 -1.203311

H -4.485531 2.886069 0.212885

H -3.185814 4.089389 0.075500

H -3.446796 2.290520 2.310989

H -0.197448 0.061011 -1.452940

H -1.718391 0.854066 -1.868208

H -0.246021 1.250395 -2.747453

H 2.053804 3.371600 1.434000

H 1.351726 1.807797 1.821922

H 3.108336 1.996579 1.758603

H 6.055927 0.371171 0.435278

H 5.642023 1.468646 -0.898596

H 5.085130 1.830444 0.739792

H 2.323612 -2.769681 2.202078

H 1.399433 -2.832304 0.695118

H 2.722065 -3.976177 0.964572

H 2.725091 -2.048706 -2.885348

H 2.018842 -3.168981 -1.712898

H 1.698399 -1.427892 -1.582388

H 0.431142 4.548398 -1.069195

H 2.799923 0.981640 -1.881088

ωB97X Energy = -1461.66284181 a.u.

(6*S*,9*R*,12*S*,13*S*,14*R*,15*R*)-**1a**, Conf L

C 3.237804 -1.279031 0.526774

C 2.942159 -0.498777 1.703850

O 2.340143 0.716734 1.505072

C 1.988737 1.198681 0.287795

C 2.238284 0.507201 -0.841083

C 2.904547 -0.769425 -0.685106

C 1.222732 2.524242 0.387612

C -0.237917 2.214220 0.107754

C -1.232218 3.046080 -0.184244

C -2.613467 2.467664 -0.487395

C -2.885488 1.126339 0.176694

C -2.976847 0.017635 -0.550477

C -3.276693 -1.399120 -0.101039

O -2.780601 -1.726669 1.209033

C -1.539380 -2.439684 1.155355

C -1.450465 -2.979889 -0.259150

C -2.530289 -2.359227 -1.011010

O 3.125863 -1.515545 -1.803152

C 4.279950 -1.131264 -2.558126

C 3.888003 -2.610098 0.734864

C 1.795287 0.955180 -2.207205

O 3.178834 -0.815896 2.849567

C 1.368071 3.153976 1.774126

O 1.710768 3.426434 -0.603968

C -1.140933 4.537340 -0.324232

C -3.036973 1.208142 1.671857

C -4.779683 -1.647091 -0.097540

O -2.634501 -3.718857 -0.595177

C -0.156443 -3.477584 -0.825789

C -0.359275 -1.560697 1.529459

O -2.812337 2.470848 -1.898653

H -0.488318 1.163160 0.201605

H -3.362223 3.169912 -0.105080

H -1.623697 -3.264489 1.869985

H -2.497989 -2.186195 -2.081942

H 5.185111 -1.272453 -1.961973

H 4.212466 -0.089894 -2.880825

H 4.308305 -1.780861 -3.429401

H 4.061189 -3.107575 -0.216968

H 3.255897 -3.249871 1.355666

H 4.840105 -2.496115 1.257106

H 1.587494 0.084349 -2.828769

H 2.563772 1.552004 -2.702367

H 0.899343 1.568844 -2.148900

H 0.943760 2.515313 2.547488

H 2.419173 3.331530 2.013075

H 0.836554 4.105491 1.772124

H 2.599549 3.695389 -0.350274

H -1.707956 5.015106 0.480666

H -0.114443 4.893446 -0.295511

H -1.595628 4.852890 -1.266504

H -2.059965 1.262271 2.161156

H -3.581457 2.116616 1.943195

H -3.559760 0.345722 2.076169

H -4.984281 -2.695699 0.122446

H -5.254135 -1.021989 0.660896

H -5.210125 -1.393489 -1.068360

H -0.311994 -3.845202 -1.839919

H 0.589310 -2.680093 -0.855613

H 0.240350 -4.292817 -0.216191

H -0.561326 -1.047650 2.470615

H 0.541235 -2.166741 1.656862

H -0.166073 -0.812603 0.756812

H -2.032481 2.086844 -2.311423

H -2.894123 0.119566 -1.630087

ωB97X Energy = -1461.66255452 a.u.

(6*S*,9*R*,12*S*,13*S*,14*R*,15*R*)-**1a**, Conf M

C 5.305832 0.877900 -0.269831

C 5.415501 -0.551703 -0.124952

O 4.243489 -1.264946 -0.188005

C 3.022362 -0.714730 -0.404535

C 2.874836 0.610746 -0.589013

C 4.078215 1.411892 -0.486571

C 1.958984 -1.817627 -0.489856

C 0.635494 -1.394724 0.116833

C 0.443680 -0.973879 1.361258

C -0.930469 -0.531496 1.842865

C -2.084479 -0.931408 0.940180

C -2.625119 -0.041166 0.113931

C -3.758990 -0.228067 -0.876881

O -4.124448 1.051180 -1.433412

C -5.225290 1.635677 -0.728136

C -5.928120 0.464851 -0.068422

C -5.031827 -0.674999 -0.178392

O 3.958566 2.758158 -0.660880

C 3.546857 3.464394 0.514255

C 6.569211 1.673720 -0.171547

C 1.569524 1.284118 -0.919852

O 6.433878 -1.181364 0.057588

C 1.776970 -2.187381 -1.964170

O 2.391681 -2.966480 0.235311

C 1.528892 -0.809047 2.386799

C -2.498229 -2.368030 1.107979

C -3.354568 -1.104061 -2.054163

O -6.184225 -0.583445 -1.015335

C -6.953348 0.671836 1.002756

C -4.766327 2.686214 0.269337

O -0.826295 0.875288 2.049993

H -0.214054 -1.449900 -0.557293

H -1.101540 -1.018990 2.814232

H -5.870751 2.094217 -1.483441

H -5.021973 -1.490444 0.535336

H 3.431769 4.506324 0.225954

H 4.308885 3.377937 1.293287

H 2.594793 3.081955 0.891176

H 7.030096 1.545684 0.810331

H 6.372249 2.731165 -0.334830

H 7.293605 1.332061 -0.913655

H 1.765322 2.199612 -1.476856

H 1.006776 1.541196 -0.019145

H 0.929151 0.647763 -1.528122

H 1.423811 -1.334136 -2.546333

H 2.724314 -2.526467 -2.388643

H 1.046174 -2.993987 -2.042943

H 3.290904 -3.176135 -0.040124

H 2.442777 -1.329431 2.108872

H 1.752456 0.252313 2.526677

H 1.196191 -1.196728 3.353147

H -1.621647 -3.020154 1.079458

H -2.971975 -2.512755 2.083468

H -3.191276 -2.709924 0.342402

H -4.197241 -1.219287 -2.736836

H -2.527619 -0.633233 -2.588851

H -3.025705 -2.088783 -1.720368

H -7.355831 -0.289143 1.322914

H -6.514427 1.171028 1.867754

H -7.775398 1.288308 0.631891

H -5.623644 3.201970 0.706588

H -4.181789 2.238032 1.076782

H -4.145447 3.425464 -0.237923

H -1.623957 1.169502 2.499819

H -2.203721 0.959650 0.103960

ωB97X Energy = -1461.66251143 a.u.

(6*S*,9*R*,12*S*,13*S*,14*R*,15*R*)-**1a**, Conf N

C 3.261743 -1.244180 0.543547

C 2.942151 -0.462159 1.712862

O 2.326307 0.745366 1.500456

C 1.977032 1.215148 0.279462

C 2.243157 0.516443 -0.841207

C 2.930770 -0.746946 -0.673827

C 1.199336 2.536795 0.368160

C -0.262692 2.209692 0.101174

C -1.269846 3.033004 -0.175825

C -2.651243 2.445029 -0.462346

C -2.899162 1.095099 0.194184

C -2.988799 -0.008308 -0.541231

C -3.268453 -1.432985 -0.103598

O -2.768103 -1.766152 1.203020

C -1.516864 -2.461098 1.142966

C -1.421366 -2.987796 -0.276201

C -2.509522 -2.374890 -1.022416

O 3.180397 -1.490716 -1.787394

C 4.341775 -1.086768 -2.521574

C 3.933087 -2.562551 0.764706

C 1.805377 0.949079 -2.214814

O 3.170117 -0.768781 2.862732

C 1.356160 3.179386 1.743853

O 1.750087 3.477495 -0.553947

C -1.196513 4.526855 -0.303049

C -3.031583 1.164510 1.691812

C -4.768169 -1.700171 -0.102321

O -2.595797 -3.739197 -0.618166

C -0.121485 -3.464122 -0.847671

C -0.349099 -1.568886 1.524483

O -2.879184 2.463863 -1.868399

H -0.500897 1.154737 0.186265

H -3.397353 3.137351 -0.057859

H -1.589454 -3.293007 1.850541

H -2.480220 -2.192522 -2.091871

H 5.238333 -1.215093 -1.909952

H 4.263429 -0.045090 -2.842002

H 4.394896 -1.733114 -3.394052

H 4.118009 -3.064589 -0.182524

H 3.308828 -3.207366 1.388084

H 4.881327 -2.429029 1.289352

H 2.508090 1.655218 -2.663238

H 0.817304 1.411116 -2.192505

H 1.739938 0.082985 -2.871538

H 0.948869 2.541493 2.526113

H 2.408753 3.370905 1.956548

H 0.814962 4.124953 1.741062

H 1.405896 3.301262 -1.433728

H -0.177040 4.902868 -0.262761

H -1.652432 4.845180 -1.243673

H -1.772806 4.988789 0.504152

H -3.521399 0.284228 2.098419

H -2.049946 1.247462 2.167774

H -3.600228 2.054331 1.975265

H -4.959219 -2.753640 0.106152

H -5.249791 -1.089688 0.663464

H -5.202582 -1.441256 -1.069953

H -0.272622 -3.822729 -1.865683

H 0.614913 -2.657743 -0.868857

H 0.284776 -4.281022 -0.246669

H -0.557668 -1.067272 2.470341

H 0.560144 -2.163060 1.645628

H -0.167133 -0.810937 0.758727

H -2.119161 2.064865 -2.303002

H -2.923852 0.103307 -1.621203

ωB97X Energy = -1461.66207542 a.u.

(6*S*,9*R*,12*S*,13*S*,14*R*,15*R*)-**1a**, Conf O

C 3.056185 -1.859704 -0.599007

C 3.603304 -0.746012 -1.332364

O 3.301707 0.509640 -0.864785

C 2.523166 0.757163 0.216415

C 1.997537 -0.251408 0.939165

C 2.307096 -1.593699 0.499508

C 2.385887 2.268835 0.476615

C 0.927602 2.706550 0.528155

C 0.010835 2.559066 -0.423250

C -1.392623 3.080712 -0.180684

C -2.418578 2.007242 0.174407

C -2.036766 0.762830 0.452129

C -2.875414 -0.420662 0.900796

O -2.047872 -1.602003 0.928071

C -2.174394 -2.365950 -0.277673

C -3.505386 -1.941687 -0.868191

C -3.935970 -0.764907 -0.129872

O 1.753404 -2.611704 1.217420

C 2.632738 -3.208582 2.176629

C 3.349953 -3.234714 -1.111318

C 1.122748 -0.089980 2.151522

O 4.301277 -0.810265 -2.320653

C 3.093401 2.612770 1.785034

O 3.093001 3.013886 -0.508608

C 0.242680 1.928674 -1.768757

C -3.830041 2.528037 0.230985

C -3.411682 -0.247929 2.314753

O -4.552760 -2.028688 0.110175

C -3.873979 -2.268371 -2.281906

C -1.004944 -2.137220 -1.220031

O -1.840178 3.876459 -1.280010

H 0.641452 3.227112 1.439474

H -1.359946 3.781466 0.658819

H -2.208795 -3.417807 0.022210

H -4.569400 0.004372 -0.556382

H 3.502718 -3.650298 1.684802

H 2.061033 -3.985544 2.678011

H 2.964213 -2.467000 2.908126

H 2.714064 -3.972488 -0.624660

H 4.394909 -3.504238 -0.938672

H 3.180125 -3.278078 -2.188188

H 1.658576 -0.378444 3.059385

H 0.247319 -0.735844 2.065169

H 0.774898 0.932415 2.273690

H 2.640031 2.102138 2.633521

H 4.143442 2.325795 1.720842

H 3.030581 3.689661 1.948759

H 2.728145 2.827544 -1.378813

H 0.313115 2.702481 -2.538197

H 1.149722 1.327166 -1.815042

H -0.591208 1.277741 -2.043519

H -4.514482 1.841007 0.723586

H -3.856681 3.477601 0.771535

H -4.215441 2.727362 -0.771364

H -4.023432 -1.109937 2.583613

H -2.574887 -0.177532 3.011987

H -4.008594 0.659431 2.407590

H -4.872102 -1.889798 -2.501490

H -3.168469 -1.816313 -2.980408

H -3.867647 -3.349253 -2.439851

H -0.069295 -2.335411 -0.694664

H -1.063901 -2.811186 -2.077109

H -0.985856 -1.108292 -1.588958

H -2.042337 3.298455 -2.022211

H -0.980633 0.520803 0.397188

ωB97X Energy = -1461.66196770 a.u.

**Table S3.** Cartesian coordinates and energies of the low-energy conformers calculated at the ωB97X/TZVP PCM/MeOH level of (6*S*,9*R*,12*S*,13*S*,14*R*,15*R*)-**1a**.

(6*S*,9*R*,12*S*,13*S*,14*R*,15*R*)-**1a**, Conf A

C -3.196914 -2.230044 -0.126492

C -3.636164 -1.389970 -1.214866

O -3.471967 -0.038580 -1.057266

C -2.929860 0.542690 0.043658

C -2.519048 -0.199354 1.088427

C -2.649886 -1.632885 0.960465

C -2.866369 2.068443 -0.075708

C -1.444399 2.595885 -0.009045

C -0.371568 2.143049 -0.647326

C 0.976453 2.842190 -0.502615

C 1.946645 1.971006 0.275685

C 2.918941 1.350508 -0.387848

C 3.950852 0.364397 0.130424

O 4.636857 -0.230508 -0.989717

C 4.038921 -1.475654 -1.369714

C 3.318179 -1.954264 -0.124426

C 3.282445 -0.828261 0.794575

O -2.218951 -2.376655 2.019318

C -0.920489 -2.950311 1.842189

C -3.382959 -3.707137 -0.278497

C -1.935749 0.356768 2.354896

O -4.134732 -1.767265 -2.253318

C -3.543483 2.577040 -1.348796

O -3.524271 2.644404 1.059622

C -0.334113 0.927673 -1.534824

C 1.664444 1.868416 1.747617

C 5.017265 1.032525 0.984956

O 4.181983 -1.912630 1.020858

C 2.318462 -3.067844 -0.179031

C 3.108878 -1.324006 -2.561907

O 0.899482 4.100868 0.148030

H -1.352679 3.477218 0.618587

H 1.378664 2.977544 -1.514078

H 4.861851 -2.151209 -1.621678

H 2.502047 -0.692306 1.534217

H -0.909186 -3.641056 0.995174

H -0.691706 -3.489123 2.758466

H -0.172923 -2.166855 1.681524

H -2.673254 -4.121447 -0.999138

H -4.384531 -3.921173 -0.653795

H -3.247999 -4.214221 0.675439

H -2.583927 0.121554 3.201406

H -1.819994 1.435110 2.303116

H -0.963621 -0.095470 2.561223

H -3.050206 2.204576 -2.245721

H -4.592119 2.274547 -1.382901

H -3.487865 3.665849 -1.344301

H -4.456967 2.410345 1.019523

H -0.041438 0.039869 -0.963790

H -1.292835 0.721165 -2.007489

H 0.405378 1.056758 -2.328458

H 1.555414 2.867709 2.172210

H 0.719826 1.345296 1.920256

H 2.448630 1.351225 2.295709

H 5.723352 0.283715 1.346083

H 5.556650 1.767930 0.385955

H 4.573183 1.547547 1.837436

H 1.970373 -3.299637 0.828020

H 1.458702 -2.787678 -0.790902

H 2.765528 -3.967860 -0.606891

H 3.637892 -0.836133 -3.381267

H 2.767515 -2.301192 -2.909645

H 2.232572 -0.722289 -2.307266

H 0.331632 4.673771 -0.375352

H 3.021925 1.543698 -1.452657

ωB97X Energy = -1461.66526368 a.u.

(6*S*,9*R*,12*S*,13*S*,14*R*,15*R*)-**1a**, Conf B

C -3.213150 -2.238603 -0.108864

C -3.622810 -1.432688 -1.233447

O -3.472261 -0.076434 -1.108893

C -2.973089 0.537833 -0.007086

C -2.594204 -0.169492 1.072788

C -2.706718 -1.607667 0.979031

C -2.911093 2.054816 -0.169118

C -1.488712 2.591497 -0.065823

C -0.389737 2.115465 -0.639141

C 0.951490 2.796467 -0.441905

C 1.894303 1.885660 0.332087

C 2.952033 1.379694 -0.297192

C 3.985821 0.392316 0.214486

O 4.738186 -0.120481 -0.902861

C 4.180823 -1.349289 -1.386421

C 3.419285 -1.918199 -0.204368

C 3.323247 -0.852692 0.779343

O -2.298803 -2.319862 2.068173

C -0.997240 -2.899952 1.935209

C -3.383200 -3.720871 -0.227522

C -2.078923 0.432271 2.348113

O -4.084148 -1.842222 -2.277095

C -3.538786 2.525228 -1.481019

O -3.689287 2.560404 0.924065

C -0.317071 0.875145 -1.489902

C 1.488740 1.609908 1.753683

C 4.986992 1.041115 1.157542

O 4.237588 -1.930820 0.974602

C 2.440505 -3.039631 -0.366544

C 3.300305 -1.136934 -2.606490

O 0.771860 4.043761 0.213734

H -1.405555 3.500844 0.522690

H 1.382558 2.965653 -1.436912

H 5.027609 -1.992255 -1.644421

H 2.514329 -0.781469 1.496267

H -0.244778 -2.122543 1.766157

H -0.969476 -3.616309 1.110500

H -0.789079 -3.410633 2.872144

H -4.369787 -3.951960 -0.631291

H -3.278249 -4.202135 0.743424

H -2.646728 -4.149103 -0.912316

H -1.194758 -0.101977 2.697462

H -2.834201 0.351600 3.132853

H -1.831161 1.482246 2.223745

H -2.994082 2.148479 -2.346152

H -4.578251 2.203773 -1.548675

H -3.501077 3.616380 -1.506592

H -3.718318 3.519528 0.846926

H -0.025118 0.005703 -0.890594

H -1.262859 0.644374 -1.976623

H 0.437053 0.993663 -2.271336

H 2.320647 1.280459 2.373798

H 1.067501 2.511532 2.201355

H 0.709696 0.842871 1.790748

H 5.701656 0.295325 1.507635

H 5.527096 1.830779 0.632800

H 4.483281 1.485713 2.016940

H 2.027776 -3.314260 0.604771

H 1.619653 -2.742964 -1.022064

H 2.925127 -3.917309 -0.799906

H 3.853519 -0.583840 -3.366268

H 2.998748 -2.095442 -3.033689

H 2.399182 -0.572610 -2.352859

H 1.637370 4.451132 0.307197

H 3.132746 1.681609 -1.326144

ωB97X Energy = -1461.66423270 a.u.

(6*S*,9*R*,12*S*,13*S*,14*R*,15*R*)-**1a**, Conf C

C 3.259058 -1.985517 -0.242071

C 3.720594 -0.971316 -1.155597

O 3.363435 0.324663 -0.866191

C 2.587387 0.693392 0.183916

C 2.115571 -0.224274 1.049481

C 2.484782 -1.602082 0.804208

C 2.472962 2.227884 0.258033

C 1.043222 2.693491 0.463294

C 0.048129 2.576589 -0.409390

C -1.321958 3.103777 -0.035551

C -2.338044 2.025453 0.330545

C -1.960358 0.758302 0.479642

C -2.781447 -0.450999 0.886634

O -1.982002 -1.638794 0.703669

C -2.230389 -2.248360 -0.569761

C -3.614395 -1.767416 -0.962342

C -3.948389 -0.677028 -0.059189

O 2.073578 -2.509151 1.731553

C 0.950576 -3.317082 1.354353

C 3.702073 -3.392500 -0.497511

C 1.286662 0.070933 2.267378

O 4.401769 -1.144052 -2.141845

C 3.364701 2.713579 1.404730

O 2.916500 2.815590 -0.959278

C 0.170096 1.961289 -1.776651

C -3.729528 2.560483 0.539558

C -3.169450 -0.418862 2.357525

O -4.551484 -1.959527 0.106977

C -4.135016 -1.940103 -2.355369

C -1.169631 -1.882520 -1.595427

O -1.834488 3.962377 -1.057687

H 0.848154 3.188368 1.411260

H -1.218200 3.758514 0.834706

H -2.230743 -3.329553 -0.400519

H -4.614015 0.134246 -0.329958

H 1.146662 -3.842889 0.416352

H 0.051690 -2.702435 1.256122

H 0.815688 -4.042170 2.153566

H 4.762831 -3.410259 -0.750958

H 3.161493 -3.831806 -1.339834

H 3.541023 -4.014048 0.381806

H 0.347002 -0.485795 2.232403

H 1.043933 1.125669 2.356290

H 1.816497 -0.241917 3.168645

H 3.046040 2.298825 2.362268

H 4.400182 2.415604 1.227419

H 3.315838 3.802391 1.457273

H 3.729971 2.379006 -1.233496

H -0.765963 1.482771 -2.074469

H 0.400945 2.730352 -2.517131

H 0.963092 1.216381 -1.826838

H -3.690572 3.483374 1.123529

H -4.200738 2.812302 -0.413114

H -4.376743 1.859516 1.062743

H -3.766295 -1.298368 2.601942

H -2.266771 -0.422031 2.971398

H -3.739131 0.479651 2.596176

H -5.147539 -1.542886 -2.425510

H -3.504506 -1.413184 -3.072880

H -4.155082 -2.997800 -2.627718

H -0.177316 -2.102959 -1.197463

H -1.305300 -2.459464 -2.512197

H -1.210334 -0.819786 -1.845807

H -2.048096 3.430322 -1.830600

H -0.915412 0.508541 0.324376

ωB97X Energy = -1461.66376458 a.u.

(6*S*,9*R*,12*S*,13*S*,14*R*,15*R*)-**1a**, Conf D

C 3.572412 1.655172 -0.443776

C 4.208881 0.398894 -0.752565

O 3.645085 -0.737533 -0.233350

C 2.550460 -0.742260 0.566365

C 1.910409 0.398627 0.874447

C 2.424704 1.624594 0.294305

C 2.189272 -2.154504 1.038898

C 0.788691 -2.582156 0.647945

C 0.153753 -2.395510 -0.501958

C -1.226752 -2.987840 -0.730948

C -2.301457 -1.913655 -0.920269

C -2.527039 -1.052242 0.067360

C -3.638081 -0.020036 0.151120

O -3.734370 0.801750 -1.028108

C -2.992655 2.017438 -0.875807

C -2.892489 2.229334 0.623862

C -3.303868 0.979094 1.243843

O 1.677639 2.703371 0.597614

C 1.518566 3.764153 -0.352005

C 4.299745 2.891765 -0.896739

C 0.687140 0.462213 1.742256

O 5.206488 0.244931 -1.427657

C 3.184533 -3.199647 0.532957

O 2.191878 -2.169579 2.473555

C 0.676380 -1.626051 -1.686361

C -3.061991 -1.989505 -2.210794

C -4.993062 -0.677649 0.370861

O -4.178710 2.103414 1.247077

C -1.951094 3.232790 1.214144

C -1.633495 1.944529 -1.551429

O -1.625928 -3.924770 0.261212

H 0.312059 -3.186398 1.413799

H -1.168409 -3.568615 -1.657007

H -3.594988 2.806604 -1.335762

H -2.951045 0.636935 2.212087

H 2.316136 4.499549 -0.272180

H 0.570642 4.239360 -0.107242

H 1.476222 3.362873 -1.365874

H 5.335942 2.629681 -1.102705

H 4.292886 3.657233 -0.121258

H 3.884711 3.322787 -1.809844

H -0.193472 0.712888 1.145501

H 0.796818 1.240742 2.498492

H 0.511761 -0.485579 2.242427

H 3.193878 -3.248969 -0.555533

H 4.197156 -2.980278 0.877261

H 2.878781 -4.169747 0.925603

H 3.091804 -2.009459 2.774768

H 1.764252 -1.620043 -1.741292

H 0.304684 -2.063187 -2.616280

H 0.334951 -0.586844 -1.659831

H -2.371859 -1.879998 -3.053379

H -3.528262 -2.974365 -2.311295

H -3.823073 -1.218112 -2.294380

H -5.759528 0.087400 0.500794

H -5.254690 -1.303308 -0.483198

H -4.963752 -1.309979 1.260539

H -2.094528 3.282125 2.293563

H -0.913623 2.956160 1.018186

H -2.132138 4.224503 0.792867

H -1.755523 1.638465 -2.591077

H -1.145609 2.921540 -1.538513

H -0.981496 1.221535 -1.053803

H -2.062318 -3.445643 0.971576

H -1.882811 -1.107816 0.945959

ωB97X Energy = -1461.66360731 a.u.

(6*S*,9*R*,12*S*,13*S*,14*R*,15*R*)-**1a**, Conf E

C -3.141097 1.702480 0.827965

C -3.806585 0.478559 1.204925

O -3.441565 -0.658076 0.527957

C -2.491318 -0.696902 -0.437315

C -1.850114 0.421328 -0.828148

C -2.217041 1.651111 -0.161841

C -2.293668 -2.102209 -1.016298

C -0.865291 -2.595225 -0.835033

C -0.132985 -2.586402 0.272993

C 1.294526 -3.099969 0.253501

C 2.283702 -2.005364 0.670988

C 2.619134 -1.068237 -0.210159

C 3.620771 0.056397 -0.021486

O 3.479808 0.744663 1.236318

C 2.645403 1.900212 1.101690

C 2.758122 2.292277 -0.360829

C 3.351817 1.149959 -1.041476

O -1.549191 2.782494 -0.528929

C -2.167009 3.516605 -1.591937

C -3.525878 2.946705 1.564105

C -0.798862 0.453670 -1.903178

O -4.658895 0.361832 2.057774

C -3.279548 -3.111360 -0.438155

O -2.611202 -2.059763 -2.413590

C -0.582576 -2.077696 1.615052

C 2.812452 -2.123824 2.071865

C 5.049155 -0.464425 -0.104829

O 4.126551 2.320070 -0.790816

C 1.856905 3.324514 -0.964178

C 1.209421 1.616361 1.514919

O 1.590307 -3.631804 -1.028593

H -0.431673 -3.020907 -1.734272

H 1.363442 -3.903592 1.000580

H 3.077813 2.669837 1.747816

H 3.172284 0.901565 -2.082886

H -3.142350 3.896206 -1.277307

H -2.286761 2.891952 -2.480710

H -1.503559 4.348295 -1.818112

H -4.574322 3.194447 1.383866

H -2.905924 3.785962 1.254953

H -3.409631 2.801215 2.639726

H -1.224686 0.736758 -2.869874

H -0.298552 -0.507882 -2.008811

H -0.034009 1.190805 -1.660443

H -3.138784 -3.239195 0.633595

H -4.306641 -2.797715 -0.625820

H -3.103797 -4.068176 -0.929827

H -1.945803 -1.545415 -2.879934

H -0.097998 -1.125087 1.850206

H -1.658333 -1.930640 1.675695

H -0.302704 -2.784676 2.400165

H 1.993098 -2.122258 2.796359

H 3.332046 -3.079762 2.189743

H 3.488576 -1.312894 2.328079

H 5.753099 0.366237 -0.041434

H 5.243723 -1.161515 0.711408

H 5.202195 -0.992402 -1.048264

H 2.045322 3.406643 -2.035004

H 0.805636 3.078383 -0.805806

H 2.047420 4.297302 -0.504024

H 1.195181 1.187152 2.517936

H 0.617370 2.533960 1.523747

H 0.737639 0.906374 0.829288

H 2.533015 -3.815427 -1.057800

H 2.174538 -1.132277 -1.202648

ωB97X Energy = -1461.66349542 a.u.

(6*S*,9*R*,12*S*,13*S*,14*R*,15*R*)-**1a**, Conf F

C 2.461118 -2.015812 0.707775

C 1.805585 -1.026002 1.524509

O 2.007245 0.282059 1.182301

C 2.801982 0.699729 0.160888

C 3.526079 -0.180311 -0.557325

C 3.309314 -1.582775 -0.258080

C 2.725907 2.223063 0.080769

C 1.290692 2.703554 0.254241

C 0.243420 2.299416 -0.458866

C -1.135545 2.850350 -0.135853

C -2.009325 1.771911 0.481476

C -3.059623 1.293403 -0.177351

C -4.069858 0.258465 0.280922

O -3.531790 -0.768030 1.131658

C -3.148382 -1.925440 0.381137

C -3.967454 -1.850351 -0.894557

C -4.545824 -0.516687 -0.935520

O 3.963931 -2.475302 -1.052748

C 5.085544 -3.120346 -0.440288

C 2.152377 -3.453529 0.988552

C 4.501541 0.162655 -1.650864

O 1.082340 -1.244306 2.474217

C 3.609215 2.810781 1.183096

O 3.220010 2.626067 -1.197360

C 0.305002 1.261714 -1.543641

C -1.550067 1.340953 1.847383

C -5.211255 0.924652 1.036886

O -5.356726 -1.640629 -0.598706

C -3.649676 -2.706275 -2.080743

C -1.651784 -1.967793 0.121850

O -1.758965 3.387974 -1.294948

H 1.140200 3.442007 1.038095

H -1.018456 3.642759 0.614484

H -3.448322 -2.793173 0.977039

H -4.800971 0.003492 -1.853231

H 5.488635 -3.805193 -1.182358

H 4.783733 -3.678158 0.448567

H 5.846802 -2.384844 -0.166774

H 2.650624 -3.799031 1.897997

H 2.463587 -4.086451 0.158855

H 1.079354 -3.579058 1.142710

H 4.034038 0.064558 -2.633585

H 5.342899 -0.528913 -1.623944

H 4.870531 1.177186 -1.553202

H 3.272722 2.478870 2.166946

H 4.644626 2.500769 1.036026

H 3.558168 3.901396 1.149607

H 2.943459 3.537429 -1.330990

H 0.180649 0.261937 -1.115306

H -0.501150 1.411461 -2.262745

H 1.258141 1.287480 -2.069370

H -2.309703 0.763455 2.366049

H -0.648806 0.723344 1.779084

H -1.289521 2.217037 2.448206

H -5.983595 0.190697 1.271137

H -4.834577 1.357696 1.965246

H -5.647171 1.726344 0.437499

H -4.364599 -2.512276 -2.880299

H -2.645782 -2.495963 -2.452270

H -3.702211 -3.764717 -1.815436

H -1.108390 -1.857165 1.062289

H -1.367040 -2.920114 -0.331671

H -1.351177 -1.159002 -0.548083

H -1.182864 4.073078 -1.644802

H -3.276090 1.728445 -1.149466

ωB97X Energy = -1461.66346577 a.u.

(6*S*,9*R*,12*S*,13*S*,14*R*,15*R*)-**1a**, Conf G

C 3.522754 -2.082503 0.251555

C 3.568343 -1.391147 1.517418

O 3.291025 -0.048901 1.498926

C 2.965220 0.648745 0.380300

C 2.923929 0.049043 -0.823969

C 3.231825 -1.363022 -0.860312

C 2.702223 2.129168 0.673978

C 1.299028 2.556977 0.285295

C 0.146668 1.967341 0.579321

C -1.176897 2.574892 0.130371

C -2.017819 1.560461 -0.632825

C -3.188274 1.189976 -0.120913

C -4.197492 0.189491 -0.655927

O -5.228178 -0.013220 0.330695

C -4.947848 -1.150878 1.155461

C -4.020670 -2.013853 0.321373

C -3.576927 -1.191245 -0.792631

O 3.166236 -1.963726 -2.081882

C 4.436891 -2.198318 -2.698503

C 3.794461 -3.554298 0.261626

C 2.577798 0.740162 -2.111129

O 3.828848 -1.884845 2.593488

C 2.970380 2.487003 2.135953

O 3.563618 2.916872 -0.158735

C -0.005788 0.691176 1.361822

C -1.408486 1.061525 -1.913506

C -4.896131 0.683196 -1.913930

O -4.548448 -2.215505 -0.998231

C -3.258333 -3.151704 0.925654

C -4.336750 -0.751312 2.488295

O -1.034174 3.784311 -0.599705

H 1.291872 3.499527 -0.252709

H -1.728368 2.858447 1.031222

H -5.903551 -1.657696 1.319104

H -2.622124 -1.330201 -1.286454

H 4.960059 -1.253737 -2.869131

H 4.233107 -2.679924 -3.651644

H 5.057267 -2.851927 -2.081224

H 3.479074 -4.013225 -0.674054

H 3.258432 -4.027584 1.085231

H 4.857886 -3.758453 0.410456

H 3.411773 0.680911 -2.814001

H 2.346336 1.788870 -1.953028

H 1.723970 0.252248 -2.584682

H 2.312451 1.944070 2.813037

H 4.003937 2.265905 2.410059

H 2.792602 3.555783 2.256719

H 4.474271 2.745347 0.101063

H -0.838847 0.777814 2.064455

H -0.238115 -0.148123 0.698670

H 0.884008 0.433305 1.932403

H -1.137097 1.894861 -2.568080

H -0.487698 0.506285 -1.709830

H -2.073500 0.417645 -2.483738

H -5.597121 -0.073958 -2.267271

H -5.446215 1.598445 -1.689696

H -4.177449 0.903944 -2.703634

H -2.676495 -3.658145 0.155640

H -2.576065 -2.794738 1.698528

H -3.941871 -3.873807 1.377958

H -4.973129 -0.012244 2.976188

H -4.251884 -1.617498 3.147537

H -3.341825 -0.318495 2.354810

H -0.529121 3.611314 -1.400208

H -3.506621 1.649915 0.811183

ωB97X Energy = -1461.66338233 a.u.

(6*S*,9*R*,12*S*,13*S*,14*R*,15*R*)-**1a**, Conf H

C 2.585624 -1.875429 0.539064

C 3.046811 -0.866000 1.460258

O 2.913423 0.440734 1.062363

C 2.370523 0.824864 -0.118580

C 1.943267 -0.080476 -1.020333

C 2.065369 -1.473949 -0.647110

C 2.299554 2.346726 -0.258514

C 0.844349 2.802608 -0.325164

C -0.106365 2.647181 0.588862

C -1.533975 3.078075 0.311589

C -2.482656 1.894110 0.117619

C -2.031436 0.756146 -0.402759

C -2.822315 -0.490802 -0.755567

O -3.760078 -0.880431 0.264026

C -3.175063 -1.822776 1.173224

C -2.043244 -2.466600 0.392700

C -1.856318 -1.661002 -0.802861

O 1.570083 -2.404819 -1.512479

C 2.487794 -2.817023 -2.531780

C 2.717545 -3.302379 0.968638

C 1.348590 0.280322 -2.353899

O 3.538661 -1.061899 2.550092

C 3.062145 3.072596 0.847640

O 2.937111 2.620618 -1.510943

C 0.072079 2.046794 1.957685

C -3.906355 2.183519 0.494840

C -3.589752 -0.315062 -2.057203

O -2.506608 -2.925628 -0.886569

C -0.980321 -3.281307 1.061577

C -2.705766 -1.160751 2.456860

O -1.672029 3.979111 -0.782155

H 0.588131 3.302508 -1.255104

H -1.878232 3.644273 1.184282

H -3.953444 -2.558885 1.395585

H -0.907311 -1.577582 -1.324446

H 3.359772 -3.299770 -2.083327

H 2.810639 -1.965973 -3.136193

H 1.953971 -3.527648 -3.157746

H 2.304442 -3.973508 0.218186

H 2.193926 -3.465927 1.913160

H 3.766371 -3.557510 1.134684

H 2.120671 0.333832 -3.123831

H 0.855383 1.248207 -2.323692

H 0.624493 -0.474505 -2.660752

H 2.629312 2.893871 1.829891

H 4.106502 2.759850 0.859246

H 3.014928 4.144824 0.646243

H 2.944426 3.575211 -1.634390

H -0.827790 1.509876 2.262773

H 0.912468 1.358702 2.023604

H 0.233712 2.842683 2.690766

H -3.982388 2.319011 1.578525

H -4.230186 3.119555 0.031949

H -4.586205 1.385242 0.208706

H -4.075361 -1.252433 -2.331944

H -4.348323 0.461450 -1.949020

H -2.908099 -0.017797 -2.857003

H -0.270659 -3.641532 0.315662

H -0.435385 -2.679724 1.791845

H -1.416236 -4.138965 1.578898

H -2.387189 -1.911448 3.182898

H -1.870211 -0.483306 2.266767

H -3.523898 -0.587364 2.894481

H -1.673836 3.463033 -1.594354

H -0.969378 0.693940 -0.631744

ωB97X Energy = -1461.66334244 a.u.

(6*S*,9*R*,12*S*,13*S*,14*R*,15*R*)-**1a**, Conf I

C 3.164250 -1.800264 -0.526924

C 3.722379 -0.685140 -1.249364

O 3.332683 0.568056 -0.841082

C 2.480636 0.813138 0.186102

C 1.981193 -0.195888 0.924635

C 2.359115 -1.535863 0.532489

C 2.299252 2.328549 0.388785

C 0.840132 2.723025 0.535667

C -0.107567 2.584855 -0.385650

C -1.515025 3.056456 -0.073900

C -2.506383 1.941568 0.251993

C -2.083411 0.698579 0.468798

C -2.877079 -0.528260 0.881588

O -2.006049 -1.678509 0.872872

C -2.094243 -2.398663 -0.362842

C -3.438825 -2.005997 -0.944986

C -3.920259 -0.876814 -0.165229

O 1.804923 -2.547369 1.256452

C 2.692310 -3.192452 2.176875

C 3.511831 -3.175364 -1.005657

C 1.095408 -0.041702 2.128883

O 4.493047 -0.742864 -2.181795

C 3.097430 2.744987 1.627281

O 2.789024 3.038367 -0.743754

C 0.097745 2.003438 -1.756177

C -3.929806 2.418607 0.358882

C -3.421939 -0.423369 2.298711

O -4.486083 -2.172821 0.023529

C -3.786583 -2.293900 -2.372341

C -0.929179 -2.084601 -1.286175

O -2.010481 3.897724 -1.117934

H 0.580768 3.189517 1.482969

H -1.475288 3.713660 0.799987

H -2.086916 -3.462100 -0.104811

H -4.582195 -0.117259 -0.565575

H 3.538849 -3.645683 1.657017

H 2.110754 -3.965496 2.672866

H 3.058817 -2.476738 2.917261

H 2.824705 -3.913050 -0.593425

H 4.531260 -3.450927 -0.723566

H 3.457174 -3.214847 -2.094298

H 1.604837 -0.412066 3.021647

H 0.182823 -0.627924 2.006504

H 0.815125 0.993286 2.303387

H 2.741070 2.234928 2.523486

H 4.153680 2.504163 1.490383

H 2.997745 3.822023 1.770441

H 3.656896 2.689595 -0.973794

H 0.157222 2.805310 -2.496443

H 1.015857 1.425392 -1.833889

H -0.741316 1.360328 -2.035061

H -4.597645 1.669615 0.778575

H -3.979038 3.309230 0.991205

H -4.319517 2.707626 -0.619318

H -3.999484 -1.317348 2.537181

H -2.589917 -0.343083 3.000566

H -4.054680 0.455669 2.420064

H -4.802605 -1.959283 -2.580979

H -3.103884 -1.777499 -3.048588

H -3.723346 -3.365699 -2.573531

H 0.010653 -2.265740 -0.761652

H -0.954490 -2.724856 -2.170361

H -0.949471 -1.041642 -1.613027

H -2.180497 3.358404 -1.896582

H -1.022753 0.492267 0.376383

ωB97X Energy = -1461.66320099 a.u.

(6*S*,9*R*,12*S*,13*S*,14*R*,15*R*)-**1a**, Conf J

C -5.199387 -1.183986 -0.249010

C -5.377262 0.178584 -0.688602

O -4.405396 1.076124 -0.329920

C -3.299799 0.755616 0.388544

C -3.068262 -0.511334 0.782399

C -4.070001 -1.494024 0.434703

C -2.398195 1.975553 0.635275

C -1.213236 1.849372 -0.298962

C 0.098628 1.848083 -0.074786

C 1.018699 1.702091 -1.281515

C 1.892080 0.465958 -1.183032

C 3.217190 0.563657 -1.189326

C 4.227520 -0.567896 -1.152584

O 3.881631 -1.628946 -0.243070

C 4.479564 -1.426168 1.041837

C 5.681790 -0.538528 0.775772

C 5.528234 -0.033418 -0.578909

O -3.844794 -2.763670 0.875038

C -3.406711 -3.679984 -0.134149

C -6.288400 -2.158192 -0.573636

C -1.852962 -0.963605 1.541021

O -6.307934 0.604971 -1.338261

C -3.127423 3.285469 0.325620

O -1.976236 1.986282 1.992037

C 0.806850 1.972838 1.245971

C 1.110299 -0.816007 -1.099863

C 4.413232 -1.187174 -2.530618

O 6.476950 -1.060895 -0.299664

C 6.414120 0.140782 1.890719

C 3.506343 -0.813240 2.034340

O 1.828886 2.861474 -1.435943

H -1.541056 1.749749 -1.332544

H 0.392050 1.578832 -2.174820

H 4.800678 -2.411965 1.392369

H 5.931044 0.914404 -0.921578

H -3.260612 -4.636494 0.361544

H -2.462098 -3.344648 -0.570979

H -4.154902 -3.785155 -0.922599

H -7.261889 -1.706295 -0.379617

H -6.192959 -3.060417 0.028628

H -6.268325 -2.439258 -1.629783

H -2.118589 -1.237520 2.564172

H -1.098530 -0.184613 1.584554

H -1.422754 -1.849317 1.070545

H -3.401672 3.359553 -0.725482

H -4.036581 3.375760 0.924033

H -2.453601 4.105720 0.574590

H -2.751655 2.131903 2.542757

H 0.231057 2.542625 1.967978

H 0.992708 0.981913 1.673861

H 1.778346 2.447294 1.096501

H 0.651091 -0.923105 -0.112173

H 0.295695 -0.811320 -1.829801

H 1.735561 -1.688254 -1.267430

H 5.202549 -1.939461 -2.497420

H 3.485238 -1.657476 -2.859310

H 4.681576 -0.417840 -3.257383

H 7.256077 0.706409 1.491865

H 5.755643 0.826457 2.425719

H 6.794130 -0.594843 2.603460

H 2.592520 -1.408699 2.068049

H 3.939885 -0.794211 3.036442

H 3.243507 0.207154 1.746440

H 1.239180 3.615005 -1.525427

H 3.644364 1.558710 -1.276149

ωB97X Energy = -1461.66275616 a.u.

(6*S*,9*R*,12*S*,13*S*,14*R*,15*R*)-**1a**, Conf K

C 3.237744 -1.279081 0.526860

C 2.942276 -0.498643 1.703920

O 2.340191 0.716883 1.504999

C 1.988818 1.198702 0.287676

C 2.238294 0.507084 -0.841138

C 2.904466 -0.769591 -0.685039

C 1.222877 2.524320 0.387386

C -0.237805 2.214309 0.107675

C -1.232121 3.046156 -0.184337

C -2.613430 2.467750 -0.487205

C -2.885340 1.126406 0.176903

C -2.976919 0.017732 -0.550280

C -3.276748 -1.399022 -0.100858

O -2.780514 -1.726617 1.209121

C -1.539386 -2.439726 1.155301

C -1.450651 -2.979926 -0.259222

C -2.530505 -2.359143 -1.010960

O 3.125614 -1.515881 -1.803043

C 4.279697 -1.131879 -2.558089

C 3.887867 -2.610157 0.735099

C 1.795289 0.954928 -2.207302

O 3.179104 -0.815575 2.849588

C 1.368331 3.154169 1.773829

O 1.710899 3.426389 -0.604301

C -1.140812 4.537385 -0.324561

C -3.036614 1.208185 1.672079

C -4.779746 -1.646942 -0.097137

O -2.634740 -3.718764 -0.595214

C -0.156722 -3.477689 -0.826023

C -0.359150 -1.560831 1.529242

O -2.812587 2.470990 -1.898424

H -0.488207 1.163267 0.201741

H -3.362116 3.169982 -0.104709

H -1.623704 -3.264506 1.869959

H -2.498305 -2.186043 -2.081891

H 5.184869 -1.272968 -1.961914

H 4.212299 -0.090583 -2.881078

H 4.308031 -1.781702 -3.429201

H 4.060508 -3.107999 -0.216646

H 3.256002 -3.249600 1.356480

H 4.840225 -2.496122 1.256869

H 1.587291 0.084029 -2.828713

H 2.563839 1.551523 -2.702646

H 0.899501 1.568822 -2.149041

H 0.944112 2.515584 2.547299

H 2.419464 3.331689 2.012696

H 0.836855 4.105709 1.771804

H 2.599615 3.695483 -0.350541

H -1.707460 5.015282 0.480531

H -0.114290 4.893439 -0.296325

H -1.595896 4.852842 -1.266672

H -2.059564 1.262986 2.161223

H -3.581626 2.116340 1.943455

H -3.558780 0.345464 2.076544

H -4.984311 -2.695559 0.122825

H -5.254014 -1.021882 0.661446

H -5.210368 -1.393230 -1.067852

H -0.312465 -3.845397 -1.840093

H 0.589105 -2.680267 -0.856024

H 0.240115 -4.292928 -0.216457

H -0.561003 -1.047775 2.470432

H 0.541338 -2.166939 1.656523

H -0.165994 -0.812744 0.756566

H -2.032508 2.087670 -2.311390

H -2.894550 0.119761 -1.629914

ωB97X Energy = -1461.66243576 a.u.

(6*S*,9*R*,12*S*,13*S*,14*R*,15*R*)-**1a**, Conf L

C 5.305249 0.878626 -0.270051

C 5.415381 -0.551036 -0.125714

O 4.243461 -1.264543 -0.188648

C 3.022138 -0.714549 -0.404657

C 2.874221 0.610949 -0.588635

C 4.077440 1.412370 -0.486223

C 1.959074 -1.817744 -0.490060

C 0.635552 -1.395256 0.116842

C 0.443715 -0.975045 1.361468

C -0.930400 -0.532665 1.843167

C -2.084414 -0.932142 0.940293

C -2.624736 -0.041590 0.114163

C -3.758497 -0.227997 -0.876870

O -4.123474 1.051433 -1.433217

C -5.224376 1.636059 -0.728211

C -5.927621 0.465338 -0.068740

C -5.031562 -0.674713 -0.178655

O 3.957381 2.758690 -0.659934

C 3.545559 3.464243 0.515528

C 6.568463 1.674717 -0.171862

C 1.568669 1.284096 -0.919020

O 6.433942 -1.180463 0.056259

C 1.776978 -2.187226 -1.964440

O 2.392220 -2.966606 0.234783

C 1.528827 -0.810872 2.387197

C -2.498403 -2.368750 1.107643

C -3.354168 -1.103865 -2.054280

O -6.183783 -0.582813 -1.015744

C -6.953005 0.672465 1.002270

C -4.765450 2.686452 0.269442

O -0.826057 0.874053 2.050685

H -0.213978 -1.449977 -0.557343

H -1.101569 -1.020416 2.814399

H -5.869558 2.094763 -1.483653

H -5.021988 -1.490231 0.535004

H 3.430224 4.506294 0.227752

H 4.307624 3.377593 1.294513

H 2.593587 3.081422 0.892310

H 7.029558 1.546606 0.809908

H 6.371239 2.732140 -0.334982

H 7.292806 1.333287 -0.914120

H 1.764151 2.199844 -1.475722

H 1.005919 1.540669 -0.018168

H 0.928390 0.647794 -1.527446

H 1.423448 -1.333969 -2.546370

H 2.724375 -2.525906 -2.389114

H 1.046459 -2.994078 -2.043270

H 3.291937 -3.175138 -0.039905

H 2.443060 -1.330239 2.108544

H 1.751555 0.250456 2.528609

H 1.196413 -1.200201 3.352988

H -1.621923 -3.021002 1.078813

H -2.972078 -2.513764 2.083127

H -3.191576 -2.710252 0.342005

H -4.196789 -1.218593 -2.737094

H -2.527006 -0.633150 -2.588732

H -3.025695 -2.088789 -1.720686

H -7.355818 -0.288444 1.322221

H -6.514161 1.171467 1.867421

H -7.774842 1.289161 0.631302

H -5.622754 3.202351 0.706565

H -4.181152 2.238091 1.076967

H -4.144302 3.425605 -0.237624

H -1.623831 1.168297 2.500279

H -2.203043 0.959105 0.104449

ωB97X Energy = -1461.66239545 a.u.

(6*S*,9*R*,12*S*,13*S*,14*R*,15*R*)-**1a**, Conf M

C 3.261039 -1.248139 0.543809

C 2.941243 -0.466564 1.713434

O 2.326712 0.741733 1.501260

C 1.979003 1.212657 0.280287

C 2.245319 0.514386 -0.840607

C 2.931517 -0.749813 -0.673480

C 1.202758 2.535143 0.369184

C -0.259528 2.209831 0.101391

C -1.265488 3.034424 -0.176185

C -2.647414 2.448182 -0.463730

C -2.897772 1.098973 0.193265

C -2.989596 -0.004584 -0.541650

C -3.271775 -1.428402 -0.102799

O -2.769864 -1.761750 1.203156

C -1.520399 -2.459683 1.141435

C -1.428348 -2.987278 -0.277635

C -2.516468 -2.372464 -1.022353

O 3.181407 -1.493001 -1.787426

C 4.344253 -1.089986 -2.519726

C 3.931055 -2.567232 0.764687

C 1.809343 0.948431 -2.214347

O 3.167967 -0.774175 2.863220

C 1.359673 3.176988 1.745212

O 1.754992 3.475605 -0.552259

C -1.190270 4.528198 -0.303182

C -3.030097 1.169096 1.690874

C -4.772055 -1.692332 -0.098713

O -2.604885 -3.736347 -0.617333

C -0.130377 -3.466529 -0.851000

C -0.349964 -1.570025 1.520719

O -2.873920 2.466626 -1.870004

H -0.499101 1.155172 0.186482

H -3.392997 3.141716 -0.060351

H -1.593842 -3.291105 1.849492

H -2.488571 -2.190664 -2.091952

H 5.239851 -1.219898 -1.907024

H 4.267664 -0.047942 -2.839443

H 4.397740 -1.735711 -3.392647

H 4.115646 -3.069188 -0.182654

H 3.306086 -3.211589 1.387813

H 4.879325 -2.434794 1.289561

H 2.513073 1.654421 -2.661416

H 0.821595 1.411213 -2.192750

H 1.744009 0.082920 -2.871850

H 0.951443 2.539182 2.527048

H 2.412378 3.367335 1.958390

H 0.819453 4.123117 1.742569

H 1.411130 3.300003 -1.432285

H -0.170388 4.903020 -0.262240

H -1.645337 4.847216 -1.243988

H -1.766460 4.990690 0.503776

H -3.523034 0.290598 2.097595

H -2.048259 1.248615 2.166979

H -3.595684 2.060916 1.974148

H -4.964974 -2.745215 0.110993

H -5.251024 -1.080180 0.667395

H -5.207588 -1.433267 -1.065805

H -0.283818 -3.825033 -1.868704

H 0.607732 -2.661742 -0.873421

H 0.275025 -4.284204 -0.250476

H -0.555930 -1.067453 2.466636

H 0.558071 -2.166253 1.640816

H -0.167361 -0.812913 0.754267

H -2.114391 2.065497 -2.303496

H -2.924628 0.106310 -1.621693

ωB97X Energy = -1461.66194892 a.u.

**Fig. S12.** HRESIMS spectrum of compound **1b**;


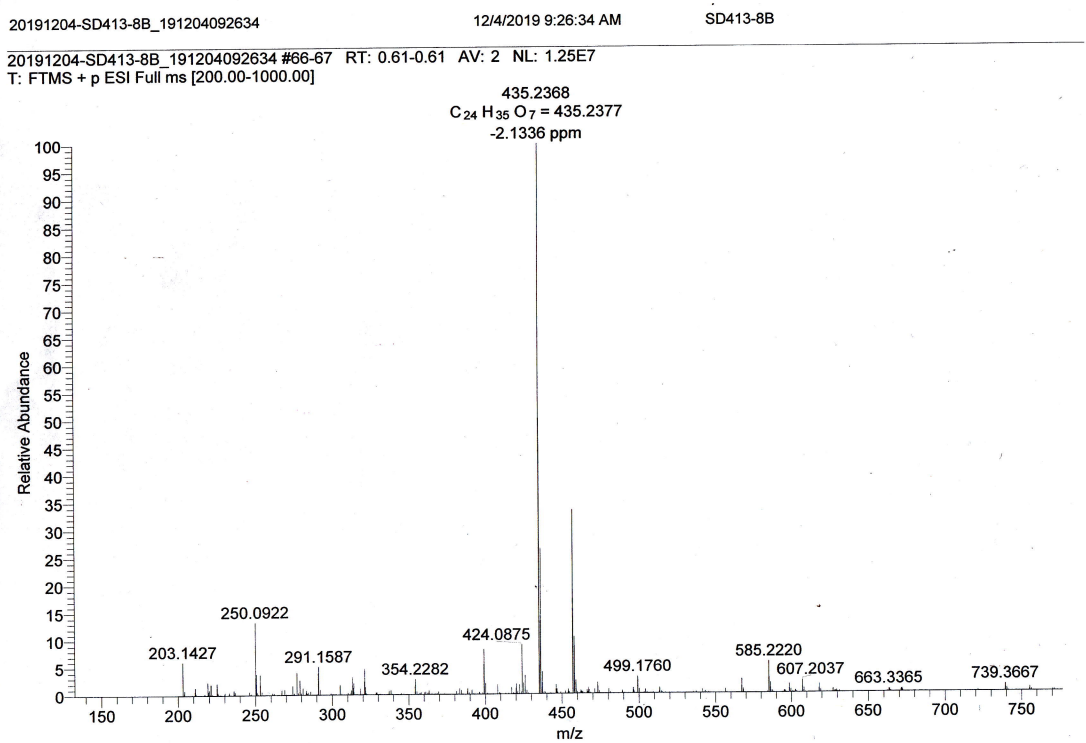


**Fig. S13.** ^1^H NMR spectrum of compound **1b** (Recorded in DMSO-*d*_6_);


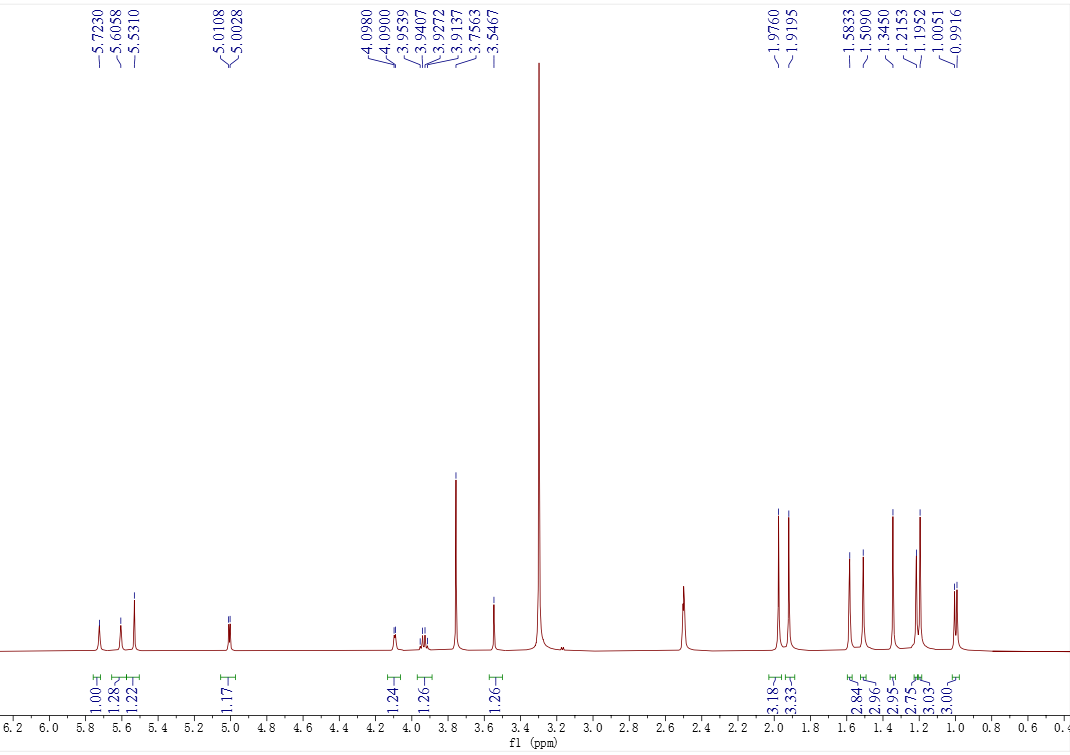


**Fig. S14.** ^13^C NMR and DEPT spectra of compound **1b** (Recorded in DMSO-*d*_6_);


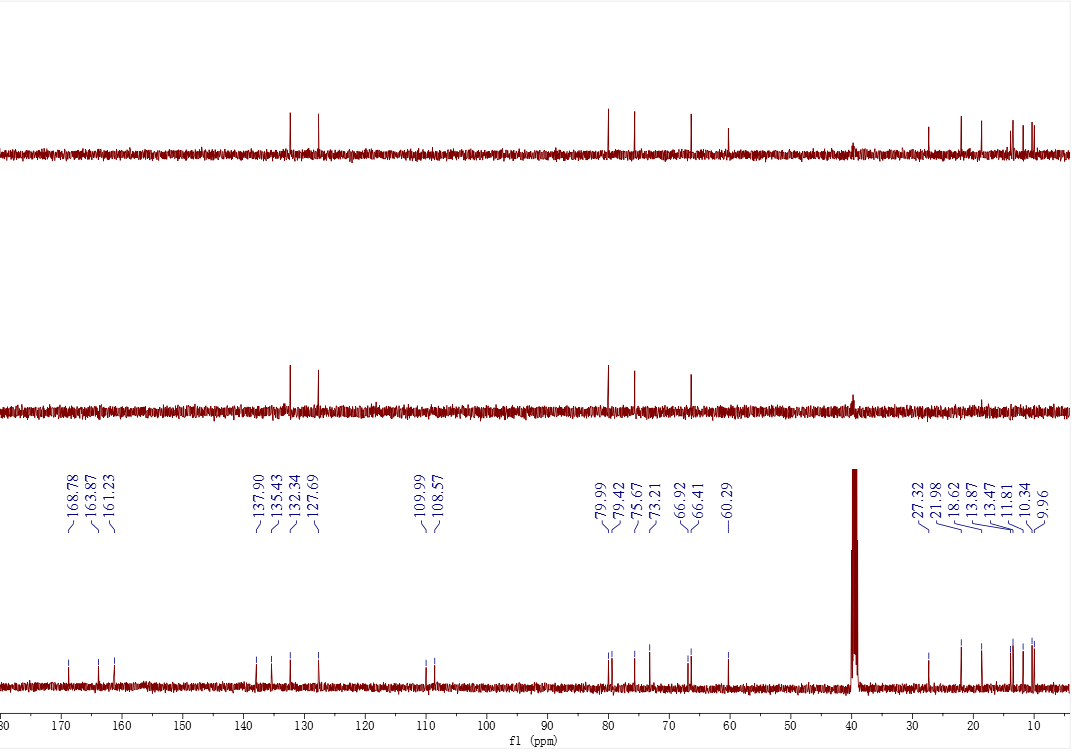


**Fig. S15.** ^1^H-^1^H COSY spectrum of compound **1b** (Recorded in DMSO-*d*_6_);


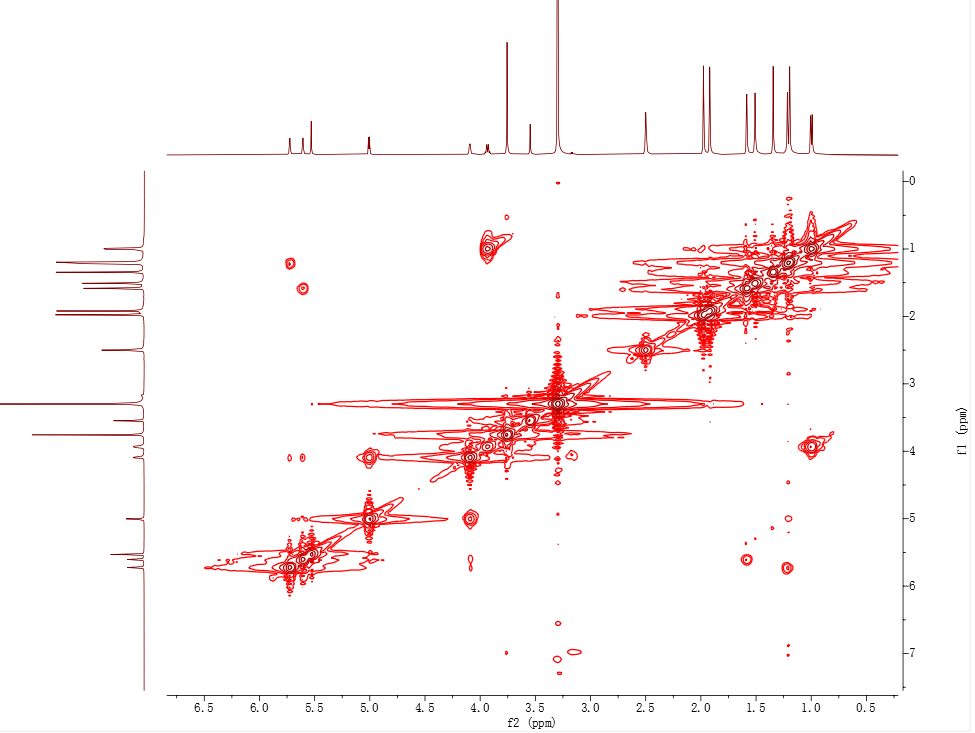


**Fig. S16.** NOESY spectrum of compound **1b** (Recorded in DMSO-*d*_6_);


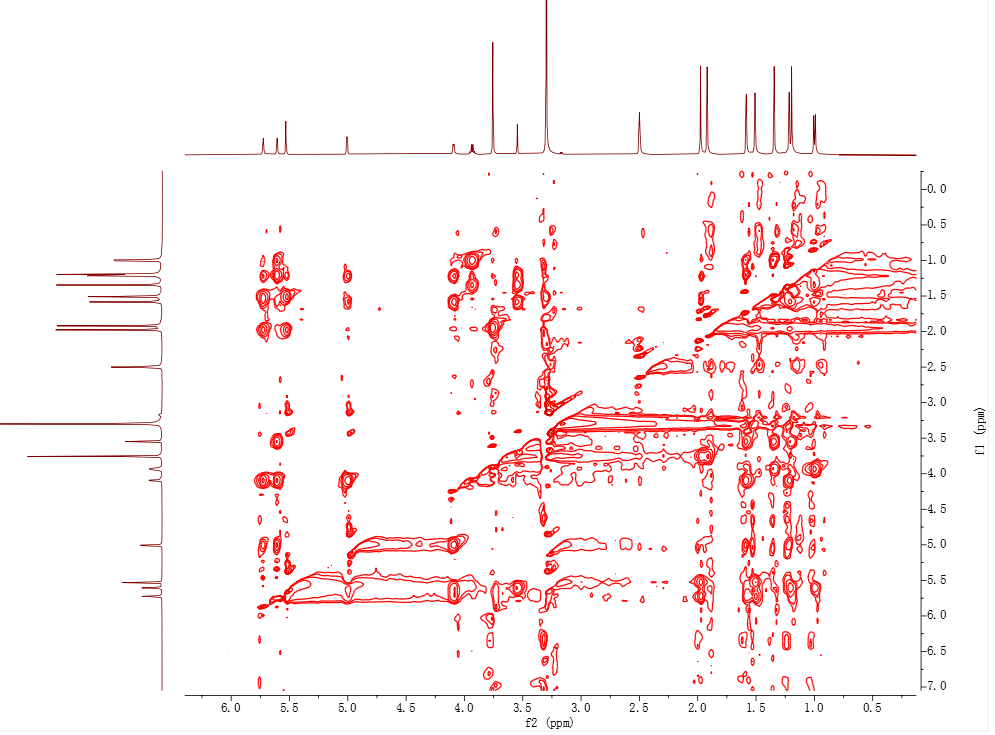


**Fig. S17.** ^1^H NMR spectrum of the (*S)*-MTPA ester compound **1b** (Recorded in DMSO-*d*_6_);

**Fig. S18.** ^1^H NMR spectrum of the (*R)*-MTPA ester compound **1b** (Recorded in DMSO-*d*_6_);

**Fig. S19.** Chiral HPLC chromatogram of compounds **1a/1b** (Column: CHIRALPAK IG, 4.6 × 250 mm; Temperature: 25 ℃; Flow rate: 1mL/min; Mobile phase: 75% *n*-hexane-isopropanol; 41 bar).


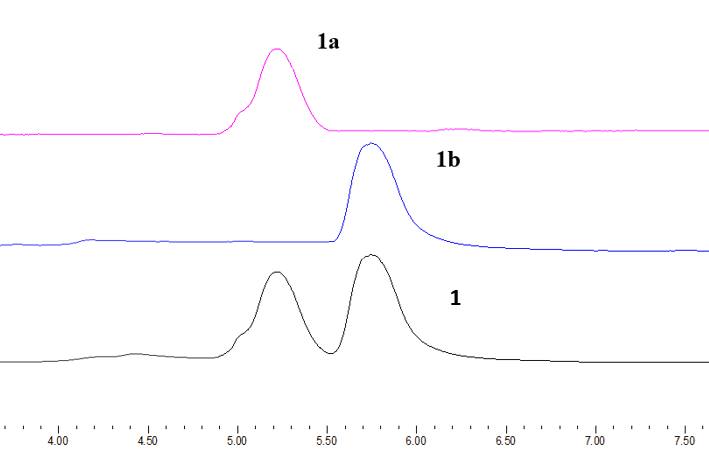


**Fig. S20.** HRESIMS spectrum of compound **2a**;


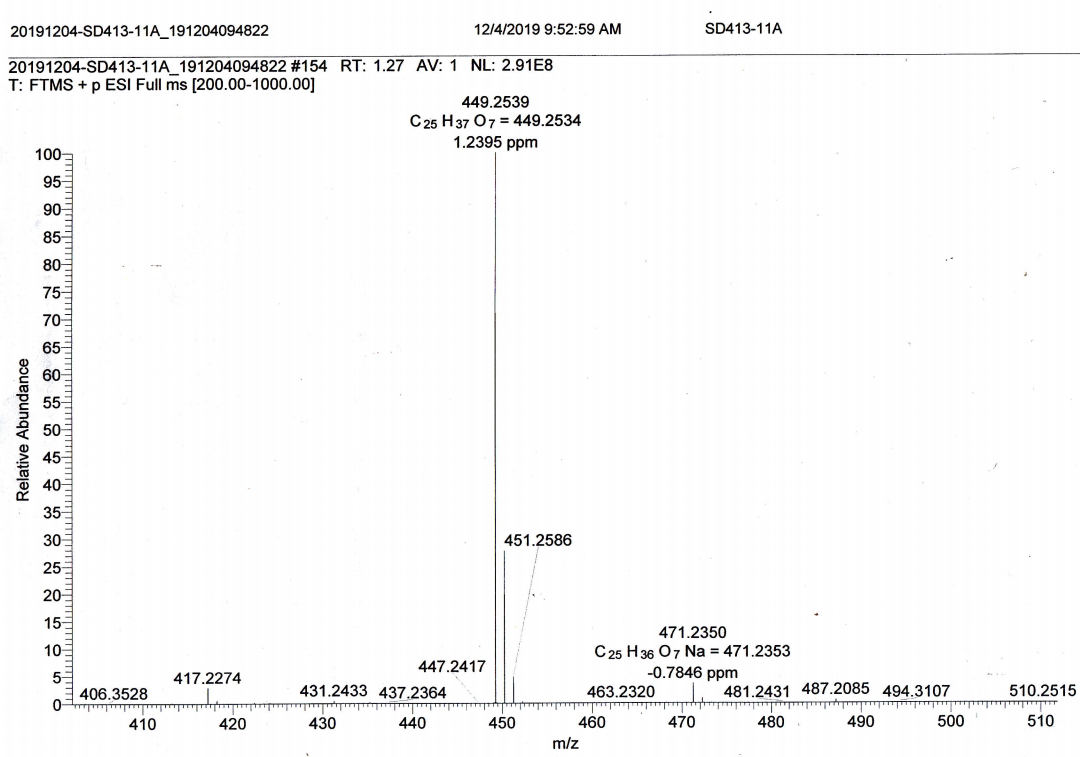


**Fig. S21.** ^1^H NMR spectrum of compound **2a** (Recorded in DMSO-*d*_6_);


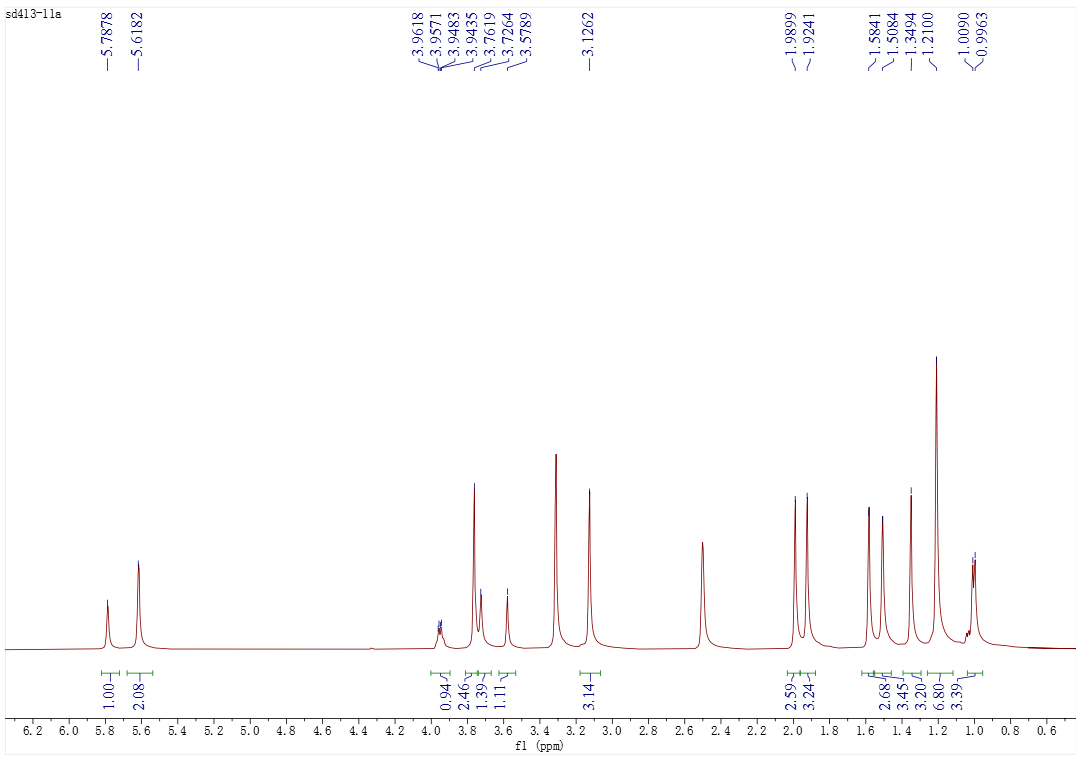


**Fig. S22.** ^13^C NMR and DEPT spectra of compound **2a** (Recorded in DMSO-*d*_6_);


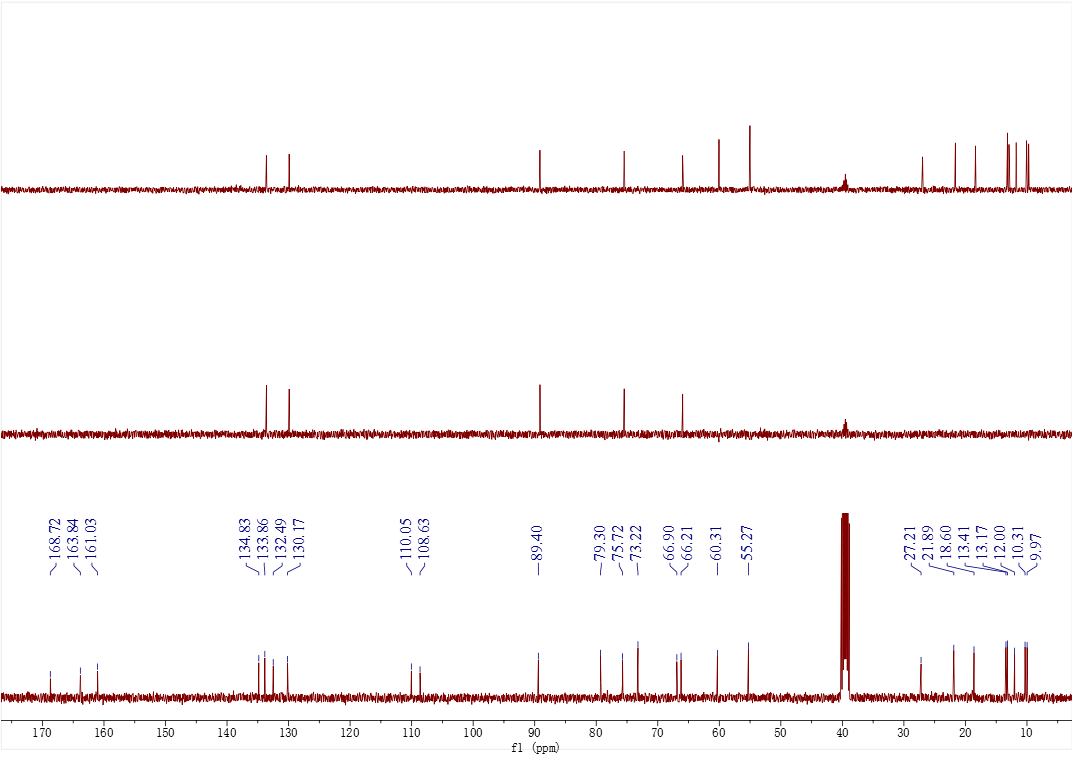


**Fig. S23.** ^1^H-^1^H COSY spectrum of compound **2a** (Recorded in DMSO-*d*_6_);


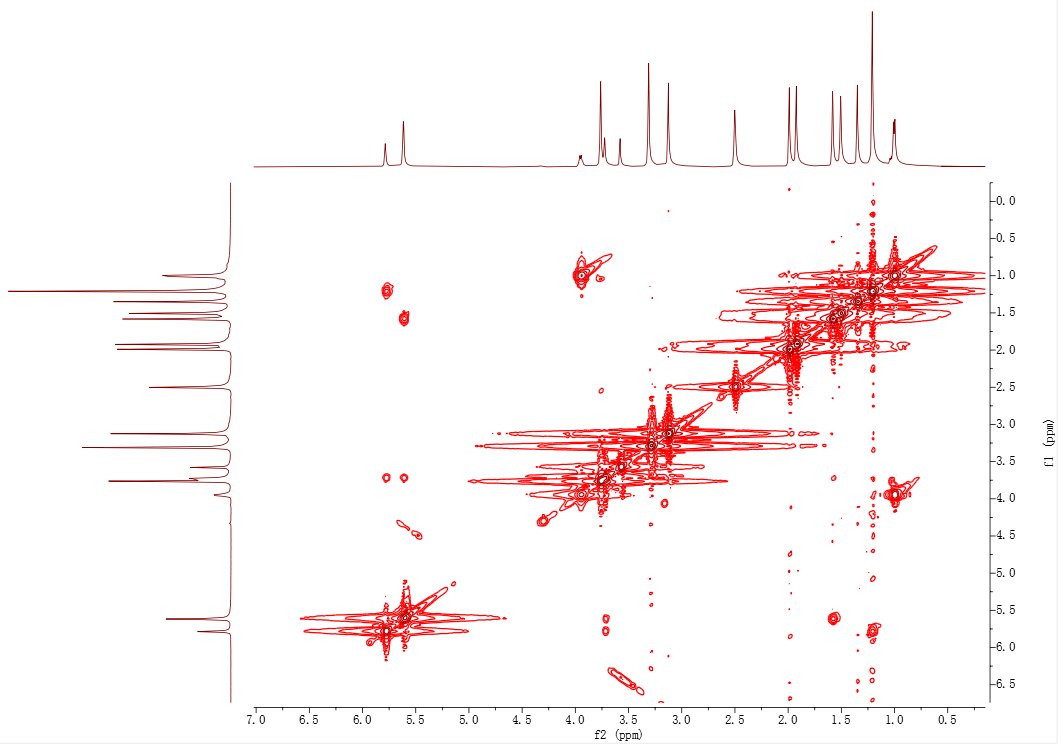


**Fig. S24.** NOESY spectrum of compound **2a** (Recorded in DMSO-*d*_6_);


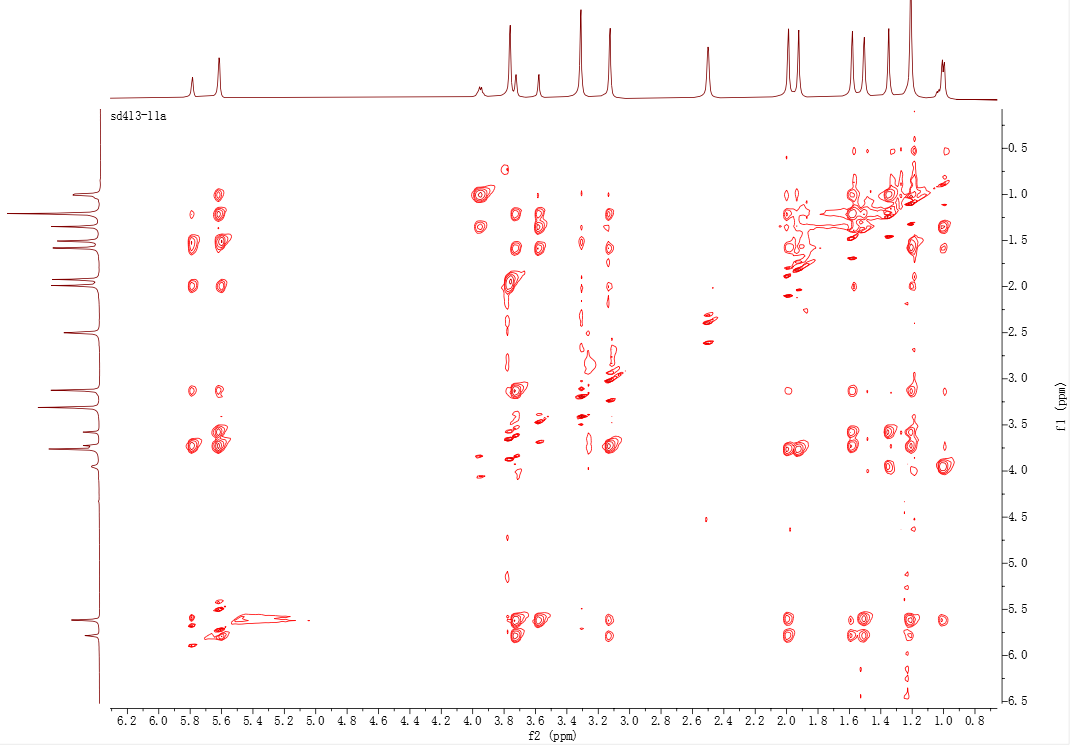


**Fig. S25.** HRSEIMS spectrum of compound **2b**;


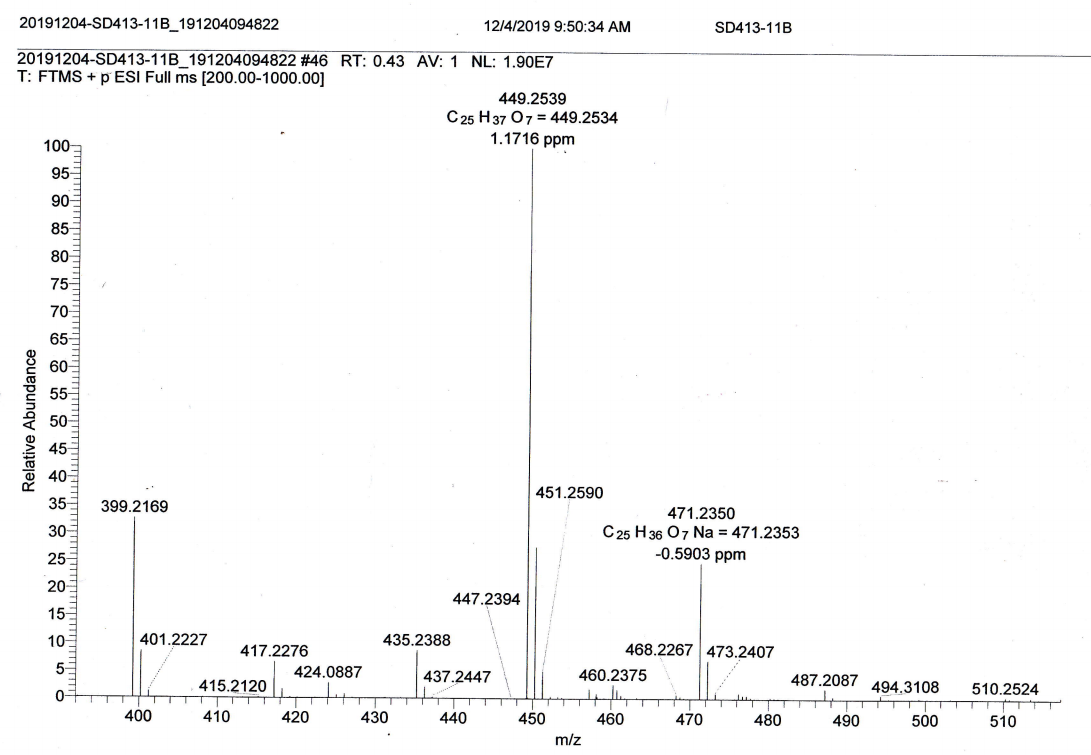


**Fig. S26.** ^1^H NMR spectrum of compound **2b** (Recorded in DMSO-*d*_6_);


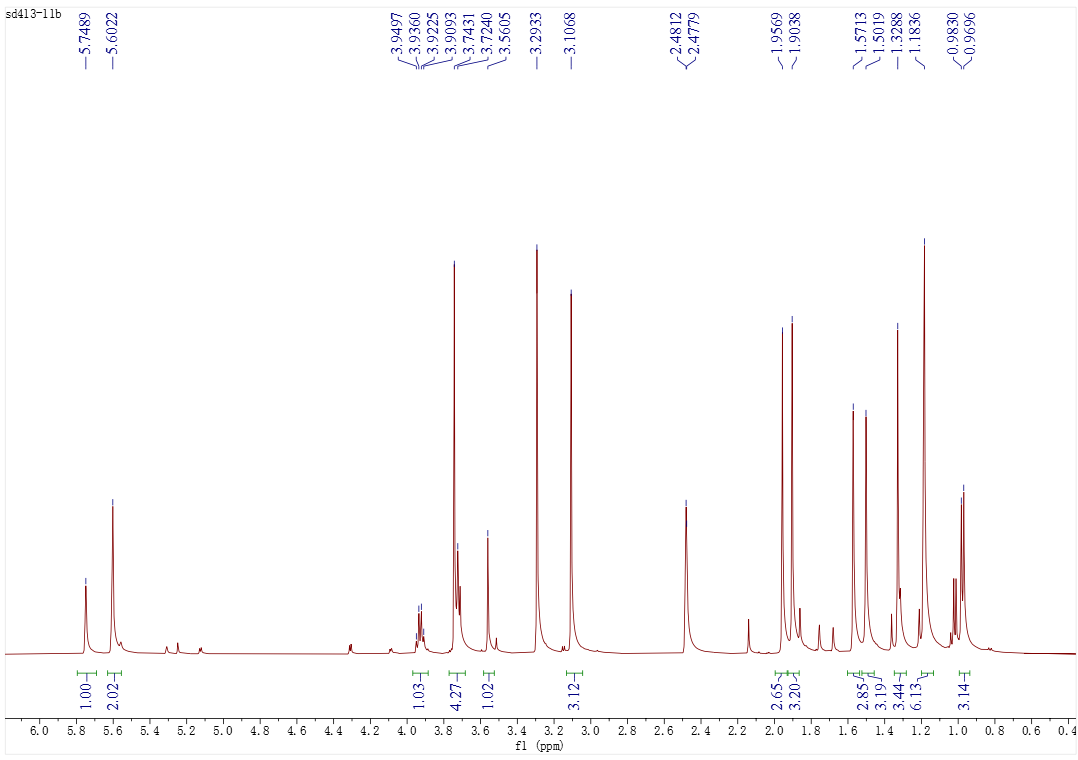


**Fig. S27.** ^13^C NMR and DEPT spectra of compound **2b** (Recorded in DMSO-*d*_6_);


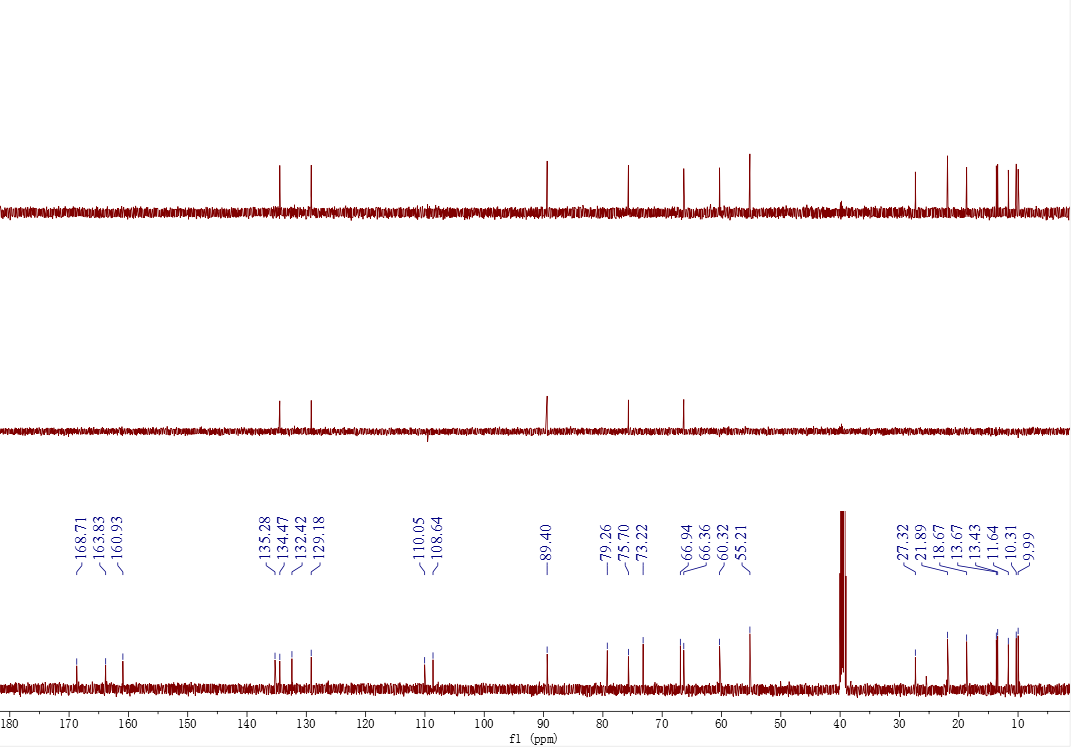


**Fig S28.** ^1^H-^1^H COSY spectrum of compound **2b** (Recorded in DMSO-*d*_6_);


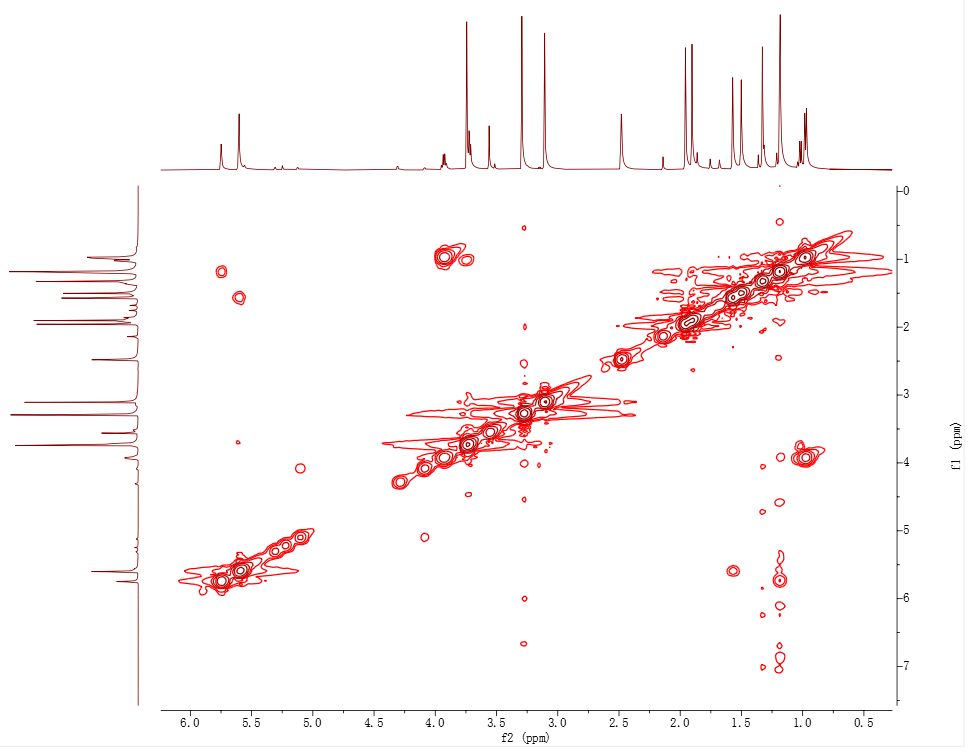


**Fig. S29.** NOESY spectrum of compound **2b** (Recorded in DMSO-*d*_6_);


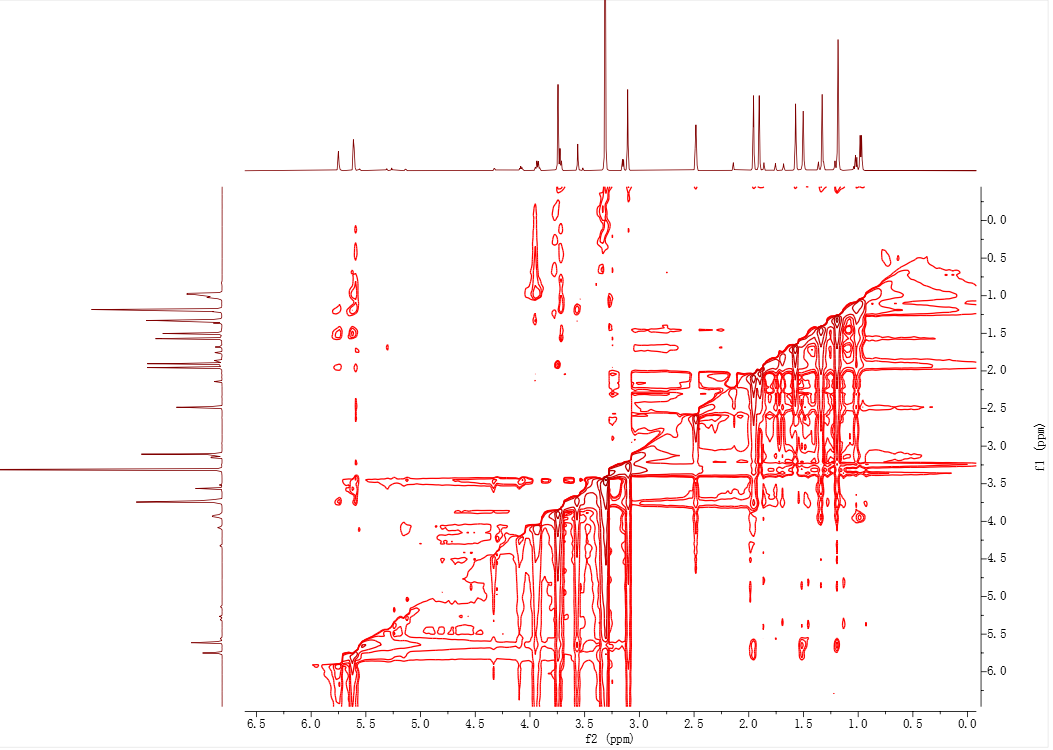


**Fig. S30.** Chiral HPLC chromatogram of compounds **2a/2b** (Column: CHIRALPAK IG, 4.6 × 250 mm; Temperature: 25 ℃; Flow rate: 1mL/min; Mobile phase: 85% *n*-hexane-isopropanol; 43 bar);


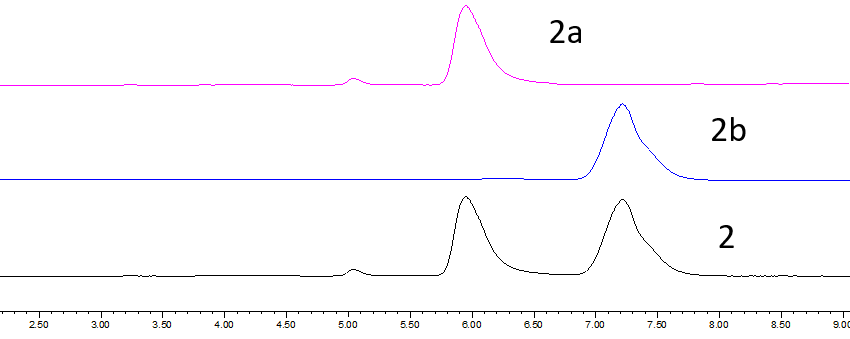


**Fig. S31.** ^1^H NMR Spectrum of **3** (Recorded in DMSO-*d*_6_);


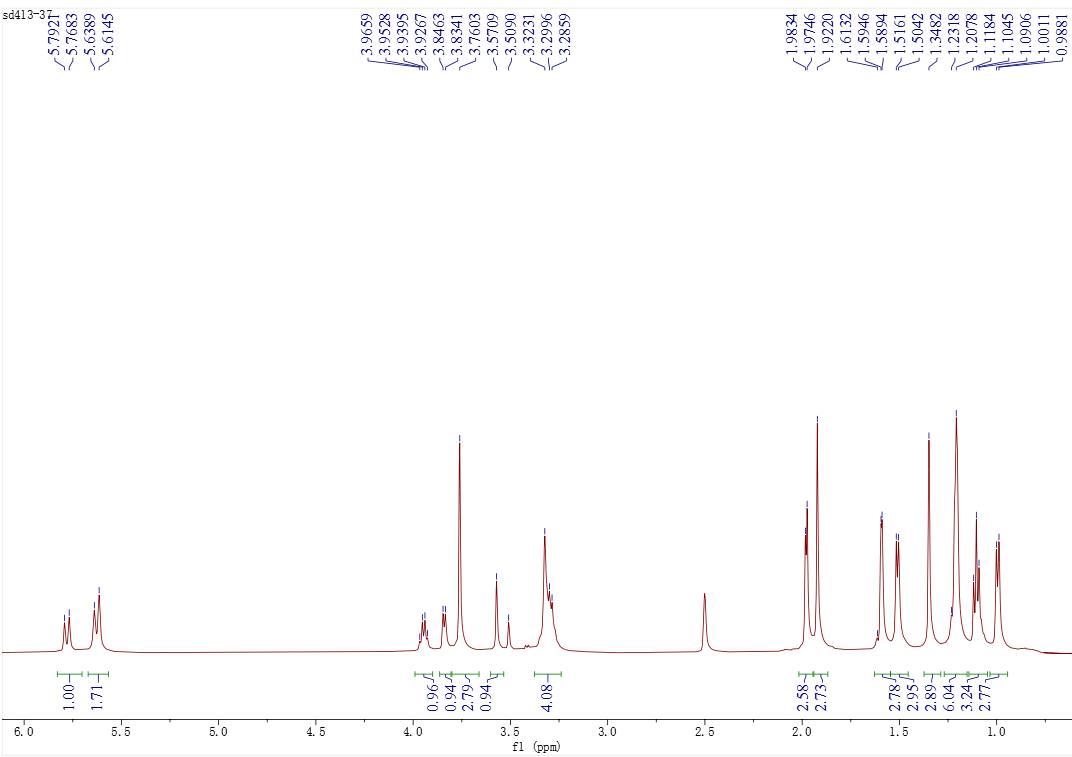


**Fig. S32.** ^13^C NMR and DEPT spectra of compound **3** (Recorded in DMSO-*d*_6_);


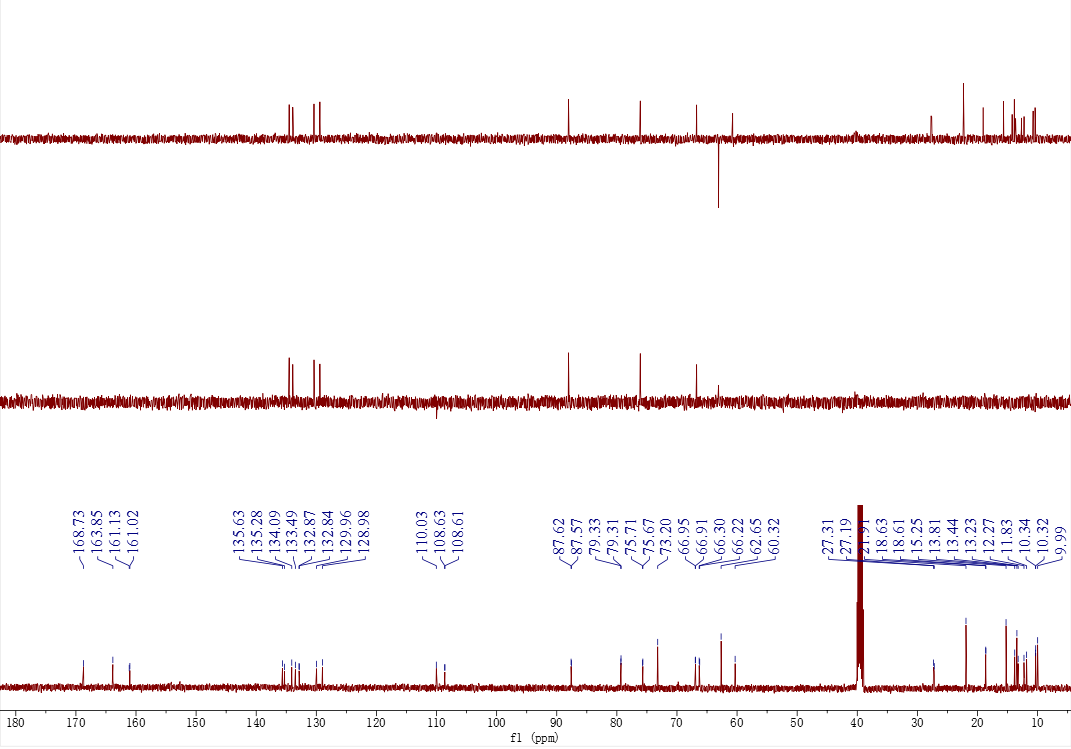


**Fig. S33.** ^1^H-^1^H COSY spectrum of compound **3** (Recorded in DMSO-*d*_6_);


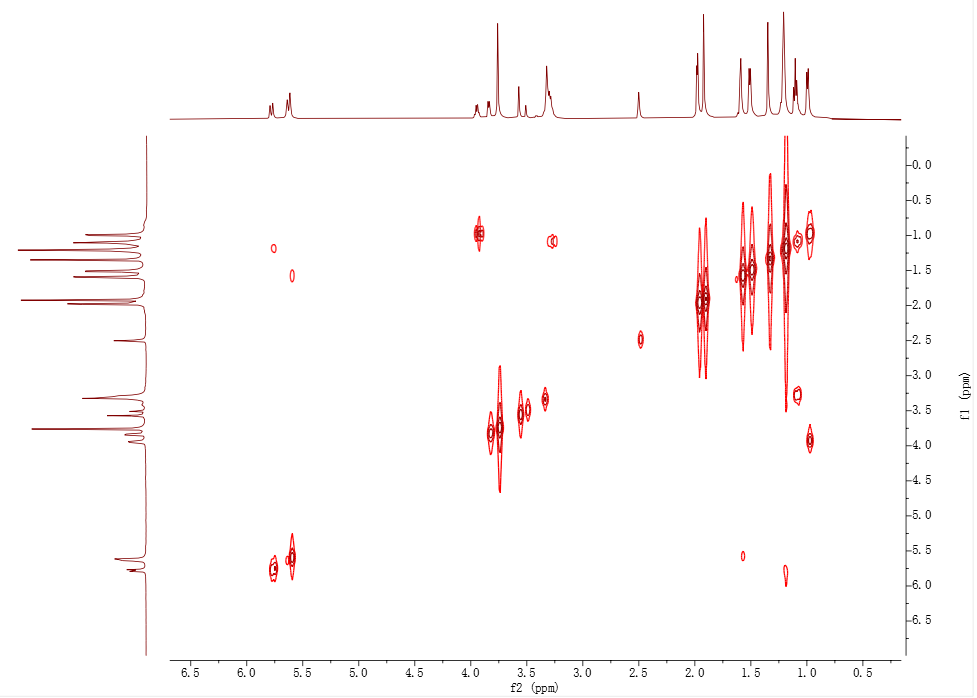


**Fig. S34.** HSQC spectrum of compound **3** (Recorded in DMSO-*d*_6_);


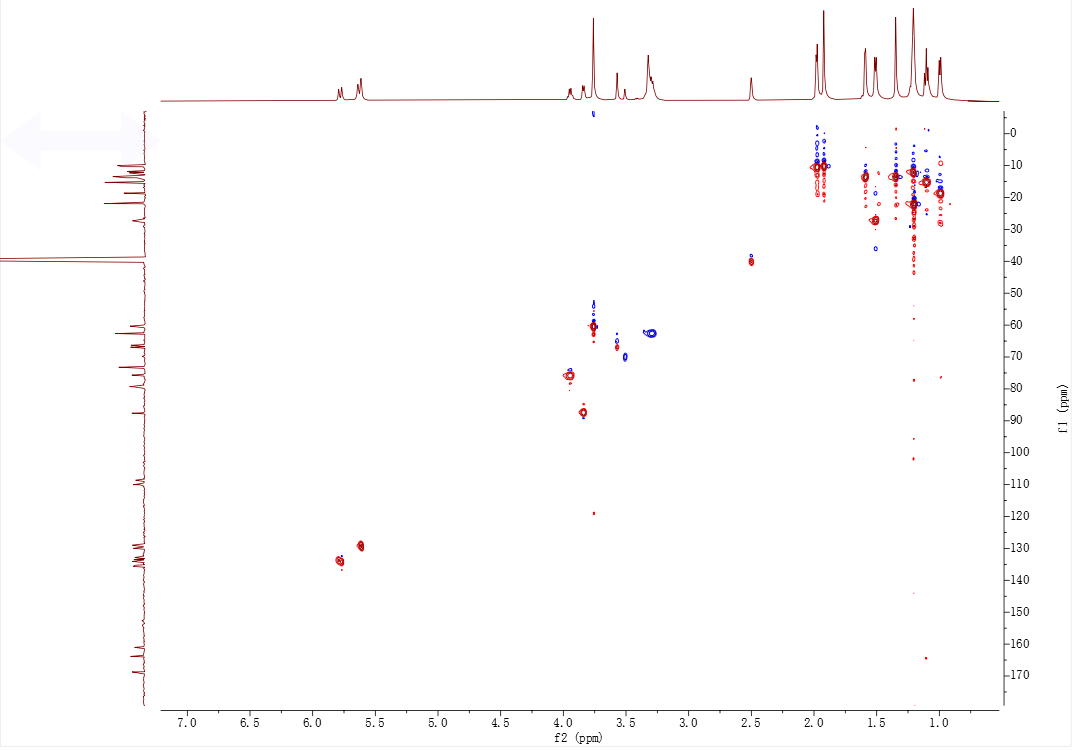


**Fig. S35.** HMBC spectrum of compound **3** (Recorded in DMSO-*d*_6_);


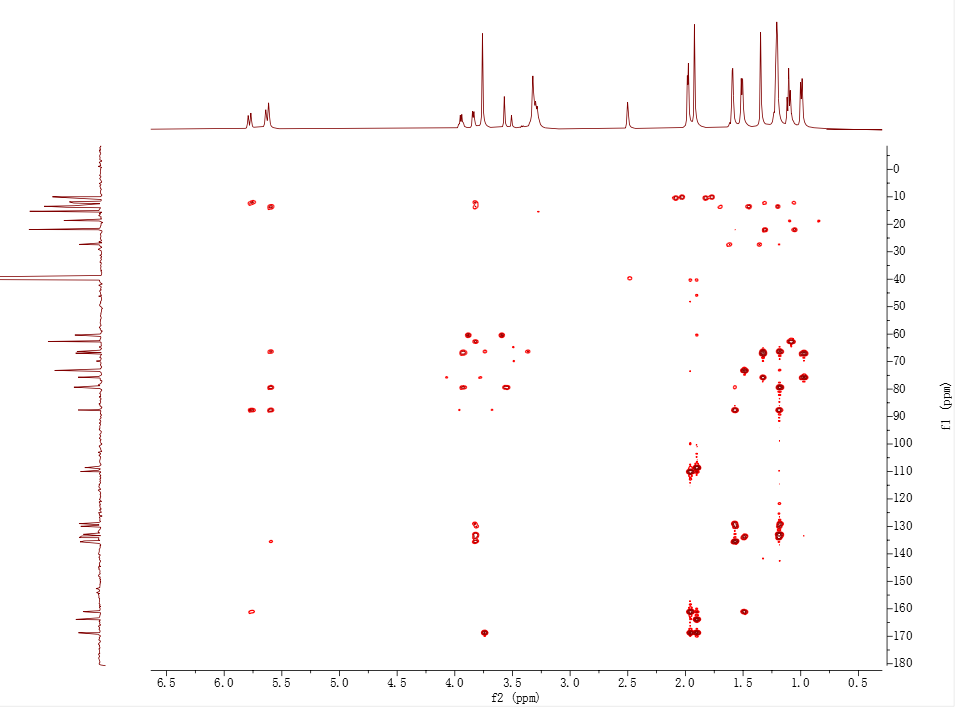


**Fig. S36.** HRESIMS spectrum of compound **3a**;


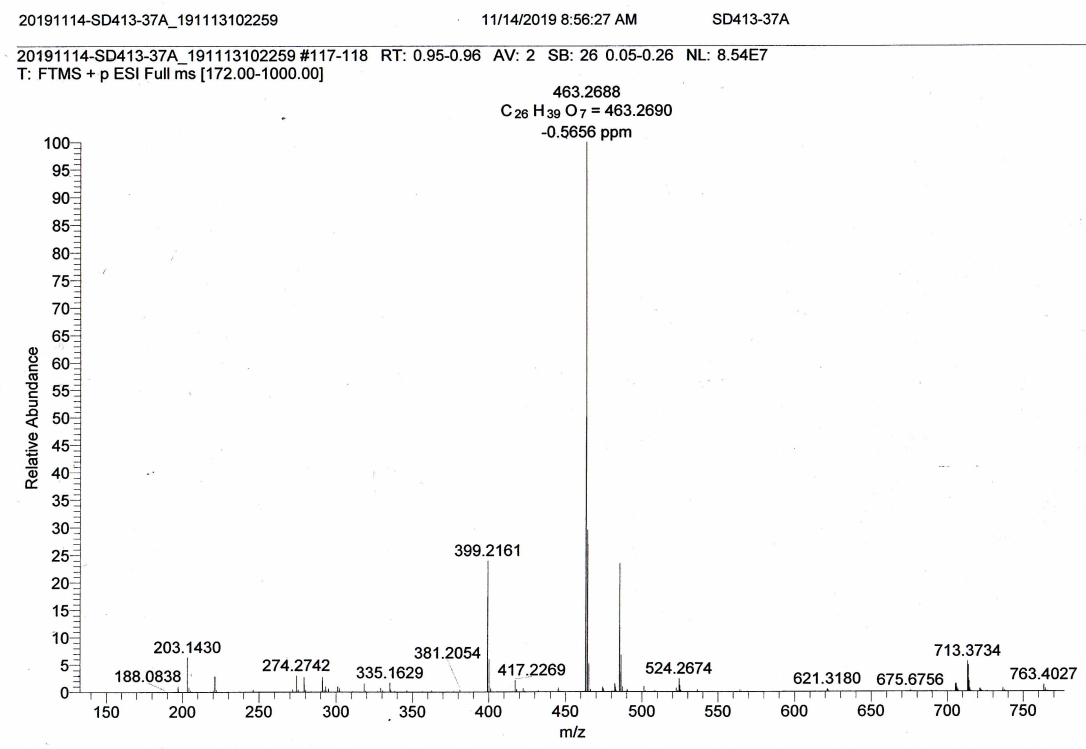


**Fig. S37.** ^1^H NMR spectrum of compound **3a** (Recorded in DMSO-*d*_6_);


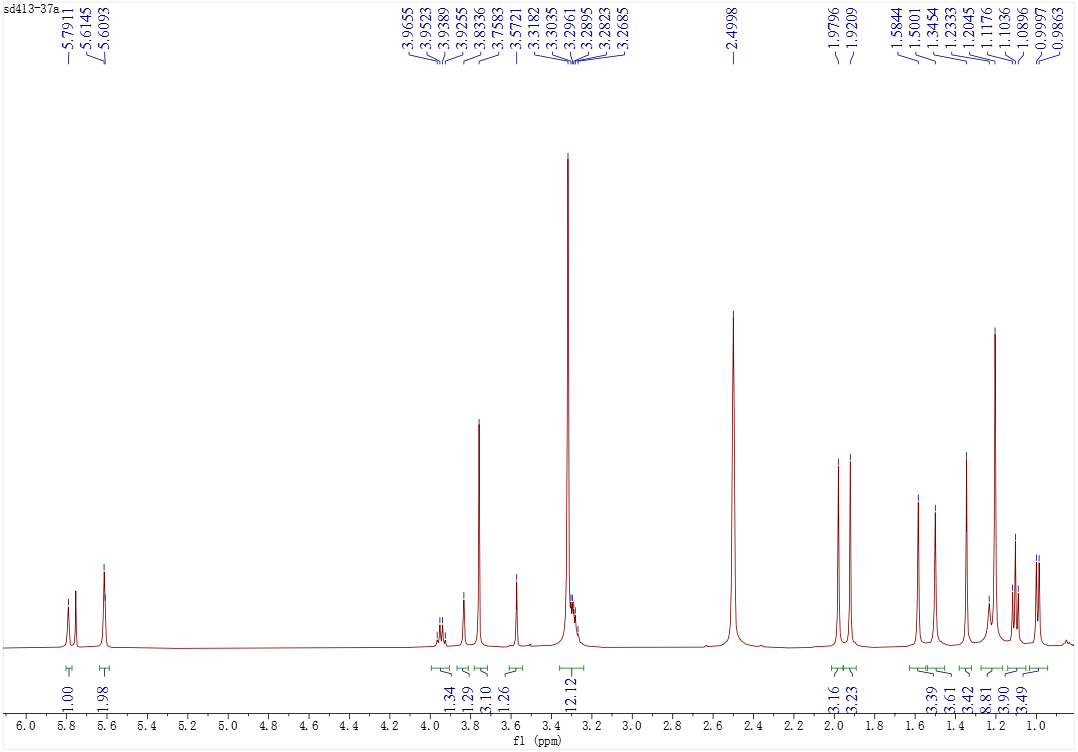


**Fig. S38.** ^13^C NMR and DEPT spectra of compound **3a** (Recorded in DMSO-*d*_6_);


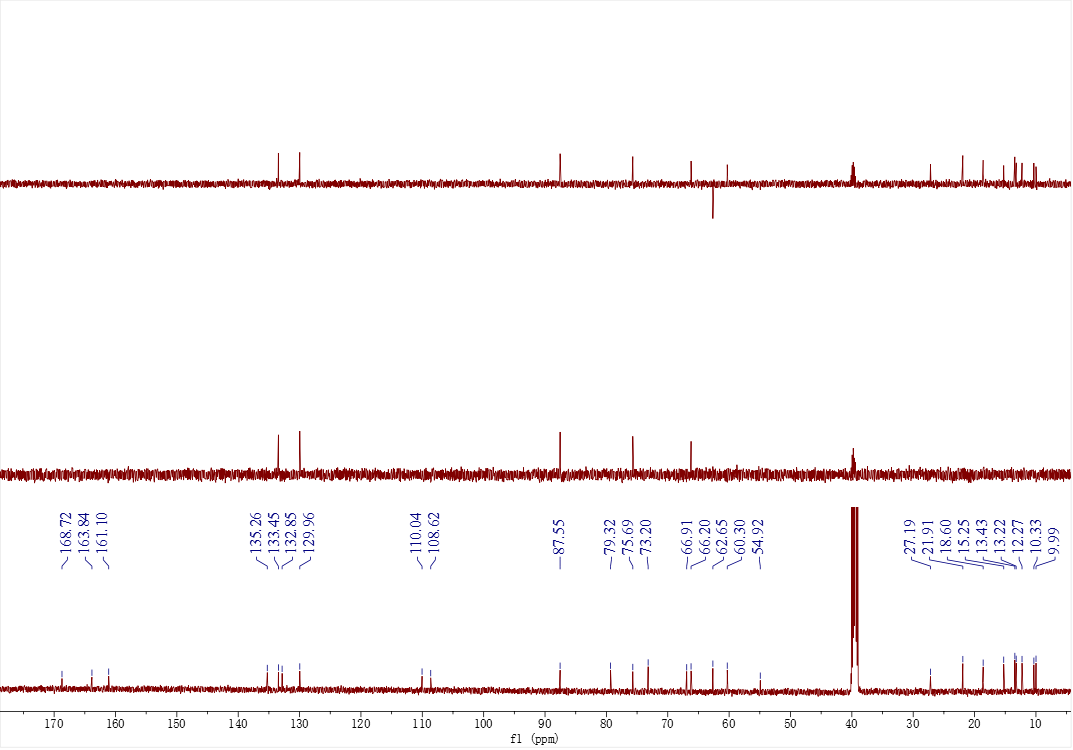


**Fig. S39.** NOESY spectrum of compound **3a** (Recorded in DMSO-*d*_6_);


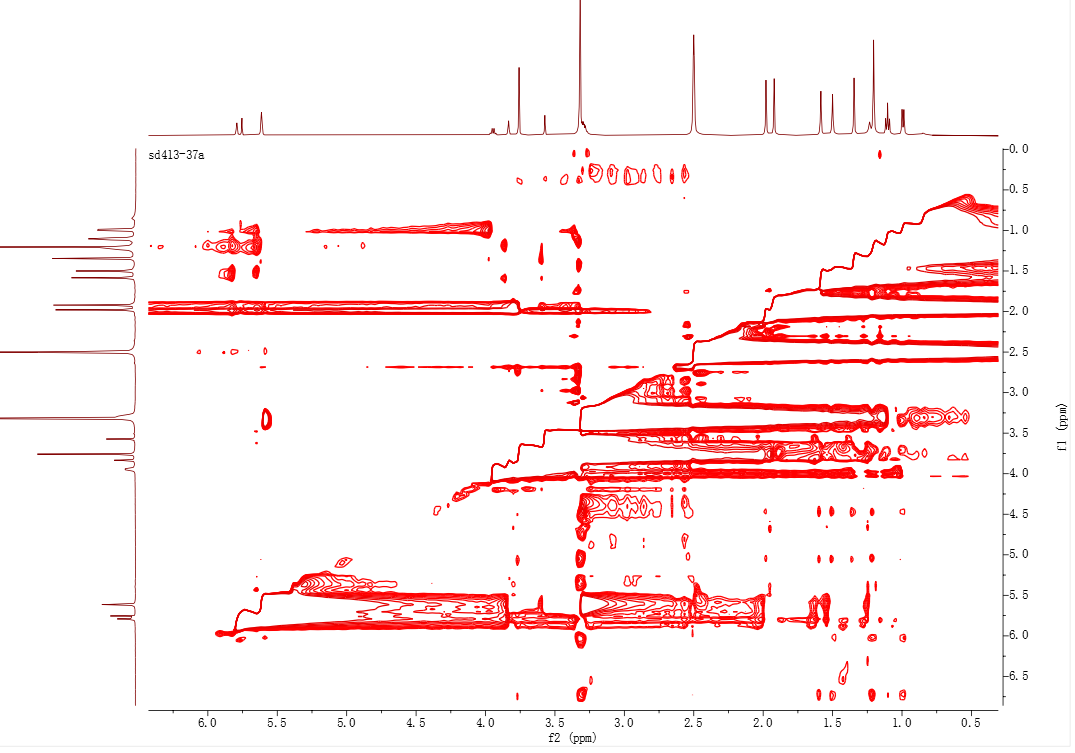


**Fig. S40.** HRESIMS spectrum of compound **3b**;


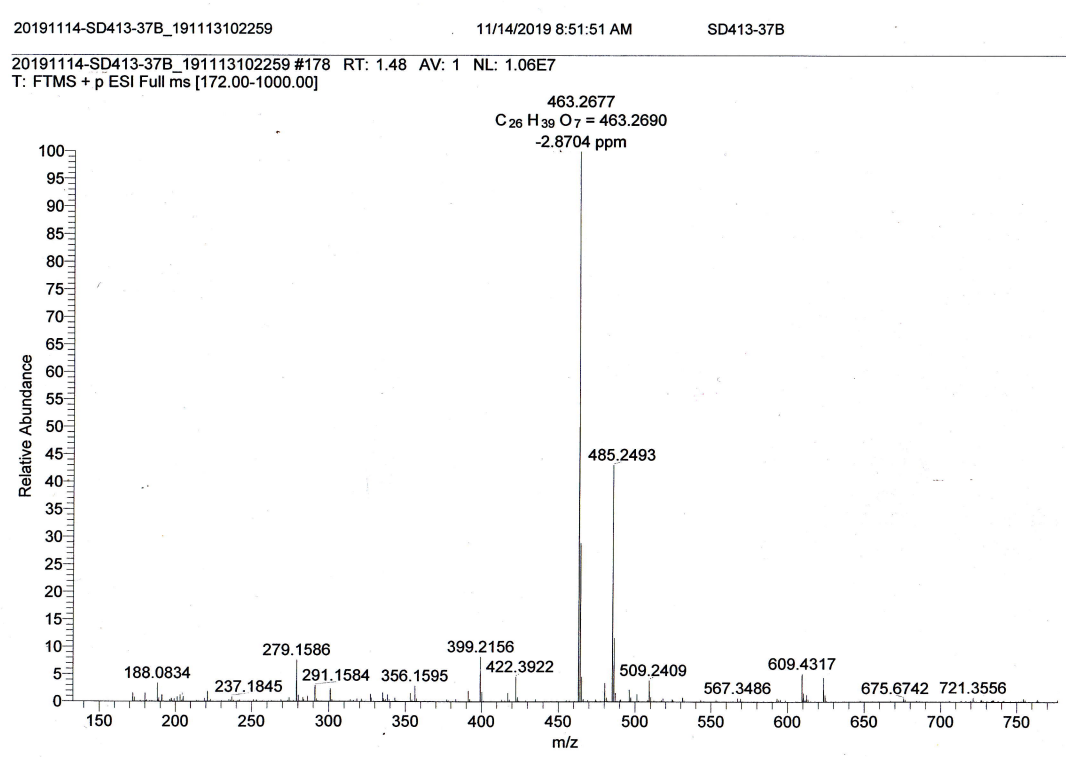


**Fig. S41.** ^1^H NMR spectrum of compound **3b** (Recorded in DMSO-*d*_6_);


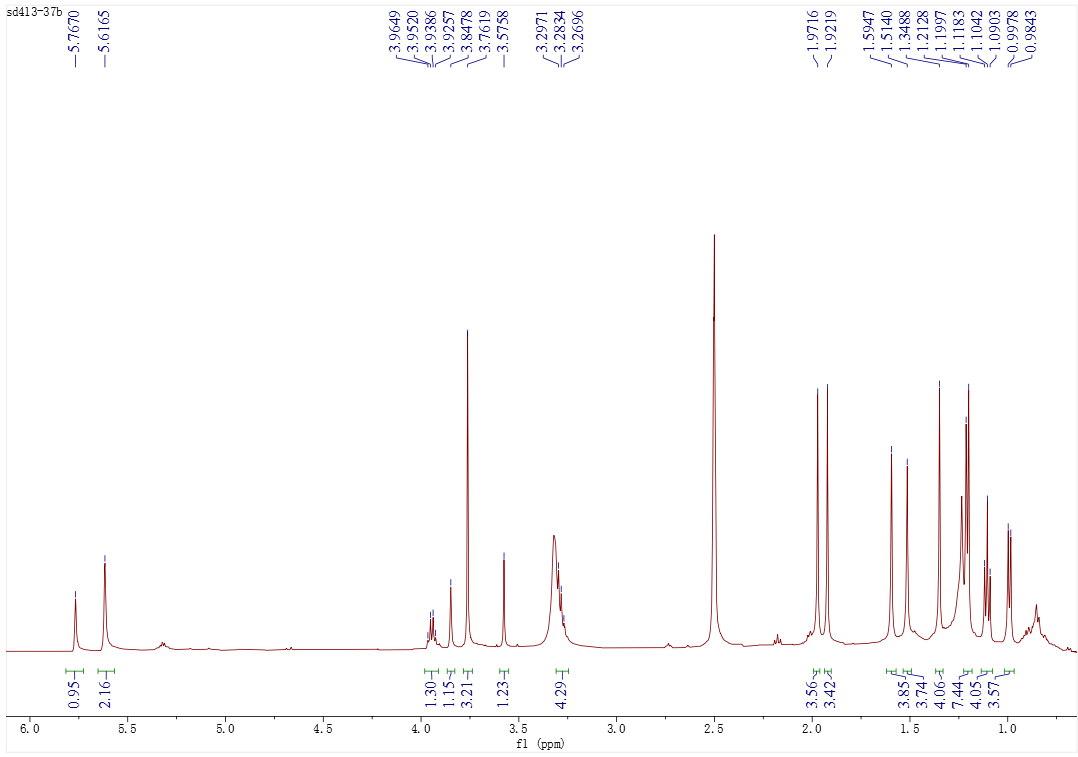


**Fig. S42.** ^13^C NMR and DEPT spectra of compound **3b** (Recorded in DMSO-*d*_6_);


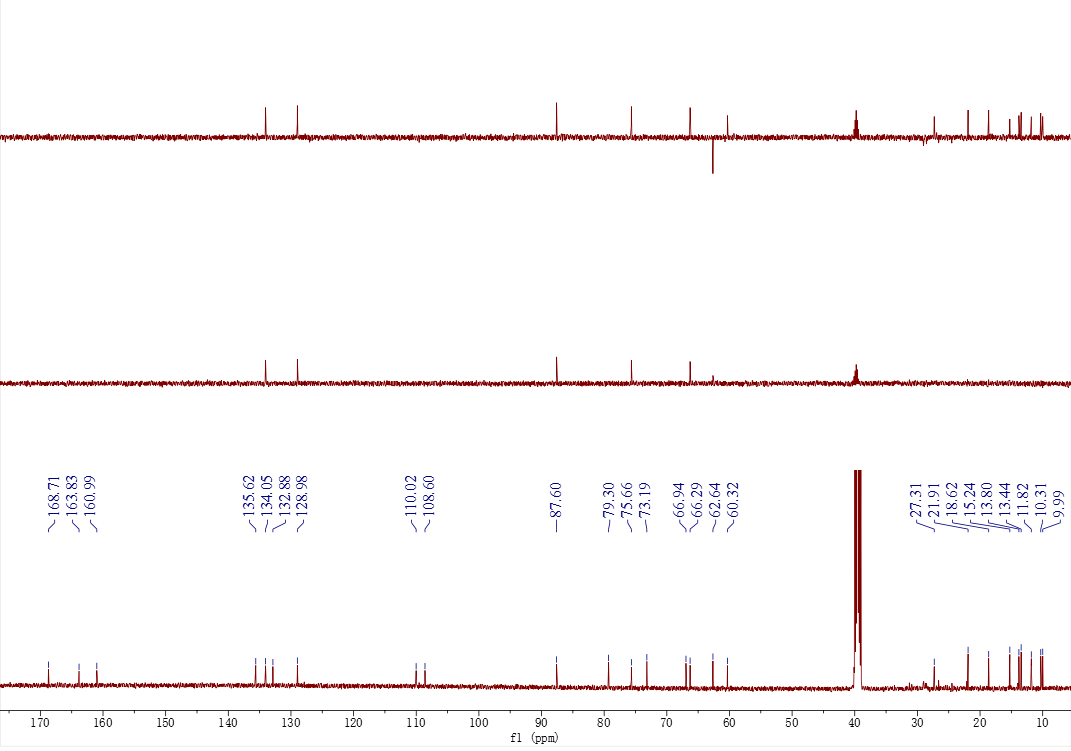


**Fig. S43.** NOESY spectrum of compound **3b** (Recorded in DMSO-*d*_6_);


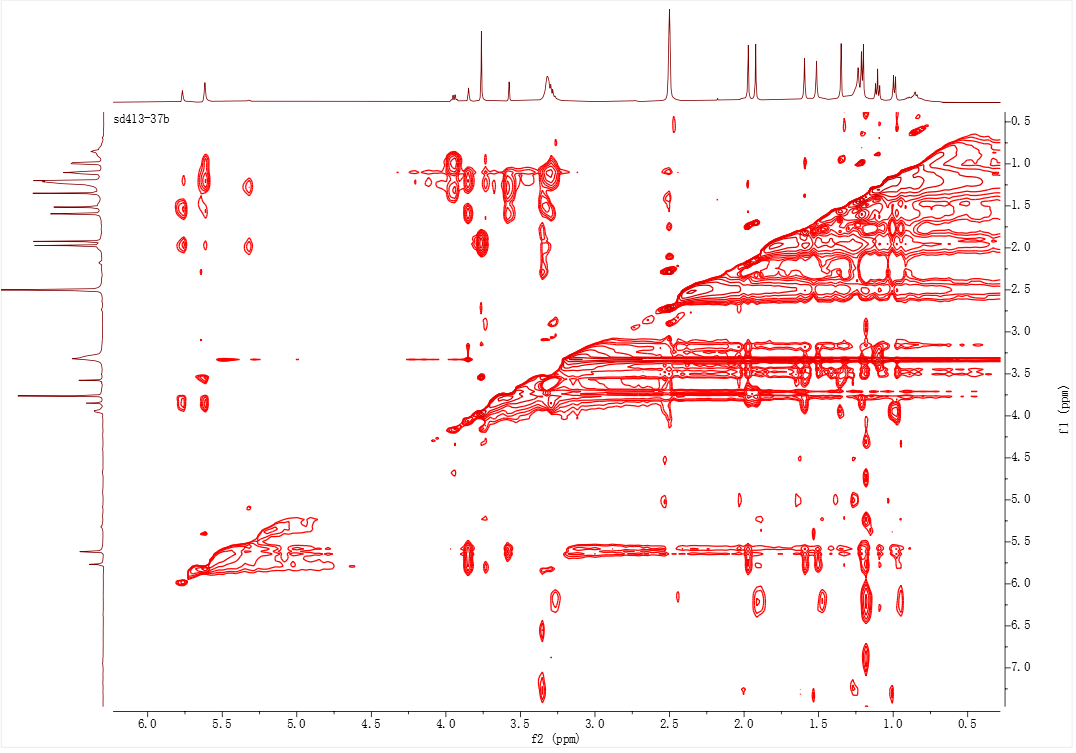


**Fig. S44.** ^1^H NMR spectrum of compound **4** (Recorded in DMSO-*d*_6_);

**Fig. S45.** ^13^C NMR and DEPT spectra of compound **4** (Recorded in DMSO-*d*_6_).

**Fig. S46.** Experimental ECD spectra of (6*R*,9*R*,12*S*,13*S*,14*R*,15*R*)-**1a** in MeCN compared with the Boltzmann-weighted B3LYP/TZVP PCM/MeCN, BH&HLYP/TZVP PCM/MeCN, CAM-B3LYP/TZVP PCM/MeCN and PBE0-TZVP PCM/MeCN spectra of (6*S*,9*R*,12*S*,13*S*,14*R*,15*R*)-**1a**.


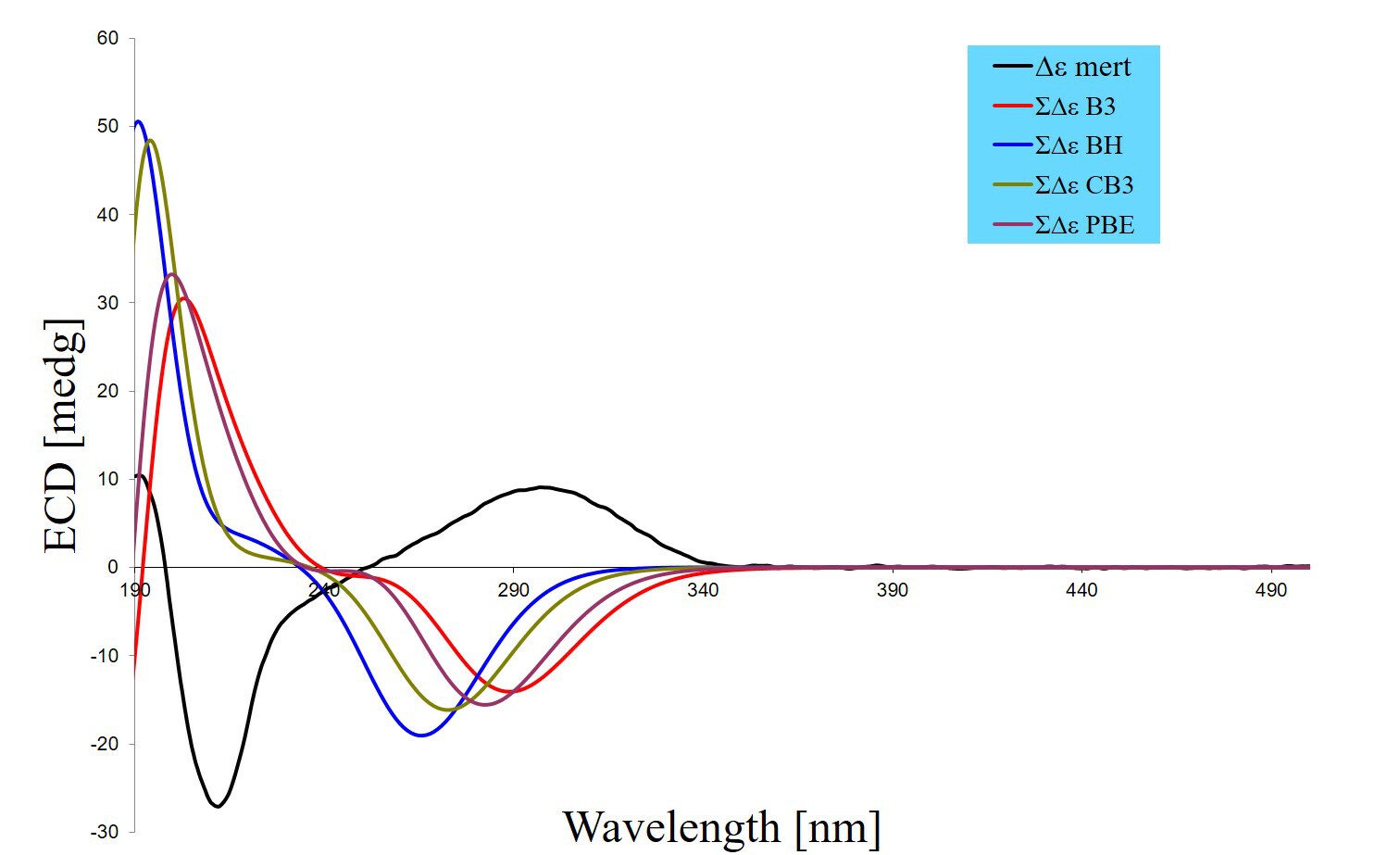


**Fig. S47.** Chiral HPLC chromatogram of compound **3** (Column: CHIRALPAK IG, 4.6 × 250 mm; Temperature: 25 ^o^C; Flow rate: 1 mL/min; Mobile phase: 80% *n*-hexane-isopropanol).


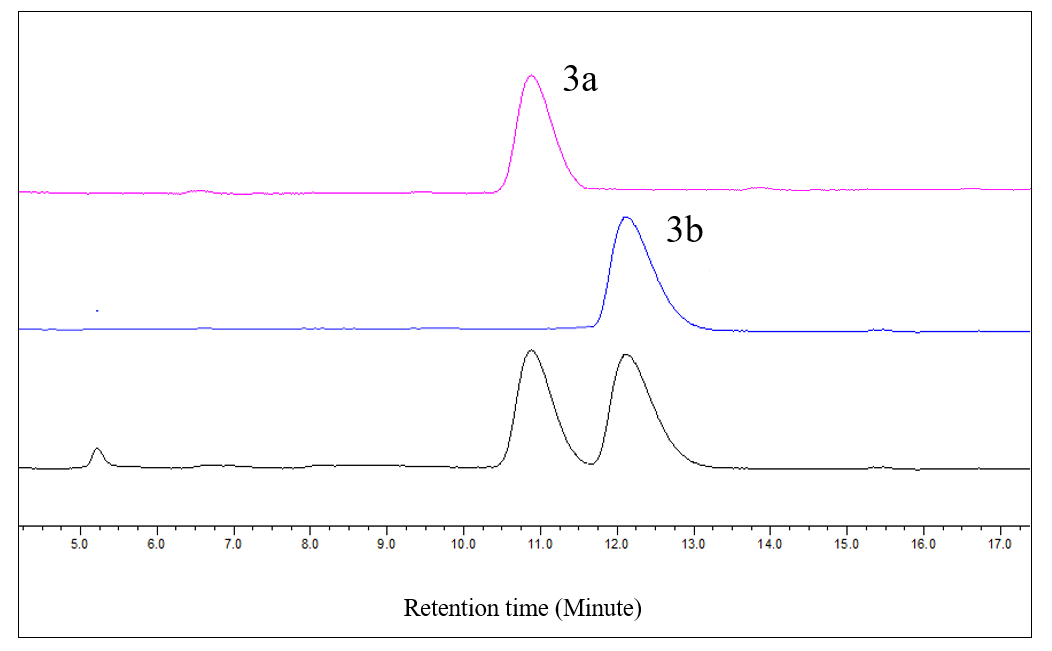


**Fig. S48.** COSY and key HMBC correlations of compound **3**.
